# The genome and proteome of *Serratia* bacteriophage η which forms unstable lysogens

**DOI:** 10.1186/1743-422X-11-6

**Published:** 2014-01-16

**Authors:** Jenna M Denyes, Peter J Krell, Richard A Manderville, Hans-Wolfgang Ackermann, Yi-Min She, Andrew M Kropinski

**Affiliations:** 1 Department of Molecular & Cellular Biology, University of Guelph, Guelph, ON N1G 2W1, Canada; 2 Department of Chemistry, University of Guelph, Guelph, ON N1G 2W1, Canada; 3 Department of Microbiology, Immunology, and Infectiology, Faculty of Medicine, Laval University, Quebec, QC G1X 4C6, Canada; 4 Shanghai Center for Plant Stress Biology, Chinese Academy of Sciences, 3888 Chenhua Road, Shanghai 201602, China; 5 Laboratory for Foodborne Zoonoses, Public Health Agency of Canada, 110 Stone Road West, Guelph, Ontario N1G 3W4, Canada; 6 Current address: ETH Zurich, Institute of Food, Nutrition and Health, Schmelzbergstrasse 7, 8092 Zurich, Switzerland

**Keywords:** *Serratia marcescens*, Phage evolution, Genome, Proteome, Bioinformatics, Lysogeny, Unstable lysogeny, Modified nucleosides, *Siphoviridae*

## Abstract

**Background:**

*Serratia marcescens* phage η is a temperate unclassified member of the *Siphoviridae* which had been reported as containing hypermodified guanine residues.

**Methods:**

The DNA was characterized by enzymatic digestion followed by HPLC analysis of the nucleoside composition, and by DNA sequencing and proteomic analysis. Its ability to form stable lysogens and integrate was also investigated.

**Results:**

Enzymatic digestion and HPLC analysis revealed phage η DNA did not contain modified bases. The genome sequence of this virus, determined using pyrosequencing, is 42,724 nucleotides in length with a mol% GC of 49.9 and is circularly permuted. Sixty-nine putative CDSs were identified of which 19 encode novel proteins. While seven close genetic relatives were identified, they shared sequence similarity with only genes *40* to *69* of the phage η genome, while gp1 to gp39 shared no conserved relationship. The structural proteome, determined by SDS-PAGE and mass spectrometry, revealed seven unique proteins. This phage forms very unstable lysogens with its host *S. marcescens.*

## Background

*Serratia marcescens* is a Gram-negative opportunistic human pathogen belonging to the family *Enterobacteriaceae*. In humans it is of particular concern for patients with indwelling medical devices [1]. Outbreaks are almost exclusively caused by contaminated medical solutions or equipment, with *S. marcescens* even growing in antiseptic solutions [2]. Only recently, community acquired infections have been identified [3,4], which highlight the growing importance of this pathogen. Treatment of infections is possible with combinations of antibiotics; however the number of clinical strains resistant to multiple antibiotics is on the rise [5,6], including isolates which produce extended spectrum beta-lactamases [7,8]. Phage therapy might prove a viable alternative, but historically phages infecting *S. marcescens* were isolated solely for use in bacterial typing systems [9,10] or for the transductional mapping of the genome [11,12] and their biology remains poorly understood [13].

There are 87 named *Serratia* bacteriophages listed in Bacteriophage Names 2000 (http://www.phage.org/names/2000/) belonging to the *Siphoviridae*[14], *Myoviridae*[15], and *Podoviridae*[12] families of tailed phages. Since 2000, only a limited number of additional *Serratia* phages have been isolated and characterized. Phages PPV [16] and KSP100 [17] belong to family *Podoviridae*; the former being a tentative member of the *T7likevirus* genus, while the latter KSP100 is a member of the *Phieco32likevirus* genus. Only one additional siphovirus, phage SM701, has been described [18]. All other newly characterized phages are all members of the *Myoviridae*. These include the virulent generalized transducing phage ϕIF3 (phiIF3) [11], ϕMAM1 (phiMAM1) [19] which is related to the newly proposed *Viunalikevirus*[20], and phages KSP20 and KSP90 [17]. Phylogenetic analysis based upon limited sequence data suggests that KSP20 with its 32 kb genome is part of the *Peduovirinae*[21], while KSP90 is part of the T4 superfamily.

*S. marcescens* bacteriophages were chosen as the focus of our research because of the growing importance of the host and its increasing prevalence as a nosocomial pathogen. Bacteriophage η was selected for further study. This virus was isolated from an overnight culture supernatant of *S. marcescens* ES, in 1960 in Germany [22]. Of considerable interest was von Lohr’s observation that the phage DNA displayed a discrepancy between the mol% GC calculated from melting temperature (Tm) and buoyant density determinations suggesting the presence of a hypermodified base. Preliminary data suggested a hypermodified guanine [22].

## Resrults and discussion

### Phage morphology and basic biology

**Figure 1:**
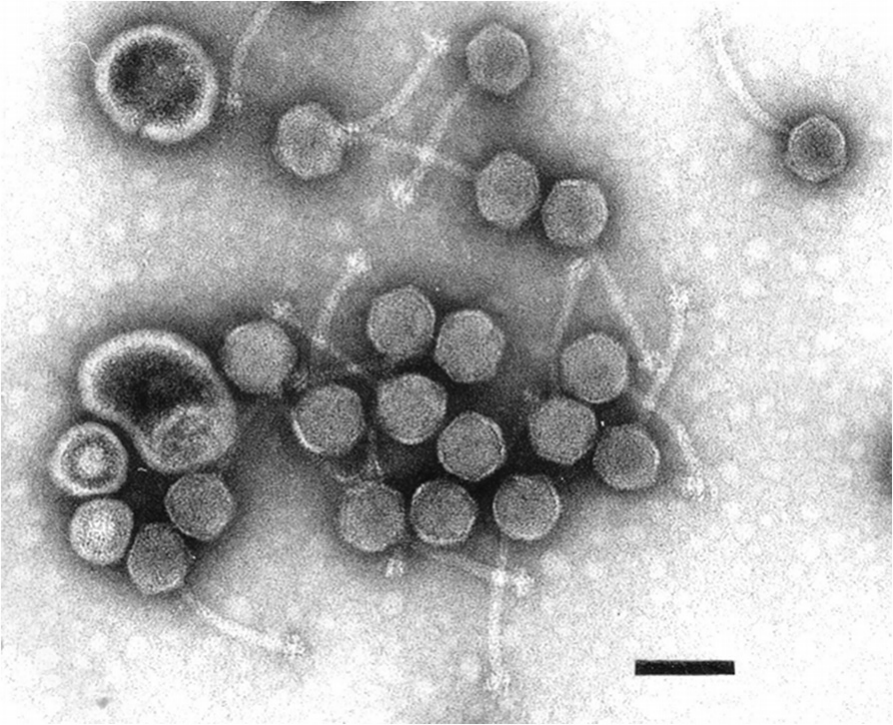
**Electron micrograph of phage η negatively stained with 2% uranyl acetate.** The bar indicates 100 nm.

Phage η is a B1 siphovirus [23,24] with an icosahedral head of 60 nm and a tail of 112 × 8 nm which ends in a base plate with three conspicuous spikes (Figure 1). As such, phage η is morphologically identical to the Jersey species of *Salmonella* phages [23,24].

The phage produces variably sized plaques when grown on *S. marcescens* using the double-agar overlay technique. The larger size plaque was approximately 3 mm in diameter, and the smaller only 1.5 mm. Both plaque types were turbid, with greater turbidity in the plaque centers. The distribution of turbidity in both plaque types was the same and they arose equally from subcultures of both small and large plaque types.

### Phage η forms highly unstable lysogens with its host

Phage-insensitive bacterial strains were selected from turbid plaques and serially propagated to remove any traces of contaminating exogenous phage. Lysogenic strains were distinguished from resistant strains by testing for the ability of chloroform-treated culture supernatants to form plaques on the wild type strain, for resistance to super-infection, and PCR reactions with phage η specific primers (data not shown). Initial subcultures were resistant to superinfection, spontaneous release of phage in absence of an induction agent to a titre of 10^7^ PFU/ml, and 10^9^ PFU/ml when induced with mitomycin C. In addition, phage-specific amplicons were observed with host DNA preparations. Most of the progeny from subsequent subcultures were phage sensitive, though phage production was also still measurable in the bulk culture. This combination of phage production and sensitivity suggests the sub-cultures were reverting to non-lysogenic cells. We choose to dismiss the possibility that this phage displays pseudolysogeny [15,25,26] based upon the isolation history of this virus and its inducibility by Mitomycin C; and, favour unstable lysogeny under laboratory culture conditions. Similar observations have been made previously with some *Vibrio* and *Clostridium* phages [27-29].

### Phage η DNA does not contain hypermodified guanine residues

Previous research had suggested the presence of a modified base in the DNA of phage η [22]. This was investigated by digestion of the DNA to nucleosides using a combination of DNase I, phosphodiesterase I and alkaline phosphatase coupled with HPLC separation of the products. We expected to observe a shift in the elution time of one of the four nucleoside peaks or the appearance of a fifth peak in the elution profile [30]. Neither of these were observed (Additional file 1: Figure S1), therefore there was no evidence to support the presence of a modified base. Phage molecular biologists no longer routinely calculate the mol% GC based upon buoyant density or melting temperature [30] and restriction analyses are also becoming a thing of the past. As a consequence there is a strong possibility that DNA modifications may not be discovered since pyrosequencing reads through most modifications (Kropinski, unpublished observations). Third generation sequencing techniques may address this potential problem.

### Phage η DNA is circularly permuted and terminally redundant

Determination of the physical location and nature of the genome ends can offer insight into the mechanisms of DNA replication and packaging that a bacteriophage employs [31]. Restriction digestion profiles of phage η DNA matched *in silico* predictions of a circular DNA molecule, and there was no difference in the profiles generated with ligated and unligated DNA samples (Additional file 2: Figure S2). Furthermore, there was no evidence of sub-molar fragments. Lastly, pulsed-field gel electrophoresis revealed one DNA band with no smearing or concatemeric molecules. These all suggest the impossible - the genome of phage η is circular, necessitating further study.

**Figure 2:**
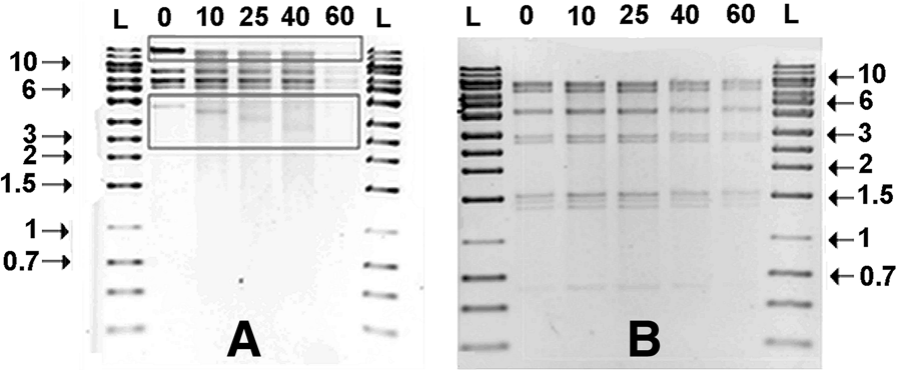
**Determination of the nature of the termini in phage η DNA. A**. Bal31 exonuclease treated phage λ DNA with subsequent EcoRI restriction digestion, Lanes are labeled according to the number of minutes the DNA was incubated with the Bal31 before EcoRI digestion with the bands which decrease are boxed. **B**. phage η DNA was digested with exonuclease and subsequently digested with EcoRI. All lanes labeled L in both 2A and 2B are loaded with Ultra Ranger DNA Ladder ™ with size markers indicated in kb.

Exonuclease digestions were carried out to processively truncate the DNA. When carried out before standard restriction digestion, the DNA bands containing the ends of the genome would be expected to become progressively smaller with increased exonuclease digestion time [31,32]. Time-controlled Bal31 exonuclease treatment followed by complete restriction digestion did not result in the decrease in molecular weight of any of the DNA restriction fragments (Figure 2A), In contrast for the control λ DNA, band mass of the left (band X) and right (band Y) termini were progressively reduced (Figure 2B). Extended Bal31 exonuclease treatment caused a decrease in η DNA concentration over time. As the exonuclease only acts on linear DNA this suggests that the η DNA molecule is linear, as expected, and not a covalently closed circle as evidenced by the restriction mapping. As an additional approach, 16 overlapping primer pairs were designed to span the genome and give products of approximately 4 kb. Each of the primer pairs produced a unique length PCR product which was the same size as predicted. The sum of these data suggests that the dsDNA genome is linear with a high degree of circular permutation.

### Genomic organization and annotation

The genome of phage η is 42,724 bp in length with a mol% GC of 49.9. This value is significantly less than that of the host, *S. marcescens* at 58 mol% GC, which is somewhat unusual for temperate phages where the overall base composition of the host and phage are usually remarkably similar (Kropinski, unpublished results). A unique 19 bp direct repeat was identified (5′-ATTGCAACTTATTTGTTTA-3′), which was found in four locations in the genome (Additional file 3: Table S1), and is possibly involved in regulation of expression.

**Figure 3:**
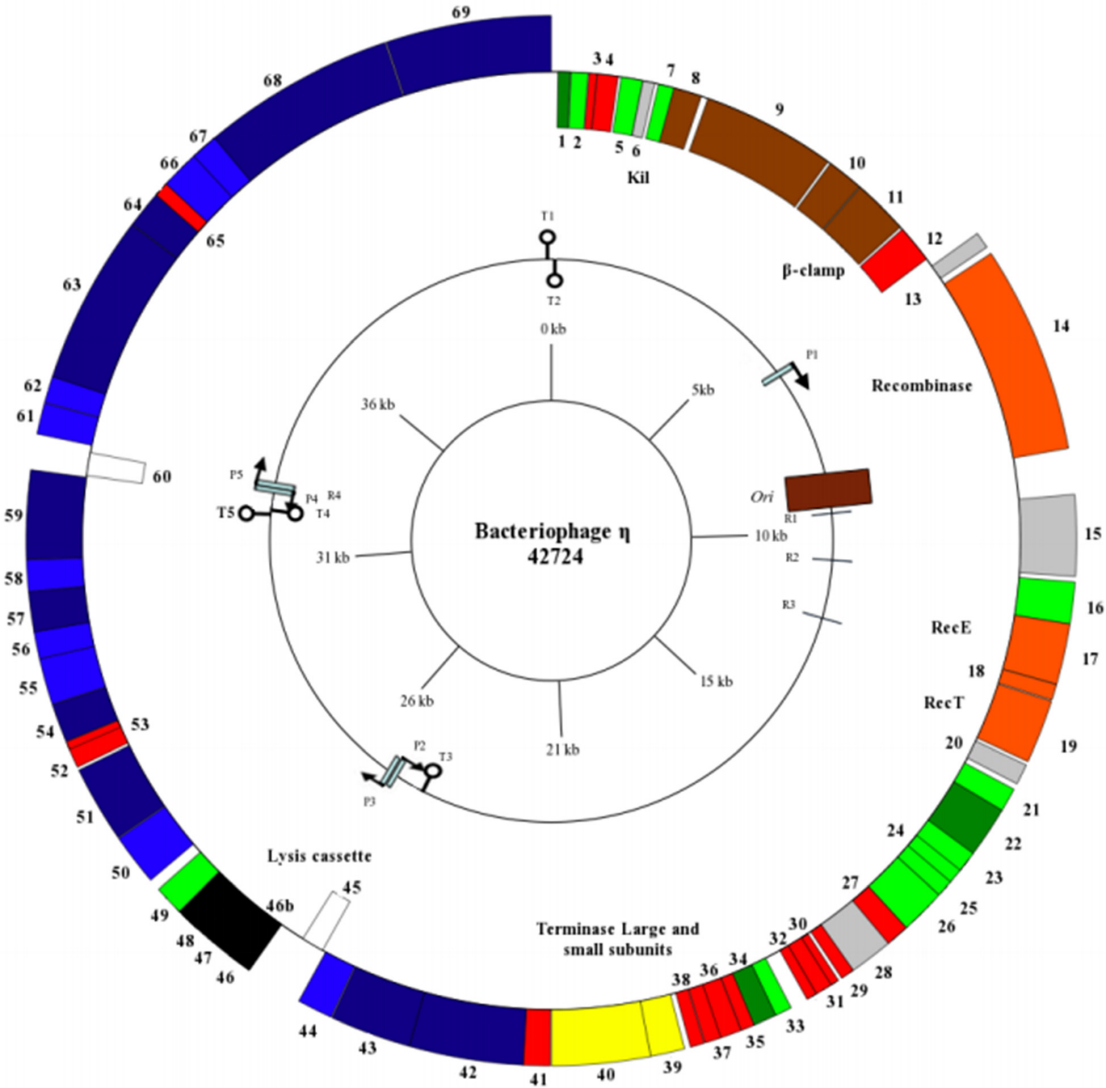
**Genetic map of η.** The genes are color coded according to function with red: novel, light green: prophage or bacterial related, dark green: phage related, black: lysis cassette, orange: recombination, brown: replication, yellow: packaging related, dark blue; confirmed structural, light blue: hypothetical, white: morons, grey: possible DNA binding and Kil. Promoters (P), terminators (T), direct repeats (R) and the *ori* are also included. Promoters are indicated with black arrows on stalks, while rho-independent terminators are indicated . The GenomeVx software package was used to construct this figure.

The orientation of genome annotation was chosen so that the majority of the genes were on the plus strand. Sixty-nine putative CDSs, identified during the genome annotation, are divided into four operons by four promoters and rho-independent terminators (Figure 3). Two potential morons (genes *45* and *60*) also divide the operons, each associated with their own promoter and terminator [33]. A putative *oriC* was identified using Ori-Finder.

The 69 proteins were grouped into six categories based on the number of homologs and the degree of amino acid conservation. Thirteen predicted proteins (90-100% identical sequence coverage) were very highly conserved to bacterial proteins with no homologs present in the viral database (data not shown). Nineteen predicted proteins were entirely novel. There were seven structural proteins confirmed using mass spectrometry (Additional file 4: Table S2). The fourth category contained 25 proteins which were conserved hypothetical proteins. Though based on genome organization of phage η and related phages, a structural or morphogenesis role could be hypothesized for nine of these proteins. Of the proteins with no predicted function, four DNA-binding proteins were tentatively identified, none of them related to previously characterized phage regulatory proteins. Functional predictions could be made for the remaining 13 gene products. These functions were associated mainly with the lytic replication cycle, with protein functions involved in DNA replication, DNA packaging, structural proteins and host cell lysis. No integrase was identified during the annotation, although recombination related proteins and putative DNA-binding proteins could be identified (Additional file 3: Table S1). Some notable genes will be discussed in greater detail.

### Phage integration

There are two lysogenic pathways open to temperate phages - integration into the host genome or maintenance as a plasmid. The former, as exhibited by coliphage lambda, involves recombination between homologous integration (*att*) sites on the phage and host genome mediated by integrase. Integration can also involve, as it does with the transposable phage such as Mu, a transposase, leading to nonhomologous recombination, and random insertion into the host genome. Phage Eta lacks both integrase and transposase homologs. The genome of this virus shares a 33 bp sequence (GTGGAGGTGCGTGATGCCAGCAAA TGAACTGAA) located between residues 15009 and 15041 within the complete genome sequences of *Serratia* sp. AS13 (CP002775), *Serratia* sp. AS12 (CP002774) and *Serratia plymuthica* AS9 (CP002773). In each case, the homologous sequence is located within a prophage related to enterobacterial phage cdtI identified using *PHAST*[33]. In the case of strain AS13, this site was within the coding sequence of hypothetical protein SerAS13_3448. Whether this serves as an integration site is not known; however, it is long enough that it could function in homologous recombination.

The alternative strategy is displayed by coliphage N15 [34], *Yersinia* phage PY54 [35], *Klebsiella* phage ΦKO2 [36], and *Halomonas aquamarina* phage ΦHAP-1 [37]. These phages encode protelomerase, along with partitioning proteins ParA and ParB. Coliphage P1 also possesses ParAB homologs. A ParB homolog was identified in Eta (gene *11*; Additional file 3: Table S1), though we can find no evidence for a ParS sequence nor a ParA homolog. It is possible that the prophage is maintained as a plasmid-type element, and is segregated using a combination of the phage ParB homolog (centromere-binding protein) and host factors.

### DNA packaging

Packaging of phage DNA into the precapsid during phage replication is carried out by the phage-encoded terminase holoenzyme, which is typically a heterodimer composed of large and small subunit. The large subunit of the terminase (gene *40*) of phage η was well conserved with previously identified subunits (Additional file 3: Table S1), unlike the small subunit (gene *39*), which had an entirely novel N-terminus. Basic features and domain organization in terminase enzyme complexes are shared in the tailed phages [38]. The large terminase subunit responsible for the ATPase and nuclease packaging-associated enzymatic functions, with the small terminase is involved in sequence specific substrate recognition and holoenzyme formation [39].

### Structural proteins

Seven distinct bands in the SDS-PAGE gel of phage η were identified using high resolution UPLC LTQ-FT mass spectrometry (Additional file 4: Table S2). Reliable identification was obtained by mass measurements of the peptides and high protein sequence coverage of the proteolytic fragments observed by tandem mass spectrometric (MS/MS) analyses. In the case of tailspike protein, the N-terminus of the protein was modified by acetylation (Additional file 4: Table S2). In addition to these high abundance proteins, an additional nine proteins could be annotated as likely as structural or morphogenesis proteins (Additional file 3: Table S1). It is possible that these proteins are either present at low abundance which could not be detected on the SDS-PAGE gel. While otherwise a standard lysis cassette, that of phage η is unique in that it is located in the middle of the capsid proteins with confirmed structural proteins both upstream and downstream (Figure 3). Typically, the structural proteins are co-regulated in one operon, with the lysis cassette upstream, which allows for a simplified regulation.

### Genomic relatives and classification

The structural cassette of phage η is the only segment of the genome which showed any degree of conserved relationship with phage genome sequences available in the NCBI database. Discontiguous megablast analysis revealed that phage η shared limited similarity (<22% sequence coverage) with *Escherichia* phages K1H [40] and K1G [40], *Salmonella* phages SETP3 [41], vB_SenS-Ent1 [42], SE2 [43], wksl3 [44], and SS3e [45]. The homologous regions were restricted to 22-43kb on the η genome, with the average shared sequence identity above 80% (Additional file 3: Table S1).

## Conclusions

*Serratia* phage η was originally characterized as a temperate siphovirus possessing hypermodified guanine residues in its DNA. The putative presence of hypermodified bases in a temperate phage creates a conceptual problem since expression of modifying enzymes would probably modify the host genome with catastrophic results. Our chromatographic results do not support the existence of modified bases in the DNA of this virus. Furthermore, the genomic results suggest that this phage is not classically temperate, since it lacks a demonstrable repressor and integrase homologs, but is capable of establishing an unstable relationship with its host which can best be described as unstable lysogeny. The DNA genome of phage η is terminally redundant and highly circularly permuted. The latter observation has led other researchers [19,46-49] to the mistaken assumption that their phage genomes are circular, which has never been observed among the *Caudovirales*, and indeed would pose problems in DNA packaging.

Comparative genomic analysis reveals that *Serratia* phage η is a unique virus which, except for the structural gene module, is unrelated to any of the current population of phages which have the complete genomes sequenced. Interestingly, the genome of its structural homolog, *Salmonella* phage Jersey, has recently been sequenced as part of a project to characterize the “Jerseylikevirus” genus (H. Anany, personal communication), and the only relationship between these viruses is within the morphogenesis cassette.

## Materials and methods

### Bacteria and bacteriophages

*S. marcescens* CV/rc3 (HER 1311) and phage η were obtained from the Centre de Référence pour les Virus Bactériens Félix d’Hérelle (Université Laval, QC, Canada). The host was grown in Difco Luria-Bertani broth (LB; Fisher Scientific, Toronto, ON, Canada) at 30°C with shaking at 180 rpm or on LB agar plates (LB with 1.5% [wt/vol] agar) at 30°C. The bacteriophage titer was determined using the double agar overlay method [48]. Phage particles were partially purified by PEG precipitation and continuous CsCl purification [49]. Purified phage was dialyzed using a Pierce 10 K Slide-A-Lyzer cassette (Thermo Fisher Scientific, Rockford, IL, USA) against 10 mM Tris-0.1 mM EDTA (pH 8.0) (TE) buffer to remove CsCl. Purified phage was stored at 4°C.

### Electron microscopy

Purified phages were sedimented by centrifugation at 25,000 g for 60 min, using a Beckman Coulter J-E (Palo Alto, CA) centrifuge and a JA19.1 fixed angle rotor. The pellet was washed twice under the same conditions in neutral ammonium acetate (0.1 M). Phages were deposited on copper grids with carbon-coated Formvar films, stained with 2% uranyl acetate (pH 4.5) and examined using a Philips EM 300 electron microscope.

### DNA isolation, digestion and HPLC analysis: Phage

DNA was extracted by standard phenol chloroform extraction and ethanol precipitation [50]. The purified phage η genomic DNA was digested to the nucleoside level in preparation for HPLC analysis according to the protocol described by [30] using DNase I, phosphodiesterase I and alkaline phosphatase (Worthington Biochemical Corp. Vassar, NJ, USA). The number of units of phosphodiesterase I were increased from 0.01 units in the protocol to 0.1 units. The phosphodiesterase I and alkaline phosphatase digestions were done in a water bath at 25°C. The digested DNA was filtered using an Ultrafree–MC membrane with a pore size of 0.1 μm (Millipore Corp, Billerica, MA, USA) to remove particles in preparation for loading onto the HLPC column. Standard nucleosides were purchased from Sigma-Aldrich (Sigma-Aldrich, Oakville, Canada).

The protocol developed was based on the method outlined by [30]. The analysis was performed on an Agilent 1100 series LC/MSD_SL Trap system. Samples (100 μl) were injected into the LC/MSD system through an Agilent 1100 series thermostated auto-sampler. Separations were carried out on a 5 μm Agilent ZORBAX SB C-18 column (4.6 × 150 mm) at a flow rate of 0.75 ml/min. Two mobile phase solvents were used; Solvent A was 0.1% formic acid in deionized water; Solvent B was 100% HPLC grade acetonitrile. Dimethyl sulfoxide (DMSO) was used to dissolve the concentrated nucleosides. HPLC method: 98/2 A/B for five minutes then increasing to 95/5 A/B at ten minutes, 80/20 A/B at 12 minutes then returning to 100% A.

### DNA sequencing and annotation

Phage DNA was digested, ligated and treated with Bal31 exonuclease according to manufacturer’s specifications (NEB) and resolved using a 1% agarose gel. Genome end determination was carried out following protocols published by Loessner *et al*., [32] and Casjens *et al*., [51]. Pulsed-field gel analysis was carried out following the protocol of Lingohr *et al*., [52].

The genome sequence was determined using 454 sequencing technology at the McGill University and Genome Quebec Innovation Centre (Montreal, QC, Canada) with 49.1 fold coverage. The genome was annotated using Kodon (Applied Maths, Austin, TX, USA) with the proteins screened for homologs using the BLASTP feature of Geneious Pro 6.2 (Biomatters Ld., Auckland, New Zealand). Their molecular mass and pI were calculated using the Batch MW and pI Finder server at http://greengene.uml.edu/programs/FindMW.html. Potential protein motifs were identified using the NCBI Batch CD-Search tool at http://www.ncbi.nlm.nih.gov/Structure/bwrpsb/bwrpsb.cgi? with an E-value threshold of 0.0001. Transmembrane domains were screened for using TMHMM [53] and Phobius [54]. In certain cases HHpred [55,56] was employed to analyze the protein sequences. The annotated sequence was deposited with GenBank under accession number KC460990 (GI:511624446). RNA secondary structure prediction was used to support the identification of rho-independent terminators [57]. The oriC was identified using Ori-Finder [58]. The gene diagram was constructed using Genome Vx [59].

### Structural protein analysis

Phage particles which had been purified through CsCl twice were used for SDS-PAGE structural protein analysis. Purified whole phages were denatured at 100°C for 5 min. in Laemmli Sample Buffer supplemented with 100 mM 2-mercaptoethanol. The structural proteins were resolved in a Bio-Rad Ready 5-15% Tris-HCl gel, in Running Buffer (25 mM Tris, 192 mM glycine, 0.1% SDS, pH 8.3 (Bio-Rad)) at 150 V for an hour. Visible bands were excised, and then digested in-gel by sequencing-grade trypsin. The cleaved peptide fragments of the proteins were extracted using acentonitrile/0.1% trifluoroacetic acid (TFA) (v/v, 60:40), then dried by a Savant vacuum centrifuge (Thermo Fisher Scientific, Nepean, Ontario). In the subsequent mass spectrometric analyses, the peptides were reconstituted in 8 μl of 0.2% formic acid (FA) and identified using a nanoAcquity ultra-performance liquid chromatograph (UPLC, Waters, Milford, MA) – linear ion-trap Fourier transform ion cyclotron resonance (LTQ-FT ICR, Thermo Fisher, San Jose, CA) mass spectrometer. The sample was normally trapped by a reverse-phase (RP) Symmetry C18 column (180 μm i.d. × 20 mm length, 5 μm) at 5 μl/min of solvent A (0.1% FA) for 3 minutes, and then separated through a C18 analytical column (100 μm i.d. × 100 mm, 1.7 μm, BEH 130) at 400 nl/min for 65 minutes. UPLC gradient was set up to a linear gradient from 5% to 30% solvent B (0.1% FA in acetonitrile), followed by to 85% solvent B over 10 min. for peptide elution. FT-MS scans were acquired with high resolution (100,000) MS from m/z 300 to 2000, and MS/MS measurements in linear ion-trap mode by data-dependent scans of the top eight intense precursor ions at multiply charged states of 2+, 3+ and 4+. Dynamic exclusion was set to a period of time at 180 s.

Protein identification was performed using Mascot Server (version 2.3.0, Matrix Science, London, UK), and UPLC MS/MS raw data were searched against the in-house database of the protein sequence derived from the annotated genome of phage η. The search parameters were restricted to tryptic peptides for a maximum of 2 missed cleavages. Cysteine carbamidomethylation was designated as a fixed modification, and deamidation of asparagine and glutamine, methionine oxidation were considered as variable modifications. Mass tolerances were set up to 10 ppm for the FT-MS ions and 1 Da for ion trap MS/MS fragment ions. Peptide assignments were filtered by an ion score cut off at 20, and the identified MS/MS spectra were also verified manually.

## Abbreviations

BLAST: Basic local alignment search tool; gp: Gene product; HPLC: High pressure liquid chromatography; N/A: Not applicable; CDS: Coding sequence; PFU: Plaque forming unit, a measure of the number of viable viral particles; SDS-PAGE: Denaturing polyacrylamide gel electrophoresis; TMHMM: Transmembrane prediction using hidden Markov models.

## Competing interests

The authors have no competing interests to disclose.

## Authors’ contributions

AMK and PJK designed and guided the project; JMD did most of the writing of the paper and the laboratory work and informatic analysis; RAM directed the HPLC nucleoside analysis; HWA did the electron microscopic examination of the phage; YMS preformed the mass spectrometry, all authors read and approved the final manuscript.

## Supplementary Material

Additional file 1: Figure S1 HPLC analysis Digested samples were separated using concentration gradients of H_2_O with 0.1% formic acid (Buffer A) and acetonitrile (Buffer B) which varied with time and are listed in Additional file 3 and in Figure 3. A mixture of the four unmodified nucleosides was used as a standard and the chromatogram produced by their separation was overlaid on the separation achieved with the experimental sample DNA. Peak elution times remained consistent and no new peaks were evident, supporting the conclusion that there is no modification present in the DNA. The small peak visible at 14 minutes was also fluorescent (fluorescence data not shown), and it was concluded to be a result of contamination of the column and unrelated to this experiment, as the concentration of acetonitrile was extremely high which would result in the elution of any contaminants. Absorbance was measured at 254 nm. Click here for file

Additional file 2: Figure S2 Restriction digestion of phage η DNA. Lane L contains Norgen UltraRanger DNA Ladder™, with size markers indicated in kb. The even numbered lanes contained un-ligated η DNA while ligated DNA was used as the substrate in the odd numbered lanes. Lanes 1 and 2 - digested with NdeI, 3 and 4 with BglI, and lanes 5 and 6 with EcoRI. Click here for file

Additional file 3: Table S1 List of CDSs, putative promoters and terminators found in phage η. An E-value threshold of 0.0001 was used for all homology analysis. Click here for file

Additional file 4: Table S2 An SDS-PAGE analysis of the purified structural phage proteins on a 12% SDS-PAGE separation gel alongside a PageRuler™ prestained protein ladder (Fermentas) is presented. Visible protein bands were excised from the gel, and labeled as shown in the figure. Subsequently the nature of the protein in the visible band was identified by UPLC-LTQFT -MS/MS analysis. For every detected protein the protein name, the predicted molecular size (Da), the maximum number of unique spectra and sequence coverage (%) is listed. Click here for file
