# Supplementary figures and images for: The genome and proteome of Serratia bacteriophage η which forms unstable lysogens
Source: Virol J. 2014 Jan 16;11:6. doi: 10.1186/1743-422X-11-6 (PMC3918226; doi:10.1186/1743-422X-11-6)

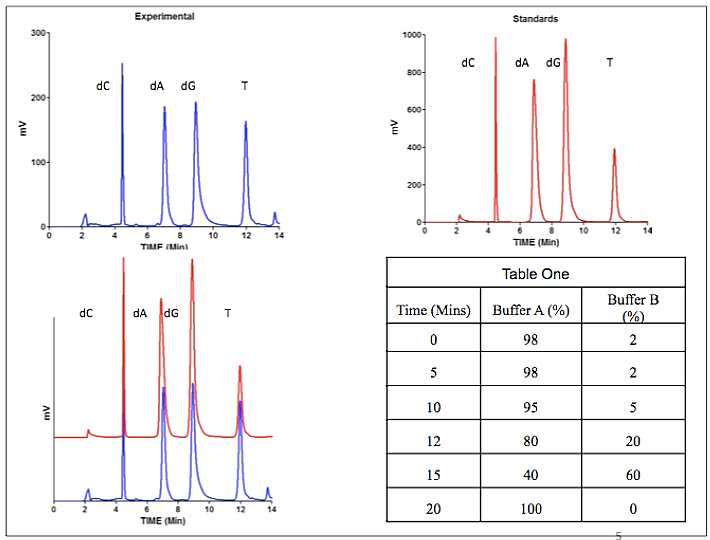

Supplement: Additional file 1: Figure S1 — HPLC analysis Digested samples were separated using concentration gradients of H2O with 0.1% formic acid (Buffer A) and acetonitrile (Buffer B) which varied with time and are listed in Additional file 3 and in Figure 3. A mixture of the four unmodified nucleosides was used as a standard and the chromatogram produced by their separation was overlaid on the separation achieved with the experimental sample DNA. Peak elution times remained consistent and no new peaks were evident, supporting the conclusion that there is no modification present in the DNA. The small peak visible at 14 minutes was also fluorescent (fluorescence data not shown), and it was concluded to be a result of contamination of the column and unrelated to this experiment, as the concentration of acetonitrile was extremely high which would result in the elution of any contaminants. Absorbance was measured at 254 nm. [file 1743-422X-11-6-S1.tiff]

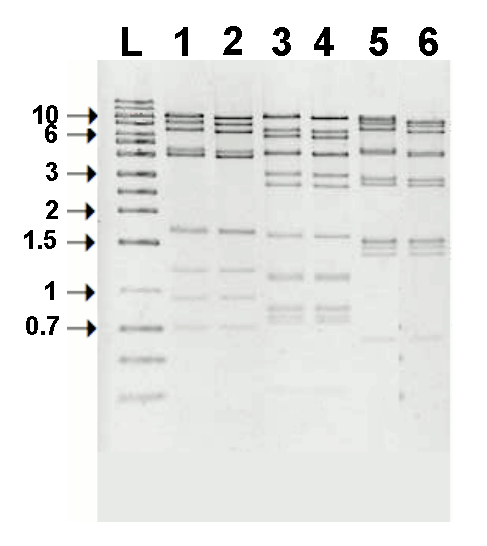

Supplement: Additional file 2: Figure S2 — Restriction digestion of phage η DNA. Lane L contains Norgen UltraRanger DNA Ladder™, with size markers indicated in kb. The even numbered lanes contained un-ligated η DNA while ligated DNA was used as the substrate in the odd numbered lanes. Lanes 1 and 2 - digested with NdeI, 3 and 4 with BglI, and lanes 5 and 6 with EcoRI. [file 1743-422X-11-6-S2.tiff]
