# Supplementary material for: The genome and proteome of Serratia bacteriophage η which forms unstable lysogens
Source: Virol J. 2014 Jan 16;11:6. doi: 10.1186/1743-422X-11-6 (PMC3918226; doi:10.1186/1743-422X-11-6)
Supplement: Additional file 3: Table S1 — List of CDSs, putative promoters and terminators found in phage η. An E-value threshold of 0.0001 was used for all homology analysis. [file 1743-422X-11-6-S3.doc]

**Additional file 3,** **Table S1.** List of CDSs, putative promoters and terminators found in η.

| Gene | Start | Stop | Strand | Mass (Da) | pI | Annotation | Homolog | E value | Protein Domain |
| --- | --- | --- | --- | --- | --- | --- | --- | --- | --- |
| T1 | 71 | 90 | - |  |  | Rho-independent terminator 1 | AAAGGGGCCTTTCGGCCCCTTTT | | |
| T2 | 75 | 97 | + |  |  | Rho-independent terminator 2 | AGGGGCCTTTCGGCCCCTTTTA | | |
| *1* | 97 | 276 | - | 6853 | 9.3 | conserved hypothetical protein | NP_112042 hypothetical protein HK620p09 [Enterobacteria phage HK620] | 1.08e-05 |  |
| *2* | 287 | 535 | - | 9408 | 8.9 | conserved hypothetical protein | YP_008126237 hypothetical protein VPRG_00074 [Vibrio phage pYD38-A] | 2.69e-21 |  |
| *3* | 548 | 664 | - | 4144 | 10.3 | novel protein | - | - |  |
| *4* | 668 | 973 | - | 11545 | 9.7 | novel protein | - | - |  |
| *5* | 1017 | 1331 | - | 11582 | 5.5 | novel protein | - | - |  |
| *6* | 1337 | 1510 | - | 6205 | 9 | Kil protein | YP_007151642 Kil protein [Enterobacteria phage HK544] | 5.90e-07 | pfam06301 Lambda kil |
| *7* | 1567 | 1821 | - | 9444 | 9.6 | conserved hypothetical protein | YP_001700551 hypothetical protein STM0899.1n.Fels1 [Salmonella phage Fels-1] | 4.99e-07 |  |
| *8* | 1797 | 2213 | - | 15097 | 9.2 | conserved hypothetical protein | YP_007002057 putative type III restriction endonuclease [Brucella phage Tb] | 2.28e-24 |  |
| *9* | 2314 | 4287 | - | 73859 | 7.3 | putative helicase | YP_007674015 helicase [Vibrio phage pYD21-A] | 4.35e-148 | pfam00270 DEAD/DEAH box helicase |
| *10* | 4330 | 4869 | - | 20083 | 8.3 | putative beta-clamp protein | YP_008126172 hypothetical protein VPRG_00008 [Vibrio phage pYD38-A] | 1.04e-76 | cd00140 Beta clamp domain of DNA polymerase III |
| *11* | 4882 | 5703 | - | 30266 | 7.8 | DNA-binding protein - putative parB-like partition protein | YP_007112451 hypothetical protein mEp390_034 [Enterobacterial phage mEp390] | 3.08e-70 |  |
| *12* | 5743 | 6300 | - | 20436 | 4.6 | novel protein | - | - |  |
| P1 | 6373 | 6398 | + | - | - | Promoter 1 | TTGACGCGCAACCTTTTTGTTGTTAT | | |
| *13* | 6430 | 6648 | + | 7719 | 8 | novel protein | - | - |  |
| *14* | 6743 | 9478 | + | 99672 | 6 | DNA primase | NP_042036 DNA primase [Enterobacteria phage P4] | 2.11e-45 | cd01029 TOPRIM_primases |
| *Ori* | 9488 | 9759 |  | | | | | | |
| R1 | 10010 | 10026 | + | - | - | Direct Repeat 1 | AAACAAATAAGTTGCAA | | |
| *15* | 10079 | 11146 | + | 39356 | 5.3 | conserved hypothetical protein | YP_002284383 hypothetical protein PAJU2_gp49 [Pseudomonas phage PAJU2] | 1.73e-17 |  |
| R2 | 11145 | 11161 | + | - | - | Direct Repeat #2 | AAACAAATAAGTTGCAA | | |
| *16* | 11218 | 11763 | + | 20758 | 4.7 | conserved hypothetical protein | YP_008126194 hypothetical protein VPRG_00030 [Vibrio phage pYD38-A] | 3.56e-17 |  |
| *17* | 11764 | 12564 | + | 30295 | 5.6 | exonuclease VIII (RecE) | YP_007001958 hypothetical protein [Escherichia phage TL-2011b] | 1.63e-55 | PRK09709 RecE  exonuclease VIII |
| R3 | 12563 | 12579 | + | - | - | direct repeat #3 | AAACAAATAAGTTGCAA | | |
| *18* | 12569 | 12778 | + | 8278 | 6.8 | novel protein | - | - |  |
| *19* | 12794 | 13633 | + | 30874 | 4.7 | Bet (RecT) | YP_001449250.1 putative Bet protein [Phage BP-4795] | 9.27e-30 | TIGR01913 Lambda Bet recombination protein |
| *20* | 13682 | 13954 | + | 10061 | 10 | novel protein | - | - |  |
| *21* | 14035 | 14352 | + | 11310 | 4.7 | novel protein | - | - |  |
| *22* | 14349 | 15035 | + | 26321 | 5.3 | conserved hypothetical protein | YP_006561 PmgT [Enterobacteria phage P1] | 5.24e-40 |  |
| *23* | 15022 | 15303 | + | 10183 | 6.8 | conserved hypothetical protein | C-terminus YP_006906829.1|hypothetical protein [Escherichia phage P13374]] | 2e-09 |  |
| *24* | 15296 | 15496 | + | 7575 | 9.2 | conserved hypothetical protein | YP_004934064 hypothetical protein HK639_34 [Escherichia phage HK639 | 4.53e-13 |  |
| *25* | 15493 | 15717 | + | 8241 | 6.4 | conserved hypothetical protein | YP_004327518 putative uncharacterised protein [Salmonella phage ViI] | 1.79e-17 |  |
| *26* | 15717 | 16316 | + | 22095 | 5 | conserved hypothetical protein | YP_006560843 hypothetical protein AMBK_37 [Salmonella phage vB_SosS_Oslo] | 3.23e-04 |  |
| *27* | 16317 | 16640 | + | 12352 | 6.2 | novel protein | - | - |  |
| *28* | 16624 | 17205 | + | 21183 | 4.5 | putative EaA protein | YP_004934089 hypothetical protein HK639_59 [Escherichia phage HK639] | 1.53e-12 |  |
| *29* | 17198 | 17380 | + | 7182 | 4.8 | novel protein |  | - |  |
| *30* | 17431 | 17544 | + | 4332 | 10.3 | novel protein | - | - |  |
| *31* | 17541 | 17765 | + | 8363 | 8.4 | conserved hypothetical protein | YP_008239624 hypothetical protein SP031_00135 [Salmonella phage FSL SP-031] | 6.78e-04 |  |
| *32* | 17765 | 17905 | + | 4983 | 6.5 | novel protein | - | - |  |
| *33* | 18139 | 18363 | + | 8671 | 9.8 | structural protein | AGF89298 hypothetical protein SP062_00090 [Salmonella phage FSL SP-062] | 3.43e-06 | pfam05206 Methyltransferase TRM13 |
| *34* | 18360 | 18674 | + | 11611 | 5.7 | NinX protein | ABQ88415 hypothetical protein ECRS218_0035 [Enterobacteria phage CUS-3] | 7.49e-16 | pfam10765 DUF2591 No known function |
| *35* | 18667 | 18846 | + | 6581 | 10.4 | novel protein | - | - |  |
| *36* | 18850 | 19131 | + | 10338 | 9.4 | novel protein | - | - |  |
| *37* | 19128 | 19343 | + | 7552 | 6 | novel protein | - | - |  |
| *38* | 19353 | 19538 | + | 6890 | 8 | novel protein | - | - |  |
| *39* | 19618 | 20073 | + | 16583 | 8.9 | terminase, small subunit | YP_224139 gp1 [Enterobacteria phage ES18] | 8.17e-07 | pfam03592 Terminase small subunit |
| *40* | 20066 | 21361 | + | 48475 | 6.2 | terminase, large subunit | ADA82397.1 putative terminase [Escherichia phage K1ind2] | 7.50e-170 | pfam03237 Terminase-like family |
| *41* | 21376 | 21729 | + | 12696 | 4.6 | novel protein | - | - |  |
| *42* | 21726 | 23228 | + | 55076 | 4.7 | portal protein | YP_008239713 portal protein [Salmonella phage Jersey] | 0 | pfam05133 SPP1 Gp6-like Portal protein |
| *43* | 23225 | 24292 | + | 39255 | 5.7 | head morphogenesis protein | ADA82298 putative head protein, SPP1 gp7 family [Escherichia phage K1-dep(1)] | 2.01e-166 | pfam04233 Phage Mu protein F like protein |
| *44* | 24303 | 24773 | + | 16603 | 4.4 | conserved hypothetical protein | YP_008239632 hypothetical protein SP031_00175 [Salmonella phage FSL SP-031] | 1.55e-54 |  |
| T3 | 24785 | 24813 | + | - | - | rho-independent terminator 3 | AGTCGCATGGATGCGGCTATATTCATTTT | | |
| *45* | 24824 | 25156 | - | 12602 | 9.1 | novel protein | - | - |  |
| *P2* | 25387 | 25316 | - | - | - | host promoter 2 | TTGAACTTGTGAGTGCTGTTGTATTCTAAT | | |
| *P3* | 25393 | 25422 | + | - | - | host promoter 3 | TTGCAACGTTATAACATTTTGAGCAATAAT | | |
| *46* | 25527 | 25850 | + | 11298 | 9.5 | holin | NP_720319 holin protein [Salmonella phage ST64T] | 4.83e-47 |  |
| *46b* | 25533 | 25850 | + | 16672 | 9.2 | 46B | - | - | Alternative start codon |
| *47* | 25834 | 26286 | + | 17229 | 9.3 | lysin | ADF97548 PlyM23 [uncultured phage] | 2.35e-58 | cd00737 endolysin_autolysin |
| *48* | 26280 | 26741 | + | 14650 | 5.6 | lysis protein Rz | YP_007151733 lysis protein Rz [Enterobacteria phage HK106] | 4.75e-39 | PHA00276 phage lambda Rz-like lysis protein |
| *49* | 26713 | 27105 | + | 36356 | 4.7 | conserved hypothetical protein | -YP_008239601 hypothetical protein SP031_00020 [Salmonella phage FSL SP-031] | 2.14e-06 |  |
| *50* | 27260 | 27943 | + | 9930 | 6.1 | conserved hypothetical protein | ADA82302 hypothetical protein [Escherichia phage K1-dep(1)] | 5.20e-68 |  |
| *51* | 27953 | 28969 | + | 3966 | 11.6 | major capsid protein | AGX84684 major capsid protein [Salmonella phage SETP7] | 4.59e-67 |  |
| *52* | 29011 | 29292 | + | 17652 | 4.5 | novel protein | - | - |  |
| *53* | 29264 | 29368 | + | 21155 | 8 | novel protein | - | - |  |
| *54* | 29372 | 29884 | + | 12914 | 8 | conserved hypothetical protein | YP_008239642 hypothetical protein SP031_00225 [Salmonella phage FSL SP-031] | 4.15e-84 |  |
| *55* | 29887 | 30501 | + | 19059 | 9.4 | conserved hypothetical protein | AGF88932 hypothetical protein SP049_00055 [Salmonella phage FSL SP-049] | 1.45e-119 |  |
| *56* | 30501 | 30860 | + | 15074 | 4.8 | conserved hypotheticalprotein | YP_008239644 hypothetical protein SP031_00235 [Salmonella phage FSL SP-031] | 1.15e-59 |  |
| *57* | 30857 | 31381 | + | 40443 | 4.3 | conserved hypothetical protein | AFO12418 hypothetical protein [Salmonella phage vB_SenS_AG11] | 2.09e-51 |  |
| *58* | 31381 | 31794 | + | 11715 | 5.8 | conserved hypothetical protein | ADA82260 hypothetical protein [Escherichia phage K1-dep(4)] | 3.48e-81 |  |
| *59* | 31797 | 32963 | + | 15521 | 4.9 | putative tail protein | YP_001110846 putative tail protein [Salmonella phage SETP3] | 0 |  |
| T4 | 32962 | 32986 | - | - | - | rho-independent terminator 4 | CAAAGGCCCTTTTCGGGGCCTTTTATTTT | | |
| T5 | 32971 | 32996 | + | - | - | rho-independent terminator 5 | GGCCCCGAAAAGGGCCTTTGTTATTT | | |
| *60* | 32991 | 33326 | - | 13858 | 5.4 | novel protein | - | - |  |
| P4 | 33328 | 33257 | - | - | - | host promoter 4 | TTGCAATCTGTATTGGCAATGCTATTAT | | |
| P5 | 33353 | 33380 | + | - | - | host promoter 5 | TTACTACTCCTTTGTTTGTGAGATGATAAT | | |
| R4 | 33375 | 33391 | - | - | - | direct repeat 4 | AAACAAATTAGTTGCAA | | |
| *61* | 33434 | 33850 | + | 78175 | 5.7 | conserved hypothetical protein | ADA82315 hypothetical protein [Escherichia phage K1-dep(1)] | 1.13e-63 | pfam08748 DUF1789 No known function |
| *62* | 33856 | 34212 | + | 18249 | 5 | conserved hypothetical protein | C-terminus of ADA82316 hypothetical protein [Escherichia phage K1-dep(1)] | 2.43e-45 | pfam06791 Prophage tail length tape measure protein |
| *63* | 34205 | 36439 | + | 8050 | 7.8 | tape measure protein | YP_001110853 tail length tape measure-related protein [Salmonella phage SETP3] | 0 | pfam08875 DUF1833 No known function |
| *64* | 36442 | 36936 | + | 18699 | 4.5 | conserved hypothetical protein | ADA82269 hypothetical protein [Escherichia phage K1-dep(4)] | 2.81e-71 |  |
| *65* | 36933 | 37127 | + | 13986 | 6.4 | novel protein | - | - |  |
| *66* | 37124 | 37636 | + | 93021 | 5.5 | conserved hypothetical protein | AFO12377 hypothetical protein [Salmonella phage wksl3] | 4.70e-85 |  |
| *67* | 37633 | 37998 | + | 75415 | 5.8 | conserved hypothetical protein | YP_008239654 hypothetical protein SP031_00285 [Salmonella phage FSL SP-031] | 1.40e-57 |  |
| *68* | 37989 | 40535 | + | 93020 | 6 | putative tail fibre protein | YP_008058251 hypothetical protein [Salmonella phage L13] | 0 |  |
| *69* | 40550 | 42723 | + | 75415 | 6 | tailspike protein | YP_007349013 putative polygalacturonase [Serratia phage phiMAM1] | 1.48e-120 | - |

BLASTP searches conducted against NCBI Viruses (taxid:10239) database November 11, 2013
