# Supplementary material for: The genome and proteome of Serratia bacteriophage η which forms unstable lysogens
Source: Virol J. 2014 Jan 16;11:6. doi: 10.1186/1743-422X-11-6 (PMC3918226; doi:10.1186/1743-422X-11-6)
Supplement: Additional file 4: Table S2 — An SDS-PAGE analysis of the purified structural phage proteins on a 12% SDS-PAGE separation gel alongside a PageRuler™ prestained protein ladder (Fermentas) is presented. Visible protein bands were excised from the gel, and labeled as shown in the figure. Subsequently the nature of the protein in the visible band was identified by UPLC-LTQFT -MS/MS analysis. For every detected protein the protein name, the predicted molecular size (Da), the maximum number of unique spectra and sequence coverage (%) is listed. [file 1743-422X-11-6-S4.doc]

**Additional File 4, Table S2.** SDS-PAGE and mass spectrometric analysis of structural proteins in *Serratia marcescens* phage η.

**A. SDS-PAGE**


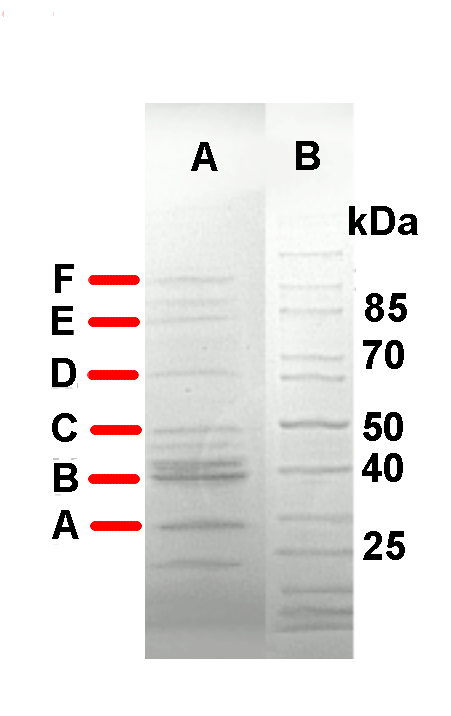


**B. Protein identification of the gel bands by LC MS/MS analyses**

| **Gel band** | **Gene annotation**  **NCBI access #** | **Protein name** | **MW** | **Protein sequence**  **coverage** | **Score** |
| --- | --- | --- | --- | --- | --- |
| A (38 kDa) | [gene_51|](http://10.139.25.109/mascot/cgi/protein_view.pl?file=../data/20100316/F010064.dat&hit=gene_51|putative&px=1&ave_thresh=1&_sigthreshold=0.05&_server_mudpit_switch=1e-009&_ignoreionsscorebelow=20)YP_008130344 | capsid protein | 36332 | 72% | 9500 |
| B (40 kDa) | [gene_59|](http://10.139.25.109/mascot/cgi/protein_view.pl?file=../data/20100316/F010065.dat&hit=gene_59|putative&px=1&ave_thresh=2&_sigthreshold=0.05&_server_mudpit_switch=1e-009&_ignoreionsscorebelow=20)YP_008130352 | tail protein | 40531 | 53% | 2376 |
|  | [gene_43|](http://10.139.25.109/mascot/cgi/protein_view.pl?file=../data/20100316/F010065.dat&hit=gene_43|putative&px=1&ave_thresh=2&_sigthreshold=0.05&_server_mudpit_switch=1e-009&_ignoreionsscorebelow=20)YP_008130336 | head morphogenesis protein | 39344 | 42% | 732 |
| C (55 kDa) | [gene_42|](http://10.139.25.109/mascot/cgi/protein_view.pl?file=../data/20100316/F010066.dat&hit=gene_42|conserved&px=1&ave_thresh=1&_sigthreshold=0.05&_server_mudpit_switch=1e-009&_ignoreionsscorebelow=20)YP_008130335 | portal protein | 55097 | 68% | 3901 |
| D (70 kDa) | [gene_69|](http://10.139.25.109/mascot/cgi/protein_view.pl?file=../data/20100316/F010067.dat&hit=gene_69|conserved&px=1&ave_thresh=1&_sigthreshold=0.05&_server_mudpit_switch=1e-009&_ignoreionsscorebelow=20)YP_008130362 | tail spike protein | 76109 | 49% | 4151 |
| E (75 kDa) | [gene_63|](http://10.139.25.109/mascot/cgi/master_results.pl?file=..%2Fdata%2F20100316%2FF010068.dat&REPTYPE=select&_sigthreshold=0.05&REPORT=AUTO&_server_mudpit_switch=0.000000001&_ignoreionsscorebelow=20&_showsubsets=0&_showpopups=TRUE&_sortunassigned=scoredown&rbrchkbox=on&_requireboldred=1#Hit1%23Hit1)YP_008130356 | tail tape measure protein | 78127 | 60% | 4167 |
| F (85 kDa) | [gene_68|](http://10.139.25.109/mascot/cgi/master_results.pl?file=..%2Fdata%2F20100316%2FF010069.dat&REPTYPE=select&_sigthreshold=0.05&REPORT=AUTO&_server_mudpit_switch=0.000000001&_ignoreionsscorebelow=20&_showsubsets=0&_showpopups=TRUE&_sortunassigned=scoredown&rbrchkbox=on&_requireboldred=1#Hit4%23Hit4)YP_008130361 | putative tail protein | 93247 | 39% | 1789 |

**1.** [gene_51|putative](http://10.139.25.109/mascot/cgi/protein_view.pl?file=../data/20100316/F010064.dat&hit=gene_51|putative&px=1&ave_thresh=1&_sigthreshold=0.05&_server_mudpit_switch=1e-009&_ignoreionsscorebelow=20) capsid protein|[Serratia phage Eta] **Mass:** 36332    **Score:** 9500   **Queries matched:** 214(214),   **emPAI:** 3683.86; Sequence Coverage: **72%**; Matched peptides shown in **Bold Red**

**1** **MATVQLADIY NPLVFDAAVQ EKQVELNR**FI QSGVAVVDPQ LTAMASVGGN IGELPFYKPL GTEEPNYSTD NPATLSTPAK ITSSK**MIYRL AAQNKSWSTM**

**101 DLARELALED PMGAITGRIG QYWATNNEKR IIQSVRGLIA DNVANDGGDM LFDISAATDT AVTDANRADA DAIIDTVQTM GDHGEMLSAI AMHSVVYR**RL

**201** QK**QNLIDFIP DARGEVNIPM YQGK**TVIVDD SLAGVTYGTT PANVYYYTIL FGAGEFR**LGE GQPTTPSAID RVEAAGNGGG QDIIYSRRSD IIHPLGFQFT**

**301 SASVAGTSAT QAELATAANW NRVYERKNVS LAVLKSNG**

**Start - End Observed Mr(expt) Mr(calc) ppm Miss Sequence**

**1 - 22 812.7548 2435.2425 2435.2406 1 0 -.MATVQLADIYNPLVFDAAVQEK.Q**  ([Ions score 94](http://gary/mascot/cgi/peptide_view.pl?file=../data/20130722/F001265.dat&query=837&hit=1&index=gene_51|putative&px=1&section=5&ave_thresh=3&_ignoreionsscorebelow=20&report=0&_sigthreshold=0.05&_msresflags=1089&_msresflags2=2&percolate=-1&percolate_rt=0))

**2 - 22 1153.1008 2304.1871 2304.2002 -6 0 M.ATVQLADIYNPLVFDAAVQEK.Q**  ([Ions score 118](http://gary/mascot/cgi/peptide_view.pl?file=../data/20130722/F001265.dat&query=798&hit=1&index=gene_51|putative&px=1&section=5&ave_thresh=3&_ignoreionsscorebelow=20&report=0&_sigthreshold=0.05&_msresflags=1089&_msresflags2=2&percolate=-1&percolate_rt=0))

**2 - 22 769.0720 2304.1942 2304.2002 -3 0 M.ATVQLADIYNPLVFDAAVQEK.Q**  ([Ions score 80](http://gary/mascot/cgi/peptide_view.pl?file=../data/20130722/F001265.dat&query=799&hit=1&index=gene_51|putative&px=1&section=5&ave_thresh=3&_ignoreionsscorebelow=20&report=0&_sigthreshold=0.05&_msresflags=1089&_msresflags2=2&percolate=-1&percolate_rt=0))

**2 - 22 1153.1049 2304.1952 2304.2002 -2 0 M.ATVQLADIYNPLVFDAAVQEK.Q**  ([Ions score 117](http://gary/mascot/cgi/peptide_view.pl?file=../data/20130722/F001265.dat&query=800&hit=1&index=gene_51|putative&px=1&section=5&ave_thresh=3&_ignoreionsscorebelow=20&report=0&_sigthreshold=0.05&_msresflags=1089&_msresflags2=2&percolate=-1&percolate_rt=0))

**2 - 22 769.0725 2304.1955 2304.2002 -2 0 M.ATVQLADIYNPLVFDAAVQEK.Q**  ([Ions score 113](http://gary/mascot/cgi/peptide_view.pl?file=../data/20130722/F001265.dat&query=801&hit=1&index=gene_51|putative&px=1&section=5&ave_thresh=3&_ignoreionsscorebelow=20&report=0&_sigthreshold=0.05&_msresflags=1089&_msresflags2=2&percolate=-1&percolate_rt=0))

**2 - 22 769.0726 2304.1961 2304.2002 -2 0 M.ATVQLADIYNPLVFDAAVQEK.Q**  ([Ions score 80](http://gary/mascot/cgi/peptide_view.pl?file=../data/20130722/F001265.dat&query=802&hit=1&index=gene_51|putative&px=1&section=5&ave_thresh=3&_ignoreionsscorebelow=20&report=0&_sigthreshold=0.05&_msresflags=1089&_msresflags2=2&percolate=-1&percolate_rt=0))

**2 - 22 769.0728 2304.1964 2304.2002 -2 0 M.ATVQLADIYNPLVFDAAVQEK.Q**  ([Ions score 91](http://gary/mascot/cgi/peptide_view.pl?file=../data/20130722/F001265.dat&query=803&hit=1&index=gene_51|putative&px=1&section=5&ave_thresh=3&_ignoreionsscorebelow=20&report=0&_sigthreshold=0.05&_msresflags=1089&_msresflags2=2&percolate=-1&percolate_rt=0))

**2 - 22 769.0732 2304.1977 2304.2002 -1 0 M.ATVQLADIYNPLVFDAAVQEK.Q**  ([Ions score 87](http://gary/mascot/cgi/peptide_view.pl?file=../data/20130722/F001265.dat&query=804&hit=1&index=gene_51|putative&px=1&section=5&ave_thresh=3&_ignoreionsscorebelow=20&report=0&_sigthreshold=0.05&_msresflags=1089&_msresflags2=2&percolate=-1&percolate_rt=0))

**2 - 22 769.0736 2304.1990 2304.2002 0 0 M.ATVQLADIYNPLVFDAAVQEK.Q**  ([Ions score 85](http://gary/mascot/cgi/peptide_view.pl?file=../data/20130722/F001265.dat&query=805&hit=1&index=gene_51|putative&px=1&section=5&ave_thresh=3&_ignoreionsscorebelow=20&report=0&_sigthreshold=0.05&_msresflags=1089&_msresflags2=2&percolate=-1&percolate_rt=0))

**2 - 22 769.0736 2304.1990 2304.2002 0 0 M.ATVQLADIYNPLVFDAAVQEK.Q**  ([Ions score 102](http://gary/mascot/cgi/peptide_view.pl?file=../data/20130722/F001265.dat&query=806&hit=1&index=gene_51|putative&px=1&section=5&ave_thresh=3&_ignoreionsscorebelow=20&report=0&_sigthreshold=0.05&_msresflags=1089&_msresflags2=2&percolate=-1&percolate_rt=0))

**2 - 22 769.0737 2304.1992 2304.2002 0 0 M.ATVQLADIYNPLVFDAAVQEK.Q**  ([Ions score 86](http://gary/mascot/cgi/peptide_view.pl?file=../data/20130722/F001265.dat&query=807&hit=1&index=gene_51|putative&px=1&section=5&ave_thresh=3&_ignoreionsscorebelow=20&report=0&_sigthreshold=0.05&_msresflags=1089&_msresflags2=2&percolate=-1&percolate_rt=0))

**2 - 22 769.0737 2304.1992 2304.2002 0 0 M.ATVQLADIYNPLVFDAAVQEK.Q**  ([Ions score 85](http://gary/mascot/cgi/peptide_view.pl?file=../data/20130722/F001265.dat&query=808&hit=1&index=gene_51|putative&px=1&section=5&ave_thresh=3&_ignoreionsscorebelow=20&report=0&_sigthreshold=0.05&_msresflags=1089&_msresflags2=2&percolate=-1&percolate_rt=0))

**2 - 22 769.0738 2304.1995 2304.2002 0 0 M.ATVQLADIYNPLVFDAAVQEK.Q**  ([Ions score 77](http://gary/mascot/cgi/peptide_view.pl?file=../data/20130722/F001265.dat&query=809&hit=1&index=gene_51|putative&px=1&section=5&ave_thresh=3&_ignoreionsscorebelow=20&report=0&_sigthreshold=0.05&_msresflags=1089&_msresflags2=2&percolate=-1&percolate_rt=0))

**2 - 22 1153.1071 2304.1996 2304.2002 0 0 M.ATVQLADIYNPLVFDAAVQEK.Q**  ([Ions score 119](http://gary/mascot/cgi/peptide_view.pl?file=../data/20130722/F001265.dat&query=810&hit=1&index=gene_51|putative&px=1&section=5&ave_thresh=3&_ignoreionsscorebelow=20&report=0&_sigthreshold=0.05&_msresflags=1089&_msresflags2=2&percolate=-1&percolate_rt=0))

**2 - 22 1153.1072 2304.1998 2304.2002 0 0 M.ATVQLADIYNPLVFDAAVQEK.Q**  ([Ions score 93](http://gary/mascot/cgi/peptide_view.pl?file=../data/20130722/F001265.dat&query=811&hit=1&index=gene_51|putative&px=1&section=5&ave_thresh=3&_ignoreionsscorebelow=20&report=0&_sigthreshold=0.05&_msresflags=1089&_msresflags2=2&percolate=-1&percolate_rt=0))

**2 - 22 769.0742 2304.2007 2304.2002 0 0 M.ATVQLADIYNPLVFDAAVQEK.Q**  ([Ions score 93](http://gary/mascot/cgi/peptide_view.pl?file=../data/20130722/F001265.dat&query=812&hit=1&index=gene_51|putative&px=1&section=5&ave_thresh=3&_ignoreionsscorebelow=20&report=0&_sigthreshold=0.05&_msresflags=1089&_msresflags2=2&percolate=-1&percolate_rt=0))

**2 - 22 769.0743 2304.2010 2304.2002 0 0 M.ATVQLADIYNPLVFDAAVQEK.Q**  ([Ions score 96](http://gary/mascot/cgi/peptide_view.pl?file=../data/20130722/F001265.dat&query=813&hit=1&index=gene_51|putative&px=1&section=5&ave_thresh=3&_ignoreionsscorebelow=20&report=0&_sigthreshold=0.05&_msresflags=1089&_msresflags2=2&percolate=-1&percolate_rt=0))

**2 - 22 769.0745 2304.2017 2304.2002 1 0 M.ATVQLADIYNPLVFDAAVQEK.Q**  ([Ions score 68](http://gary/mascot/cgi/peptide_view.pl?file=../data/20130722/F001265.dat&query=814&hit=1&index=gene_51|putative&px=1&section=5&ave_thresh=3&_ignoreionsscorebelow=20&report=0&_sigthreshold=0.05&_msresflags=1089&_msresflags2=2&percolate=-1&percolate_rt=0))

**2 - 22 1153.1083 2304.2020 2304.2002 1 0 M.ATVQLADIYNPLVFDAAVQEK.Q**  ([Ions score 118](http://gary/mascot/cgi/peptide_view.pl?file=../data/20130722/F001265.dat&query=815&hit=1&index=gene_51|putative&px=1&section=5&ave_thresh=3&_ignoreionsscorebelow=20&report=0&_sigthreshold=0.05&_msresflags=1089&_msresflags2=2&percolate=-1&percolate_rt=0))

**2 - 22 769.0753 2304.2041 2304.2002 2 0 M.ATVQLADIYNPLVFDAAVQEK.Q**  ([Ions score 90](http://gary/mascot/cgi/peptide_view.pl?file=../data/20130722/F001265.dat&query=816&hit=1&index=gene_51|putative&px=1&section=5&ave_thresh=3&_ignoreionsscorebelow=20&report=0&_sigthreshold=0.05&_msresflags=1089&_msresflags2=2&percolate=-1&percolate_rt=0))

**2 - 22 1153.1102 2304.2059 2304.2002 2 0 M.ATVQLADIYNPLVFDAAVQEK.Q**  ([Ions score 112](http://gary/mascot/cgi/peptide_view.pl?file=../data/20130722/F001265.dat&query=817&hit=1&index=gene_51|putative&px=1&section=5&ave_thresh=3&_ignoreionsscorebelow=20&report=0&_sigthreshold=0.05&_msresflags=1089&_msresflags2=2&percolate=-1&percolate_rt=0))

**2 - 22 1153.1121 2304.2096 2304.2002 4 0 M.ATVQLADIYNPLVFDAAVQEK.Q**  ([Ions score 112](http://gary/mascot/cgi/peptide_view.pl?file=../data/20130722/F001265.dat&query=818&hit=1&index=gene_51|putative&px=1&section=5&ave_thresh=3&_ignoreionsscorebelow=20&report=0&_sigthreshold=0.05&_msresflags=1089&_msresflags2=2&percolate=-1&percolate_rt=0))

**23 - 28 371.1982 740.3817 740.3817 0 0 K.QVELNR.F**  Gln->pyro-Glu (N-term Q) ([Ions score 43](http://gary/mascot/cgi/peptide_view.pl?file=../data/20130722/F001265.dat&query=27&hit=1&index=gene_51|putative&px=1&section=5&ave_thresh=3&_ignoreionsscorebelow=20&report=0&_sigthreshold=0.05&_msresflags=1089&_msresflags2=2&percolate=-1&percolate_rt=0))

**23 - 28 379.7101 757.4057 757.4082 -3 0 K.QVELNR.F**  ([Ions score 30](http://gary/mascot/cgi/peptide_view.pl?file=../data/20130722/F001265.dat&query=31&hit=1&index=gene_51|putative&px=1&section=5&ave_thresh=3&_ignoreionsscorebelow=20&report=0&_sigthreshold=0.05&_msresflags=1089&_msresflags2=2&percolate=-1&percolate_rt=0))

**23 - 28 379.7109 757.4073 757.4082 -1 0 K.QVELNR.F**  ([Ions score 36](http://gary/mascot/cgi/peptide_view.pl?file=../data/20130722/F001265.dat&query=32&hit=1&index=gene_51|putative&px=1&section=5&ave_thresh=3&_ignoreionsscorebelow=20&report=0&_sigthreshold=0.05&_msresflags=1089&_msresflags2=2&percolate=-1&percolate_rt=0))

**23 - 28 379.7112 757.4079 757.4082 0 0 K.QVELNR.F**  ([Ions score 30](http://gary/mascot/cgi/peptide_view.pl?file=../data/20130722/F001265.dat&query=33&hit=1&index=gene_51|putative&px=1&section=5&ave_thresh=3&_ignoreionsscorebelow=20&report=0&_sigthreshold=0.05&_msresflags=1089&_msresflags2=2&percolate=-1&percolate_rt=0))

**23 - 28 379.7112 757.4079 757.4082 0 0 K.QVELNR.F**  ([Ions score 24](http://gary/mascot/cgi/peptide_view.pl?file=../data/20130722/F001265.dat&query=34&hit=1&index=gene_51|putative&px=1&section=5&ave_thresh=3&_ignoreionsscorebelow=20&report=0&_sigthreshold=0.05&_msresflags=1089&_msresflags2=2&percolate=-1&percolate_rt=0))

**23 - 28 379.7113 757.4080 757.4082 0 0 K.QVELNR.F**  ([Ions score 33](http://gary/mascot/cgi/peptide_view.pl?file=../data/20130722/F001265.dat&query=35&hit=1&index=gene_51|putative&px=1&section=5&ave_thresh=3&_ignoreionsscorebelow=20&report=0&_sigthreshold=0.05&_msresflags=1089&_msresflags2=2&percolate=-1&percolate_rt=0))

**23 - 28 379.7115 757.4085 757.4082 0 0 K.QVELNR.F**  ([Ions score 38](http://gary/mascot/cgi/peptide_view.pl?file=../data/20130722/F001265.dat&query=36&hit=1&index=gene_51|putative&px=1&section=5&ave_thresh=3&_ignoreionsscorebelow=20&report=0&_sigthreshold=0.05&_msresflags=1089&_msresflags2=2&percolate=-1&percolate_rt=0))

**23 - 28 379.7118 757.4090 757.4082 1 0 K.QVELNR.F**  ([Ions score 42](http://gary/mascot/cgi/peptide_view.pl?file=../data/20130722/F001265.dat&query=37&hit=1&index=gene_51|putative&px=1&section=5&ave_thresh=3&_ignoreionsscorebelow=20&report=0&_sigthreshold=0.05&_msresflags=1089&_msresflags2=2&percolate=-1&percolate_rt=0))

**23 - 28 379.7120 757.4095 757.4082 2 0 K.QVELNR.F**  ([Ions score 21](http://gary/mascot/cgi/peptide_view.pl?file=../data/20130722/F001265.dat&query=38&hit=1&index=gene_51|putative&px=1&section=5&ave_thresh=3&_ignoreionsscorebelow=20&report=0&_sigthreshold=0.05&_msresflags=1089&_msresflags2=2&percolate=-1&percolate_rt=0))

**23 - 28 379.7128 757.4110 757.4082 4 0 K.QVELNR.F**  ([Ions score 28](http://gary/mascot/cgi/peptide_view.pl?file=../data/20130722/F001265.dat&query=39&hit=1&index=gene_51|putative&px=1&section=5&ave_thresh=3&_ignoreionsscorebelow=20&report=0&_sigthreshold=0.05&_msresflags=1089&_msresflags2=2&percolate=-1&percolate_rt=0))

**86 - 95 604.3337 1206.6528 1206.6543 -1 1 K.MIYRLAAQNK.S**  ([Ions score 29](http://gary/mascot/cgi/peptide_view.pl?file=../data/20130722/F001265.dat&query=381&hit=1&index=gene_51|putative&px=1&section=5&ave_thresh=3&_ignoreionsscorebelow=20&report=0&_sigthreshold=0.05&_msresflags=1089&_msresflags2=2&percolate=-1&percolate_rt=0))

**86 - 95 604.3337 1206.6529 1206.6543 -1 1 K.MIYRLAAQNK.S**  ([Ions score 37](http://gary/mascot/cgi/peptide_view.pl?file=../data/20130722/F001265.dat&query=382&hit=1&index=gene_51|putative&px=1&section=5&ave_thresh=3&_ignoreionsscorebelow=20&report=0&_sigthreshold=0.05&_msresflags=1089&_msresflags2=2&percolate=-1&percolate_rt=0))

**86 - 95 403.2250 1206.6531 1206.6543 -1 1 K.MIYRLAAQNK.S**  ([Ions score 22](http://gary/mascot/cgi/peptide_view.pl?file=../data/20130722/F001265.dat&query=383&hit=1&index=gene_51|putative&px=1&section=5&ave_thresh=3&_ignoreionsscorebelow=20&report=0&_sigthreshold=0.05&_msresflags=1089&_msresflags2=2&percolate=-1&percolate_rt=0))

**86 - 95 604.3340 1206.6534 1206.6543 -1 1 K.MIYRLAAQNK.S**  ([Ions score 46](http://gary/mascot/cgi/peptide_view.pl?file=../data/20130722/F001265.dat&query=385&hit=1&index=gene_51|putative&px=1&section=5&ave_thresh=3&_ignoreionsscorebelow=20&report=0&_sigthreshold=0.05&_msresflags=1089&_msresflags2=2&percolate=-1&percolate_rt=0))

**86 - 95 403.2253 1206.6542 1206.6543 0 1 K.MIYRLAAQNK.S**  ([Ions score 26](http://gary/mascot/cgi/peptide_view.pl?file=../data/20130722/F001265.dat&query=386&hit=1&index=gene_51|putative&px=1&section=5&ave_thresh=3&_ignoreionsscorebelow=20&report=0&_sigthreshold=0.05&_msresflags=1089&_msresflags2=2&percolate=-1&percolate_rt=0))

**86 - 95 403.2255 1206.6547 1206.6543 0 1 K.MIYRLAAQNK.S**  ([Ions score 24](http://gary/mascot/cgi/peptide_view.pl?file=../data/20130722/F001265.dat&query=388&hit=1&index=gene_51|putative&px=1&section=5&ave_thresh=3&_ignoreionsscorebelow=20&report=0&_sigthreshold=0.05&_msresflags=1089&_msresflags2=2&percolate=-1&percolate_rt=0))

**86 - 95 604.3351 1206.6556 1206.6543 1 1 K.MIYRLAAQNK.S**  ([Ions score 40](http://gary/mascot/cgi/peptide_view.pl?file=../data/20130722/F001265.dat&query=392&hit=1&index=gene_51|putative&px=1&section=5&ave_thresh=3&_ignoreionsscorebelow=20&report=0&_sigthreshold=0.05&_msresflags=1089&_msresflags2=2&percolate=-1&percolate_rt=0))

**86 - 95 403.2259 1206.6558 1206.6543 1 1 K.MIYRLAAQNK.S**  ([Ions score 21](http://gary/mascot/cgi/peptide_view.pl?file=../data/20130722/F001265.dat&query=393&hit=1&index=gene_51|putative&px=1&section=5&ave_thresh=3&_ignoreionsscorebelow=20&report=0&_sigthreshold=0.05&_msresflags=1089&_msresflags2=2&percolate=-1&percolate_rt=0))

**86 - 95 403.2263 1206.6570 1206.6543 2 1 K.MIYRLAAQNK.S**  ([Ions score 24](http://gary/mascot/cgi/peptide_view.pl?file=../data/20130722/F001265.dat&query=395&hit=1&index=gene_51|putative&px=1&section=5&ave_thresh=3&_ignoreionsscorebelow=20&report=0&_sigthreshold=0.05&_msresflags=1089&_msresflags2=2&percolate=-1&percolate_rt=0))

**86 - 95 604.3364 1206.6582 1206.6543 3 1 K.MIYRLAAQNK.S**  ([Ions score 27](http://gary/mascot/cgi/peptide_view.pl?file=../data/20130722/F001265.dat&query=397&hit=1&index=gene_51|putative&px=1&section=5&ave_thresh=3&_ignoreionsscorebelow=20&report=0&_sigthreshold=0.05&_msresflags=1089&_msresflags2=2&percolate=-1&percolate_rt=0))

**86 - 95 612.3309 1222.6473 1222.6492 -2 1 K.MIYRLAAQNK.S**  Oxidation (M) ([Ions score 43](http://gary/mascot/cgi/peptide_view.pl?file=../data/20130722/F001265.dat&query=407&hit=1&index=gene_51|putative&px=1&section=5&ave_thresh=3&_ignoreionsscorebelow=20&report=0&_sigthreshold=0.05&_msresflags=1089&_msresflags2=2&percolate=-1&percolate_rt=0))

**90 - 95 322.6896 643.3647 643.3653 -1 0 R.LAAQNK.S**  ([Ions score 30](http://gary/mascot/cgi/peptide_view.pl?file=../data/20130722/F001265.dat&query=4&hit=1&index=gene_51|putative&px=1&section=5&ave_thresh=3&_ignoreionsscorebelow=20&report=0&_sigthreshold=0.05&_msresflags=1089&_msresflags2=2&percolate=-1&percolate_rt=0))

**96 - 104 533.7505 1065.4864 1065.4913 -5 0 K.SWSTMDLAR.E**  ([Ions score 49](http://gary/mascot/cgi/peptide_view.pl?file=../data/20130722/F001265.dat&query=230&hit=1&index=gene_51|putative&px=1&section=5&ave_thresh=3&_ignoreionsscorebelow=20&report=0&_sigthreshold=0.05&_msresflags=1089&_msresflags2=2&percolate=-1&percolate_rt=0))

**96 - 104 533.7509 1065.4873 1065.4913 -4 0 K.SWSTMDLAR.E**  ([Ions score 50](http://gary/mascot/cgi/peptide_view.pl?file=../data/20130722/F001265.dat&query=231&hit=1&index=gene_51|putative&px=1&section=5&ave_thresh=3&_ignoreionsscorebelow=20&report=0&_sigthreshold=0.05&_msresflags=1089&_msresflags2=2&percolate=-1&percolate_rt=0))

**96 - 104 533.7519 1065.4893 1065.4913 -2 0 K.SWSTMDLAR.E**  ([Ions score 32](http://gary/mascot/cgi/peptide_view.pl?file=../data/20130722/F001265.dat&query=232&hit=1&index=gene_51|putative&px=1&section=5&ave_thresh=3&_ignoreionsscorebelow=20&report=0&_sigthreshold=0.05&_msresflags=1089&_msresflags2=2&percolate=-1&percolate_rt=0))

**96 - 104 533.7524 1065.4902 1065.4913 -1 0 K.SWSTMDLAR.E**  ([Ions score 49](http://gary/mascot/cgi/peptide_view.pl?file=../data/20130722/F001265.dat&query=233&hit=1&index=gene_51|putative&px=1&section=5&ave_thresh=3&_ignoreionsscorebelow=20&report=0&_sigthreshold=0.05&_msresflags=1089&_msresflags2=2&percolate=-1&percolate_rt=0))

**96 - 104 533.7525 1065.4904 1065.4913 -1 0 K.SWSTMDLAR.E**  ([Ions score 42](http://gary/mascot/cgi/peptide_view.pl?file=../data/20130722/F001265.dat&query=234&hit=1&index=gene_51|putative&px=1&section=5&ave_thresh=3&_ignoreionsscorebelow=20&report=0&_sigthreshold=0.05&_msresflags=1089&_msresflags2=2&percolate=-1&percolate_rt=0))

**96 - 104 533.7534 1065.4923 1065.4913 1 0 K.SWSTMDLAR.E**  ([Ions score 33](http://gary/mascot/cgi/peptide_view.pl?file=../data/20130722/F001265.dat&query=235&hit=1&index=gene_51|putative&px=1&section=5&ave_thresh=3&_ignoreionsscorebelow=20&report=0&_sigthreshold=0.05&_msresflags=1089&_msresflags2=2&percolate=-1&percolate_rt=0))

**96 - 104 533.7546 1065.4947 1065.4913 3 0 K.SWSTMDLAR.E**  ([Ions score 38](http://gary/mascot/cgi/peptide_view.pl?file=../data/20130722/F001265.dat&query=236&hit=1&index=gene_51|putative&px=1&section=5&ave_thresh=3&_ignoreionsscorebelow=20&report=0&_sigthreshold=0.05&_msresflags=1089&_msresflags2=2&percolate=-1&percolate_rt=0))

**96 - 104 541.7477 1081.4809 1081.4862 -5 0 K.SWSTMDLAR.E**  Oxidation (M) ([Ions score 36](http://gary/mascot/cgi/peptide_view.pl?file=../data/20130722/F001265.dat&query=247&hit=1&index=gene_51|putative&px=1&section=5&ave_thresh=3&_ignoreionsscorebelow=20&report=0&_sigthreshold=0.05&_msresflags=1089&_msresflags2=2&percolate=-1&percolate_rt=0))

**96 - 104 541.7491 1081.4837 1081.4862 -2 0 K.SWSTMDLAR.E**  Oxidation (M) ([Ions score 26](http://gary/mascot/cgi/peptide_view.pl?file=../data/20130722/F001265.dat&query=248&hit=1&index=gene_51|putative&px=1&section=5&ave_thresh=3&_ignoreionsscorebelow=20&report=0&_sigthreshold=0.05&_msresflags=1089&_msresflags2=2&percolate=-1&percolate_rt=0))

**96 - 118 630.8093 2519.2080 2519.2148 -3 1 K.SWSTMDLARELALEDPMGAITGR.I**  ([Ions score 79](http://gary/mascot/cgi/peptide_view.pl?file=../data/20130722/F001265.dat&query=870&hit=1&index=gene_51|putative&px=1&section=5&ave_thresh=3&_ignoreionsscorebelow=20&report=0&_sigthreshold=0.05&_msresflags=1089&_msresflags2=2&percolate=-1&percolate_rt=0))

**96 - 118 1260.6119 2519.2093 2519.2148 -2 1 K.SWSTMDLARELALEDPMGAITGR.I**  ([Ions score 49](http://gary/mascot/cgi/peptide_view.pl?file=../data/20130722/F001265.dat&query=871&hit=1&index=gene_51|putative&px=1&section=5&ave_thresh=3&_ignoreionsscorebelow=20&report=0&_sigthreshold=0.05&_msresflags=1089&_msresflags2=2&percolate=-1&percolate_rt=0))

**96 - 118 840.7441 2519.2104 2519.2148 -2 1 K.SWSTMDLARELALEDPMGAITGR.I**  ([Ions score 72](http://gary/mascot/cgi/peptide_view.pl?file=../data/20130722/F001265.dat&query=872&hit=1&index=gene_51|putative&px=1&section=5&ave_thresh=3&_ignoreionsscorebelow=20&report=0&_sigthreshold=0.05&_msresflags=1089&_msresflags2=2&percolate=-1&percolate_rt=0))

**96 - 118 846.0733 2535.1981 2535.2097 -5 1 K.SWSTMDLARELALEDPMGAITGR.I**  Oxidation (M) ([Ions score 27](http://gary/mascot/cgi/peptide_view.pl?file=../data/20130722/F001265.dat&query=880&hit=1&index=gene_51|putative&px=1&section=5&ave_thresh=3&_ignoreionsscorebelow=20&report=0&_sigthreshold=0.05&_msresflags=1089&_msresflags2=2&percolate=-1&percolate_rt=0))

**96 - 118 1268.6080 2535.2015 2535.2097 -3 1 K.SWSTMDLARELALEDPMGAITGR.I**  Oxidation (M) ([Ions score 46](http://gary/mascot/cgi/peptide_view.pl?file=../data/20130722/F001265.dat&query=881&hit=1&index=gene_51|putative&px=1&section=5&ave_thresh=3&_ignoreionsscorebelow=20&report=0&_sigthreshold=0.05&_msresflags=1089&_msresflags2=2&percolate=-1&percolate_rt=0))

**96 - 118 846.0753 2535.2040 2535.2097 -2 1 K.SWSTMDLARELALEDPMGAITGR.I**  Oxidation (M) ([Ions score 47](http://gary/mascot/cgi/peptide_view.pl?file=../data/20130722/F001265.dat&query=882&hit=1&index=gene_51|putative&px=1&section=5&ave_thresh=3&_ignoreionsscorebelow=20&report=0&_sigthreshold=0.05&_msresflags=1089&_msresflags2=2&percolate=-1&percolate_rt=0))

**96 - 118 1268.6123 2535.2100 2535.2097 0 1 K.SWSTMDLARELALEDPMGAITGR.I**  Oxidation (M) ([Ions score 43](http://gary/mascot/cgi/peptide_view.pl?file=../data/20130722/F001265.dat&query=883&hit=1&index=gene_51|putative&px=1&section=5&ave_thresh=3&_ignoreionsscorebelow=20&report=0&_sigthreshold=0.05&_msresflags=1089&_msresflags2=2&percolate=-1&percolate_rt=0))

**96 - 118 634.8104 2535.2126 2535.2097 1 1 K.SWSTMDLARELALEDPMGAITGR.I**  Oxidation (M) ([Ions score 73](http://gary/mascot/cgi/peptide_view.pl?file=../data/20130722/F001265.dat&query=884&hit=1&index=gene_51|putative&px=1&section=5&ave_thresh=3&_ignoreionsscorebelow=20&report=0&_sigthreshold=0.05&_msresflags=1089&_msresflags2=2&percolate=-1&percolate_rt=0))

**96 - 118 634.8123 2535.2199 2535.2097 4 1 K.SWSTMDLARELALEDPMGAITGR.I**  Oxidation (M) ([Ions score 77](http://gary/mascot/cgi/peptide_view.pl?file=../data/20130722/F001265.dat&query=886&hit=1&index=gene_51|putative&px=1&section=5&ave_thresh=3&_ignoreionsscorebelow=20&report=0&_sigthreshold=0.05&_msresflags=1089&_msresflags2=2&percolate=-1&percolate_rt=0))

**96 - 118 851.4036 2551.1891 2551.2047 -6 1 K.SWSTMDLARELALEDPMGAITGR.I** 2 Oxidation (M) ([Ions score 40](http://gary/mascot/cgi/peptide_view.pl?file=../data/20130722/F001265.dat&query=893&hit=1&index=gene_51|putative&px=1&section=5&ave_thresh=3&_ignoreionsscorebelow=20&report=0&_sigthreshold=0.05&_msresflags=1089&_msresflags2=2&percolate=-1&percolate_rt=0))

**96 - 118 851.4063 2551.1971 2551.2047 -3 1 K.SWSTMDLARELALEDPMGAITGR.I** 2 Oxidation (M) ([Ions score 26](http://gary/mascot/cgi/peptide_view.pl?file=../data/20130722/F001265.dat&query=894&hit=1&index=gene_51|putative&px=1&section=5&ave_thresh=3&_ignoreionsscorebelow=20&report=0&_sigthreshold=0.05&_msresflags=1089&_msresflags2=2&percolate=-1&percolate_rt=0))

**96 - 118 638.8066 2551.1972 2551.2047 -3 1 K.SWSTMDLARELALEDPMGAITGR.I** 2 Oxidation (M) ([Ions score 34](http://gary/mascot/cgi/peptide_view.pl?file=../data/20130722/F001265.dat&query=895&hit=1&index=gene_51|putative&px=1&section=5&ave_thresh=3&_ignoreionsscorebelow=20&report=0&_sigthreshold=0.05&_msresflags=1089&_msresflags2=2&percolate=-1&percolate_rt=0))

**96 - 118 851.4067 2551.1984 2551.2047 -2 1 K.SWSTMDLARELALEDPMGAITGR.I** 2 Oxidation (M) ([Ions score 67](http://gary/mascot/cgi/peptide_view.pl?file=../data/20130722/F001265.dat&query=896&hit=1&index=gene_51|putative&px=1&section=5&ave_thresh=3&_ignoreionsscorebelow=20&report=0&_sigthreshold=0.05&_msresflags=1089&_msresflags2=2&percolate=-1&percolate_rt=0))

**96 - 118 638.8074 2551.2004 2551.2047 -2 1 K.SWSTMDLARELALEDPMGAITGR.I** 2 Oxidation (M) ([Ions score 59](http://gary/mascot/cgi/peptide_view.pl?file=../data/20130722/F001265.dat&query=897&hit=1&index=gene_51|putative&px=1&section=5&ave_thresh=3&_ignoreionsscorebelow=20&report=0&_sigthreshold=0.05&_msresflags=1089&_msresflags2=2&percolate=-1&percolate_rt=0))

**96 - 118 1276.6108 2551.2071 2551.2047 1 1 K.SWSTMDLARELALEDPMGAITGR.I** 2 Oxidation (M) ([Ions score 34](http://gary/mascot/cgi/peptide_view.pl?file=../data/20130722/F001265.dat&query=898&hit=1&index=gene_51|putative&px=1&section=5&ave_thresh=3&_ignoreionsscorebelow=20&report=0&_sigthreshold=0.05&_msresflags=1089&_msresflags2=2&percolate=-1&percolate_rt=0))

**96 - 129 956.9617 3823.8178 3823.8297 -3 2 K.SWSTMDLARELALEDPMGAITGRIGQYWATNNEK.R**  ([Ions score 72](http://gary/mascot/cgi/peptide_view.pl?file=../data/20130722/F001265.dat&query=1028&hit=1&index=gene_51|putative&px=1&section=5&ave_thresh=3&_ignoreionsscorebelow=20&report=0&_sigthreshold=0.05&_msresflags=1089&_msresflags2=2&percolate=-1&percolate_rt=0))

**96 - 129 960.9611 3839.8151 3839.8247 -2 2 K.SWSTMDLARELALEDPMGAITGRIGQYWATNNEK.R**

Oxidation(M)([Ions score 81](http://gary/mascot/cgi/peptide_view.pl?file=../data/20130722/F001265.dat&query=1029&hit=1&index=gene_51|putative&px=1&section=5&ave_thresh=3&_ignoreionsscorebelow=20&report=0&_sigthreshold=0.05&_msresflags=1089&_msresflags2=2&percolate=-1&percolate_rt=0))

**96 - 129 960.9627 3839.8215 3839.8247 -1 2 K.SWSTMDLARELALEDPMGAITGRIGQYWATNNEK.R**

Oxidation (M) ([Ions score 82](http://gary/mascot/cgi/peptide_view.pl?file=../data/20130722/F001265.dat&query=1030&hit=1&index=gene_51|putative&px=1&section=5&ave_thresh=3&_ignoreionsscorebelow=20&report=0&_sigthreshold=0.05&_msresflags=1089&_msresflags2=2&percolate=-1&percolate_rt=0))

**105 - 118 491.5848 1471.7326 1471.7341 -1 0 R.ELALEDPMGAITGR.I**  ([Ions score 66](http://gary/mascot/cgi/peptide_view.pl?file=../data/20130722/F001265.dat&query=634&hit=1&index=gene_51|putative&px=1&section=5&ave_thresh=3&_ignoreionsscorebelow=20&report=0&_sigthreshold=0.05&_msresflags=1089&_msresflags2=2&percolate=-1&percolate_rt=0))

**105 - 118 736.8737 1471.7328 1471.7341 -1 0 R.ELALEDPMGAITGR.I**  ([Ions score 90](http://gary/mascot/cgi/peptide_view.pl?file=../data/20130722/F001265.dat&query=635&hit=1&index=gene_51|putative&px=1&section=5&ave_thresh=3&_ignoreionsscorebelow=20&report=0&_sigthreshold=0.05&_msresflags=1089&_msresflags2=2&percolate=-1&percolate_rt=0))

**105 - 118 496.9160 1487.7262 1487.7290 -2 0 R.ELALEDPMGAITGR.I**  Oxidation (M) ([Ions score 52](http://gary/mascot/cgi/peptide_view.pl?file=../data/20130722/F001265.dat&query=645&hit=1&index=gene_51|putative&px=1&section=5&ave_thresh=3&_ignoreionsscorebelow=20&report=0&_sigthreshold=0.05&_msresflags=1089&_msresflags2=2&percolate=-1&percolate_rt=0))

**105 - 118 744.8726 1487.7306 1487.7290 1 0 R.ELALEDPMGAITGR.I**  Oxidation (M) ([Ions score 80](http://gary/mascot/cgi/peptide_view.pl?file=../data/20130722/F001265.dat&query=646&hit=1&index=gene_51|putative&px=1&section=5&ave_thresh=3&_ignoreionsscorebelow=20&report=0&_sigthreshold=0.05&_msresflags=1089&_msresflags2=2&percolate=-1&percolate_rt=0))

**105 - 129 926.4564 2776.3474 2776.3490 -1 1 R.ELALEDPMGAITGRIGQYWATNNEK.R**  ([Ions score 95](http://gary/mascot/cgi/peptide_view.pl?file=../data/20130722/F001265.dat&query=950&hit=1&index=gene_51|putative&px=1&section=5&ave_thresh=3&_ignoreionsscorebelow=20&report=0&_sigthreshold=0.05&_msresflags=1089&_msresflags2=2&percolate=-1&percolate_rt=0))

**105 - 129 1389.1834 2776.3521 2776.3490 1 1 R.ELALEDPMGAITGRIGQYWATNNEK.R**  ([Ions score 85](http://gary/mascot/cgi/peptide_view.pl?file=../data/20130722/F001265.dat&query=952&hit=1&index=gene_51|putative&px=1&section=5&ave_thresh=3&_ignoreionsscorebelow=20&report=0&_sigthreshold=0.05&_msresflags=1089&_msresflags2=2&percolate=-1&percolate_rt=0))

**105 - 129 695.0955 2776.3530 2776.3490 1 1 R.ELALEDPMGAITGRIGQYWATNNEK.R**  ([Ions score 50](http://gary/mascot/cgi/peptide_view.pl?file=../data/20130722/F001265.dat&query=953&hit=1&index=gene_51|putative&px=1&section=5&ave_thresh=3&_ignoreionsscorebelow=20&report=0&_sigthreshold=0.05&_msresflags=1089&_msresflags2=2&percolate=-1&percolate_rt=0))

**105 - 129 926.7857 2777.3353 2777.3330 1 1 R.ELALEDPMGAITGRIGQYWATNNEK.R** Deamidated(NQ)([Ions score 80](http://gary/mascot/cgi/peptide_view.pl?file=../data/20130722/F001265.dat&query=954&hit=1&index=gene_51|putative&px=1&section=5&ave_thresh=3&_ignoreionsscorebelow=20&report=0&_sigthreshold=0.05&_msresflags=1089&_msresflags2=2&percolate=-1&percolate_rt=0))

**105 - 129 931.7870 2792.3391 2792.3439 -2 1 R.ELALEDPMGAITGRIGQYWATNNEK.R** Oxidation (M) ([Ions score 80](http://gary/mascot/cgi/peptide_view.pl?file=../data/20130722/F001265.dat&query=955&hit=1&index=gene_51|putative&px=1&section=5&ave_thresh=3&_ignoreionsscorebelow=20&report=0&_sigthreshold=0.05&_msresflags=1089&_msresflags2=2&percolate=-1&percolate_rt=0))

**105 - 130 734.1183 2932.4441 2932.4501 -2 2 R.ELALEDPMGAITGRIGQYWATNNEKR.I**  ([Ions score 25](http://gary/mascot/cgi/peptide_view.pl?file=../data/20130722/F001265.dat&query=965&hit=1&index=gene_51|putative&px=1&section=5&ave_thresh=3&_ignoreionsscorebelow=20&report=0&_sigthreshold=0.05&_msresflags=1089&_msresflags2=2&percolate=-1&percolate_rt=0))

**105 - 130 978.4927 2932.4564 2932.4501 2 2 R.ELALEDPMGAITGRIGQYWATNNEKR.I**  ([Ions score 88](http://gary/mascot/cgi/peptide_view.pl?file=../data/20130722/F001265.dat&query=966&hit=1&index=gene_51|putative&px=1&section=5&ave_thresh=3&_ignoreionsscorebelow=20&report=0&_sigthreshold=0.05&_msresflags=1089&_msresflags2=2&percolate=-1&percolate_rt=0))

**119 - 129 662.3167 1322.6189 1322.6255 -5 0 R.IGQYWATNNEK.R**  ([Ions score 59](http://gary/mascot/cgi/peptide_view.pl?file=../data/20130722/F001265.dat&query=543&hit=1&index=gene_51|putative&px=1&section=5&ave_thresh=3&_ignoreionsscorebelow=20&report=0&_sigthreshold=0.05&_msresflags=1089&_msresflags2=2&percolate=-1&percolate_rt=0))

**119 - 129 662.3180 1322.6215 1322.6255 -3 0 R.IGQYWATNNEK.R**  ([Ions score 58](http://gary/mascot/cgi/peptide_view.pl?file=../data/20130722/F001265.dat&query=544&hit=1&index=gene_51|putative&px=1&section=5&ave_thresh=3&_ignoreionsscorebelow=20&report=0&_sigthreshold=0.05&_msresflags=1089&_msresflags2=2&percolate=-1&percolate_rt=0))

**119 - 129 662.3185 1322.6225 1322.6255 -2 0 R.IGQYWATNNEK.R**  ([Ions score 66](http://gary/mascot/cgi/peptide_view.pl?file=../data/20130722/F001265.dat&query=545&hit=1&index=gene_51|putative&px=1&section=5&ave_thresh=3&_ignoreionsscorebelow=20&report=0&_sigthreshold=0.05&_msresflags=1089&_msresflags2=2&percolate=-1&percolate_rt=0))

**119 - 129 441.8815 1322.6227 1322.6255 -2 0 R.IGQYWATNNEK.R**  ([Ions score 24](http://gary/mascot/cgi/peptide_view.pl?file=../data/20130722/F001265.dat&query=546&hit=1&index=gene_51|putative&px=1&section=5&ave_thresh=3&_ignoreionsscorebelow=20&report=0&_sigthreshold=0.05&_msresflags=1089&_msresflags2=2&percolate=-1&percolate_rt=0))

**119 - 129 662.3193 1322.6240 1322.6255 -1 0 R.IGQYWATNNEK.R**  ([Ions score 70](http://gary/mascot/cgi/peptide_view.pl?file=../data/20130722/F001265.dat&query=547&hit=1&index=gene_51|putative&px=1&section=5&ave_thresh=3&_ignoreionsscorebelow=20&report=0&_sigthreshold=0.05&_msresflags=1089&_msresflags2=2&percolate=-1&percolate_rt=0))

**119 - 129 662.3194 1322.6242 1322.6255 -1 0 R.IGQYWATNNEK.R**  ([Ions score 70](http://gary/mascot/cgi/peptide_view.pl?file=../data/20130722/F001265.dat&query=548&hit=1&index=gene_51|putative&px=1&section=5&ave_thresh=3&_ignoreionsscorebelow=20&report=0&_sigthreshold=0.05&_msresflags=1089&_msresflags2=2&percolate=-1&percolate_rt=0))

**119 - 129 662.3195 1322.6244 1322.6255 -1 0 R.IGQYWATNNEK.R**  ([Ions score 57](http://gary/mascot/cgi/peptide_view.pl?file=../data/20130722/F001265.dat&query=549&hit=1&index=gene_51|putative&px=1&section=5&ave_thresh=3&_ignoreionsscorebelow=20&report=0&_sigthreshold=0.05&_msresflags=1089&_msresflags2=2&percolate=-1&percolate_rt=0))

**119 - 129 662.3195 1322.6245 1322.6255 -1 0 R.IGQYWATNNEK.R**  ([Ions score 65](http://gary/mascot/cgi/peptide_view.pl?file=../data/20130722/F001265.dat&query=550&hit=1&index=gene_51|putative&px=1&section=5&ave_thresh=3&_ignoreionsscorebelow=20&report=0&_sigthreshold=0.05&_msresflags=1089&_msresflags2=2&percolate=-1&percolate_rt=0))

**119 - 129 662.3200 1322.6255 1322.6255 0 0 R.IGQYWATNNEK.R**  ([Ions score 57](http://gary/mascot/cgi/peptide_view.pl?file=../data/20130722/F001265.dat&query=551&hit=1&index=gene_51|putative&px=1&section=5&ave_thresh=3&_ignoreionsscorebelow=20&report=0&_sigthreshold=0.05&_msresflags=1089&_msresflags2=2&percolate=-1&percolate_rt=0))

**119 - 129 662.3202 1322.6258 1322.6255 0 0 R.IGQYWATNNEK.R**  ([Ions score 64](http://gary/mascot/cgi/peptide_view.pl?file=../data/20130722/F001265.dat&query=552&hit=1&index=gene_51|putative&px=1&section=5&ave_thresh=3&_ignoreionsscorebelow=20&report=0&_sigthreshold=0.05&_msresflags=1089&_msresflags2=2&percolate=-1&percolate_rt=0))

**119 - 129 662.3203 1322.6261 1322.6255 0 0 R.IGQYWATNNEK.R**  ([Ions score 67](http://gary/mascot/cgi/peptide_view.pl?file=../data/20130722/F001265.dat&query=553&hit=1&index=gene_51|putative&px=1&section=5&ave_thresh=3&_ignoreionsscorebelow=20&report=0&_sigthreshold=0.05&_msresflags=1089&_msresflags2=2&percolate=-1&percolate_rt=0))

**119 - 129 662.3208 1322.6270 1322.6255 1 0 R.IGQYWATNNEK.R**  ([Ions score 62](http://gary/mascot/cgi/peptide_view.pl?file=../data/20130722/F001265.dat&query=554&hit=1&index=gene_51|putative&px=1&section=5&ave_thresh=3&_ignoreionsscorebelow=20&report=0&_sigthreshold=0.05&_msresflags=1089&_msresflags2=2&percolate=-1&percolate_rt=0))

**119 - 129 662.3219 1322.6292 1322.6255 3 0 R.IGQYWATNNEK.R**  ([Ions score 66](http://gary/mascot/cgi/peptide_view.pl?file=../data/20130722/F001265.dat&query=555&hit=1&index=gene_51|putative&px=1&section=5&ave_thresh=3&_ignoreionsscorebelow=20&report=0&_sigthreshold=0.05&_msresflags=1089&_msresflags2=2&percolate=-1&percolate_rt=0))

**119 - 129 662.3221 1322.6296 1322.6255 3 0 R.IGQYWATNNEK.R**  ([Ions score 55](http://gary/mascot/cgi/peptide_view.pl?file=../data/20130722/F001265.dat&query=556&hit=1&index=gene_51|putative&px=1&section=5&ave_thresh=3&_ignoreionsscorebelow=20&report=0&_sigthreshold=0.05&_msresflags=1089&_msresflags2=2&percolate=-1&percolate_rt=0))

**119 - 129 662.3234 1322.6322 1322.6255 5 0 R.IGQYWATNNEK.R**  ([Ions score 54](http://gary/mascot/cgi/peptide_view.pl?file=../data/20130722/F001265.dat&query=557&hit=1&index=gene_51|putative&px=1&section=5&ave_thresh=3&_ignoreionsscorebelow=20&report=0&_sigthreshold=0.05&_msresflags=1089&_msresflags2=2&percolate=-1&percolate_rt=0))

**119 - 129 662.8151 1323.6156 1323.6095 5 0 R.IGQYWATNNEK.R**  Deamidated (NQ) ([Ions score 81](http://gary/mascot/cgi/peptide_view.pl?file=../data/20130722/F001265.dat&query=559&hit=1&index=gene_51|putative&px=1&section=5&ave_thresh=3&_ignoreionsscorebelow=20&report=0&_sigthreshold=0.05&_msresflags=1089&_msresflags2=2&percolate=-1&percolate_rt=0))

**119 - 130 493.9157 1478.7253 1478.7266 -1 1 R.IGQYWATNNEKR.I**  ([Ions score 55](http://gary/mascot/cgi/peptide_view.pl?file=../data/20130722/F001265.dat&query=641&hit=1&index=gene_51|putative&px=1&section=5&ave_thresh=3&_ignoreionsscorebelow=20&report=0&_sigthreshold=0.05&_msresflags=1089&_msresflags2=2&percolate=-1&percolate_rt=0))

**119 - 130 740.3708 1478.7270 1478.7266 0 1 R.IGQYWATNNEKR.I**  ([Ions score 61](http://gary/mascot/cgi/peptide_view.pl?file=../data/20130722/F001265.dat&query=642&hit=1&index=gene_51|putative&px=1&section=5&ave_thresh=3&_ignoreionsscorebelow=20&report=0&_sigthreshold=0.05&_msresflags=1089&_msresflags2=2&percolate=-1&percolate_rt=0))

**119 - 130 494.2448 1479.7126 1479.7106 1 1 R.IGQYWATNNEKR.I**  Deamidated (NQ) ([Ions score 73](http://gary/mascot/cgi/peptide_view.pl?file=../data/20130722/F001265.dat&query=643&hit=1&index=gene_51|putative&px=1&section=5&ave_thresh=3&_ignoreionsscorebelow=20&report=0&_sigthreshold=0.05&_msresflags=1089&_msresflags2=2&percolate=-1&percolate_rt=0))

**119 - 130 740.8660 1479.7174 1479.7106 5 1 R.IGQYWATNNEKR.I**  Deamidated (NQ) ([Ions score 70](http://gary/mascot/cgi/peptide_view.pl?file=../data/20130722/F001265.dat&query=644&hit=1&index=gene_51|putative&px=1&section=5&ave_thresh=3&_ignoreionsscorebelow=20&report=0&_sigthreshold=0.05&_msresflags=1089&_msresflags2=2&percolate=-1&percolate_rt=0))

**130 - 136 436.2750 870.5355 870.5399 -5 1 K.RIIQSVR.G**  ([Ions score 25](http://gary/mascot/cgi/peptide_view.pl?file=../data/20130722/F001265.dat&query=105&hit=1&index=gene_51|putative&px=1&section=5&ave_thresh=3&_ignoreionsscorebelow=20&report=0&_sigthreshold=0.05&_msresflags=1089&_msresflags2=2&percolate=-1&percolate_rt=0))

**130 - 136 436.2772 870.5399 870.5399 0 1 K.RIIQSVR.G**  ([Ions score 43](http://gary/mascot/cgi/peptide_view.pl?file=../data/20130722/F001265.dat&query=107&hit=1&index=gene_51|putative&px=1&section=5&ave_thresh=3&_ignoreionsscorebelow=20&report=0&_sigthreshold=0.05&_msresflags=1089&_msresflags2=2&percolate=-1&percolate_rt=0))

**130 - 136 436.2774 870.5402 870.5399 0 1 K.RIIQSVR.G**  ([Ions score 45](http://gary/mascot/cgi/peptide_view.pl?file=../data/20130722/F001265.dat&query=108&hit=1&index=gene_51|putative&px=1&section=5&ave_thresh=3&_ignoreionsscorebelow=20&report=0&_sigthreshold=0.05&_msresflags=1089&_msresflags2=2&percolate=-1&percolate_rt=0))

**130 - 136 436.2774 870.5402 870.5399 0 1 K.RIIQSVR.G**  ([Ions score 36](http://gary/mascot/cgi/peptide_view.pl?file=../data/20130722/F001265.dat&query=109&hit=1&index=gene_51|putative&px=1&section=5&ave_thresh=3&_ignoreionsscorebelow=20&report=0&_sigthreshold=0.05&_msresflags=1089&_msresflags2=2&percolate=-1&percolate_rt=0))

**130 - 136 436.2775 870.5404 870.5399 1 1 K.RIIQSVR.G**  ([Ions score 45](http://gary/mascot/cgi/peptide_view.pl?file=../data/20130722/F001265.dat&query=110&hit=1&index=gene_51|putative&px=1&section=5&ave_thresh=3&_ignoreionsscorebelow=20&report=0&_sigthreshold=0.05&_msresflags=1089&_msresflags2=2&percolate=-1&percolate_rt=0))

**130 - 136 436.2777 870.5408 870.5399 1 1 K.RIIQSVR.G**  ([Ions score 44](http://gary/mascot/cgi/peptide_view.pl?file=../data/20130722/F001265.dat&query=111&hit=1&index=gene_51|putative&px=1&section=5&ave_thresh=3&_ignoreionsscorebelow=20&report=0&_sigthreshold=0.05&_msresflags=1089&_msresflags2=2&percolate=-1&percolate_rt=0))

**130 - 136 436.2779 870.5412 870.5399 2 1 K.RIIQSVR.G**  ([Ions score 38](http://gary/mascot/cgi/peptide_view.pl?file=../data/20130722/F001265.dat&query=112&hit=1&index=gene_51|putative&px=1&section=5&ave_thresh=3&_ignoreionsscorebelow=20&report=0&_sigthreshold=0.05&_msresflags=1089&_msresflags2=2&percolate=-1&percolate_rt=0))

**130 - 136 436.2783 870.5420 870.5399 2 1 K.RIIQSVR.G**  ([Ions score 34](http://gary/mascot/cgi/peptide_view.pl?file=../data/20130722/F001265.dat&query=113&hit=1&index=gene_51|putative&px=1&section=5&ave_thresh=3&_ignoreionsscorebelow=20&report=0&_sigthreshold=0.05&_msresflags=1089&_msresflags2=2&percolate=-1&percolate_rt=0))

**130 - 136 436.2789 870.5432 870.5399 4 1 K.RIIQSVR.G**  ([Ions score 36](http://gary/mascot/cgi/peptide_view.pl?file=../data/20130722/F001265.dat&query=114&hit=1&index=gene_51|putative&px=1&section=5&ave_thresh=3&_ignoreionsscorebelow=20&report=0&_sigthreshold=0.05&_msresflags=1089&_msresflags2=2&percolate=-1&percolate_rt=0))

**131 - 136 358.2265 714.4384 714.4388 -1 0 R.IIQSVR.G**  ([Ions score 25](http://gary/mascot/cgi/peptide_view.pl?file=../data/20130722/F001265.dat&query=14&hit=1&index=gene_51|putative&px=1&section=5&ave_thresh=3&_ignoreionsscorebelow=20&report=0&_sigthreshold=0.05&_msresflags=1089&_msresflags2=2&percolate=-1&percolate_rt=0))

**131 - 136 358.2267 714.4389 714.4388 0 0 R.IIQSVR.G**  ([Ions score 20](http://gary/mascot/cgi/peptide_view.pl?file=../data/20130722/F001265.dat&query=17&hit=1&index=gene_51|putative&px=1&section=5&ave_thresh=3&_ignoreionsscorebelow=20&report=0&_sigthreshold=0.05&_msresflags=1089&_msresflags2=2&percolate=-1&percolate_rt=0))

**131 - 136 358.2271 714.4397 714.4388 1 0 R.IIQSVR.G**  ([Ions score 21](http://gary/mascot/cgi/peptide_view.pl?file=../data/20130722/F001265.dat&query=20&hit=1&index=gene_51|putative&px=1&section=5&ave_thresh=3&_ignoreionsscorebelow=20&report=0&_sigthreshold=0.05&_msresflags=1089&_msresflags2=2&percolate=-1&percolate_rt=0))

**137 - 167 1041.8215 3122.4428 3122.4462 -1 0 R.GLIADNVANDGGDMLFDISAATDTAVTDANR.A**  ([Ions score 98](http://gary/mascot/cgi/peptide_view.pl?file=../data/20130722/F001265.dat&query=985&hit=1&index=gene_51|putative&px=1&section=5&ave_thresh=3&_ignoreionsscorebelow=20&report=0&_sigthreshold=0.05&_msresflags=1089&_msresflags2=2&percolate=-1&percolate_rt=0))

**137 - 167 781.6182 3122.4438 3122.4462 -1 0 R.GLIADNVANDGGDMLFDISAATDTAVTDANR.A**  ([Ions score 79](http://gary/mascot/cgi/peptide_view.pl?file=../data/20130722/F001265.dat&query=986&hit=1&index=gene_51|putative&px=1&section=5&ave_thresh=3&_ignoreionsscorebelow=20&report=0&_sigthreshold=0.05&_msresflags=1089&_msresflags2=2&percolate=-1&percolate_rt=0))

**137 - 167 785.6168 3138.4379 3138.4412 -1 0 R.GLIADNVANDGGDMLFDISAATDTAVTDANR.A**

Oxidation (M) ([Ions score 75](http://gary/mascot/cgi/peptide_view.pl?file=../data/20130722/F001265.dat&query=987&hit=1&index=gene_51|putative&px=1&section=5&ave_thresh=3&_ignoreionsscorebelow=20&report=0&_sigthreshold=0.05&_msresflags=1089&_msresflags2=2&percolate=-1&percolate_rt=0))

**137 - 167 1047.1550 3138.4433 3138.4412 1 0 R.GLIADNVANDGGDMLFDISAATDTAVTDANR.A**

Oxidation (M) ([Ions score 85](http://gary/mascot/cgi/peptide_view.pl?file=../data/20130722/F001265.dat&query=988&hit=1&index=gene_51|putative&px=1&section=5&ave_thresh=3&_ignoreionsscorebelow=20&report=0&_sigthreshold=0.05&_msresflags=1089&_msresflags2=2&percolate=-1&percolate_rt=0))

**168 - 198 830.1458 3316.5541 3316.5526 0 0 R.ADADAIIDTVQTMGDHGEMLSAIAMHSVVYR.R**  ([Ions score 44](http://gary/mascot/cgi/peptide_view.pl?file=../data/20130722/F001265.dat&query=1002&hit=1&index=gene_51|putative&px=1&section=5&ave_thresh=3&_ignoreionsscorebelow=20&report=0&_sigthreshold=0.05&_msresflags=1089&_msresflags2=2&percolate=-1&percolate_rt=0))

**168 - 198 834.1459 3332.5544 3332.5475 2 0 R.ADADAIIDTVQTMGDHGEMLSAIAMHSVVYR.R**

Oxidation (M) ([Ions score 32](http://gary/mascot/cgi/peptide_view.pl?file=../data/20130722/F001265.dat&query=1005&hit=1&index=gene_51|putative&px=1&section=5&ave_thresh=3&_ignoreionsscorebelow=20&report=0&_sigthreshold=0.05&_msresflags=1089&_msresflags2=2&percolate=-1&percolate_rt=0))

**168 - 198 838.1432 3348.5437 3348.5425 0 0 R.ADADAIIDTVQTMGDHGEMLSAIAMHSVVYR.R**

2 Oxidation (M) ([Ions score 22](http://gary/mascot/cgi/peptide_view.pl?file=../data/20130722/F001265.dat&query=1006&hit=1&index=gene_51|putative&px=1&section=5&ave_thresh=3&_ignoreionsscorebelow=20&report=0&_sigthreshold=0.05&_msresflags=1089&_msresflags2=2&percolate=-1&percolate_rt=0))

**168 - 198 838.1456 3348.5534 3348.5425 3 0 R.ADADAIIDTVQTMGDHGEMLSAIAMHSVVYR.R**

2 Oxidation (M) ([Ions score 31](http://gary/mascot/cgi/peptide_view.pl?file=../data/20130722/F001265.dat&query=1007&hit=1&index=gene_51|putative&px=1&section=5&ave_thresh=3&_ignoreionsscorebelow=20&report=0&_sigthreshold=0.05&_msresflags=1089&_msresflags2=2&percolate=-1&percolate_rt=0))

**203 - 213 642.8327 1283.6508 1283.6510 0 0 K.QNLIDFIPDAR.G**  Gln->pyro-Glu (N-term Q) ([Ions score 41](http://gary/mascot/cgi/peptide_view.pl?file=../data/20130722/F001265.dat&query=486&hit=1&index=gene_51|putative&px=1&section=5&ave_thresh=3&_ignoreionsscorebelow=20&report=0&_sigthreshold=0.05&_msresflags=1089&_msresflags2=2&percolate=-1&percolate_rt=0))

**203 - 213 651.3439 1300.6732 1300.6775 -3 0 K.QNLIDFIPDAR.G**  ([Ions score 74](http://gary/mascot/cgi/peptide_view.pl?file=../data/20130722/F001265.dat&query=504&hit=1&index=gene_51|putative&px=1&section=5&ave_thresh=3&_ignoreionsscorebelow=20&report=0&_sigthreshold=0.05&_msresflags=1089&_msresflags2=2&percolate=-1&percolate_rt=0))

**203 - 213 651.3448 1300.6750 1300.6775 -2 0 K.QNLIDFIPDAR.G**  ([Ions score 87](http://gary/mascot/cgi/peptide_view.pl?file=../data/20130722/F001265.dat&query=505&hit=1&index=gene_51|putative&px=1&section=5&ave_thresh=3&_ignoreionsscorebelow=20&report=0&_sigthreshold=0.05&_msresflags=1089&_msresflags2=2&percolate=-1&percolate_rt=0))

**203 - 213 651.3448 1300.6751 1300.6775 -2 0 K.QNLIDFIPDAR.G**  ([Ions score 57](http://gary/mascot/cgi/peptide_view.pl?file=../data/20130722/F001265.dat&query=506&hit=1&index=gene_51|putative&px=1&section=5&ave_thresh=3&_ignoreionsscorebelow=20&report=0&_sigthreshold=0.05&_msresflags=1089&_msresflags2=2&percolate=-1&percolate_rt=0))

**203 - 213 651.3456 1300.6766 1300.6775 -1 0 K.QNLIDFIPDAR.G**  ([Ions score 92](http://gary/mascot/cgi/peptide_view.pl?file=../data/20130722/F001265.dat&query=508&hit=1&index=gene_51|putative&px=1&section=5&ave_thresh=3&_ignoreionsscorebelow=20&report=0&_sigthreshold=0.05&_msresflags=1089&_msresflags2=2&percolate=-1&percolate_rt=0))

**203 - 213 651.3456 1300.6766 1300.6775 -1 0 K.QNLIDFIPDAR.G**  ([Ions score 64](http://gary/mascot/cgi/peptide_view.pl?file=../data/20130722/F001265.dat&query=509&hit=1&index=gene_51|putative&px=1&section=5&ave_thresh=3&_ignoreionsscorebelow=20&report=0&_sigthreshold=0.05&_msresflags=1089&_msresflags2=2&percolate=-1&percolate_rt=0))

**203 - 213 434.5662 1300.6768 1300.6775 -1 0 K.QNLIDFIPDAR.G**  ([Ions score 33](http://gary/mascot/cgi/peptide_view.pl?file=../data/20130722/F001265.dat&query=510&hit=1&index=gene_51|putative&px=1&section=5&ave_thresh=3&_ignoreionsscorebelow=20&report=0&_sigthreshold=0.05&_msresflags=1089&_msresflags2=2&percolate=-1&percolate_rt=0))

**203 - 213 651.3460 1300.6773 1300.6775 0 0 K.QNLIDFIPDAR.G**  ([Ions score 83](http://gary/mascot/cgi/peptide_view.pl?file=../data/20130722/F001265.dat&query=511&hit=1&index=gene_51|putative&px=1&section=5&ave_thresh=3&_ignoreionsscorebelow=20&report=0&_sigthreshold=0.05&_msresflags=1089&_msresflags2=2&percolate=-1&percolate_rt=0))

**203 - 213 651.3461 1300.6776 1300.6775 0 0 K.QNLIDFIPDAR.G**  ([Ions score 45](http://gary/mascot/cgi/peptide_view.pl?file=../data/20130722/F001265.dat&query=512&hit=1&index=gene_51|putative&px=1&section=5&ave_thresh=3&_ignoreionsscorebelow=20&report=0&_sigthreshold=0.05&_msresflags=1089&_msresflags2=2&percolate=-1&percolate_rt=0))

**203 - 213 651.3466 1300.6786 1300.6775 1 0 K.QNLIDFIPDAR.G**  ([Ions score 60](http://gary/mascot/cgi/peptide_view.pl?file=../data/20130722/F001265.dat&query=513&hit=1&index=gene_51|putative&px=1&section=5&ave_thresh=3&_ignoreionsscorebelow=20&report=0&_sigthreshold=0.05&_msresflags=1089&_msresflags2=2&percolate=-1&percolate_rt=0))

**203 - 213 651.3467 1300.6789 1300.6775 1 0 K.QNLIDFIPDAR.G**  ([Ions score 82](http://gary/mascot/cgi/peptide_view.pl?file=../data/20130722/F001265.dat&query=514&hit=1&index=gene_51|putative&px=1&section=5&ave_thresh=3&_ignoreionsscorebelow=20&report=0&_sigthreshold=0.05&_msresflags=1089&_msresflags2=2&percolate=-1&percolate_rt=0))

**203 - 213 651.3472 1300.6799 1300.6775 2 0 K.QNLIDFIPDAR.G**  ([Ions score 75](http://gary/mascot/cgi/peptide_view.pl?file=../data/20130722/F001265.dat&query=515&hit=1&index=gene_51|putative&px=1&section=5&ave_thresh=3&_ignoreionsscorebelow=20&report=0&_sigthreshold=0.05&_msresflags=1089&_msresflags2=2&percolate=-1&percolate_rt=0))

**203 - 213 651.3476 1300.6806 1300.6775 2 0 K.QNLIDFIPDAR.G**  ([Ions score 63](http://gary/mascot/cgi/peptide_view.pl?file=../data/20130722/F001265.dat&query=516&hit=1&index=gene_51|putative&px=1&section=5&ave_thresh=3&_ignoreionsscorebelow=20&report=0&_sigthreshold=0.05&_msresflags=1089&_msresflags2=2&percolate=-1&percolate_rt=0))

**203 - 213 651.8388 1301.6631 1301.6615 1 0 K.QNLIDFIPDAR.G**  Deamidated (NQ) ([Ions score 73](http://gary/mascot/cgi/peptide_view.pl?file=../data/20130722/F001265.dat&query=519&hit=1&index=gene_51|putative&px=1&section=5&ave_thresh=3&_ignoreionsscorebelow=20&report=0&_sigthreshold=0.05&_msresflags=1089&_msresflags2=2&percolate=-1&percolate_rt=0))

**203 - 224 1251.1277 2500.2408 2500.2420 0 1 K.QNLIDFIPDARGEVNIPMYQGK.T**

Gln->pyro-Glu (N-term Q) ([Ions score 66](http://gary/mascot/cgi/peptide_view.pl?file=../data/20130722/F001265.dat&query=849&hit=1&index=gene_51|putative&px=1&section=5&ave_thresh=3&_ignoreionsscorebelow=20&report=0&_sigthreshold=0.05&_msresflags=1089&_msresflags2=2&percolate=-1&percolate_rt=0))

**203 - 224 834.4230 2500.2473 2500.2420 2 1 K.QNLIDFIPDARGEVNIPMYQGK.T**

Gln->pyro-Glu (N-term Q) ([Ions score 69](http://gary/mascot/cgi/peptide_view.pl?file=../data/20130722/F001265.dat&query=850&hit=1&index=gene_51|putative&px=1&section=5&ave_thresh=3&_ignoreionsscorebelow=20&report=0&_sigthreshold=0.05&_msresflags=1089&_msresflags2=2&percolate=-1&percolate_rt=0))

**203 - 224 839.7531 2516.2375 2516.2370 0 1 K.QNLIDFIPDARGEVNIPMYQGK.T**

Gln->pyro-Glu (N-term Q); Oxidation (M) ([Ions score 68](http://gary/mascot/cgi/peptide_view.pl?file=../data/20130722/F001265.dat&query=856&hit=1&index=gene_51|putative&px=1&section=5&ave_thresh=3&_ignoreionsscorebelow=20&report=0&_sigthreshold=0.05&_msresflags=1089&_msresflags2=2&percolate=-1&percolate_rt=0))

**203 - 224 1259.1279 2516.2413 2516.2370 2 1 K.QNLIDFIPDARGEVNIPMYQGK.T**

Gln->pyro-Glu (N-term Q); Oxidation (M) ([Ions score 49](http://gary/mascot/cgi/peptide_view.pl?file=../data/20130722/F001265.dat&query=857&hit=1&index=gene_51|putative&px=1&section=5&ave_thresh=3&_ignoreionsscorebelow=20&report=0&_sigthreshold=0.05&_msresflags=1089&_msresflags2=2&percolate=-1&percolate_rt=0))

**203 - 224 840.0936 2517.2591 2517.2686 -4 1 K.QNLIDFIPDARGEVNIPMYQGK.T**  ([Ions score 32](http://gary/mascot/cgi/peptide_view.pl?file=../data/20130722/F001265.dat&query=858&hit=1&index=gene_51|putative&px=1&section=5&ave_thresh=3&_ignoreionsscorebelow=20&report=0&_sigthreshold=0.05&_msresflags=1089&_msresflags2=2&percolate=-1&percolate_rt=0))

**203 - 224 840.0956 2517.2651 2517.2686 -1 1 K.QNLIDFIPDARGEVNIPMYQGK.T**  ([Ions score 39](http://gary/mascot/cgi/peptide_view.pl?file=../data/20130722/F001265.dat&query=859&hit=1&index=gene_51|putative&px=1&section=5&ave_thresh=3&_ignoreionsscorebelow=20&report=0&_sigthreshold=0.05&_msresflags=1089&_msresflags2=2&percolate=-1&percolate_rt=0))

**203 - 224 1259.6414 2517.2682 2517.2686 0 1 K.QNLIDFIPDARGEVNIPMYQGK.T**  ([Ions score 79](http://gary/mascot/cgi/peptide_view.pl?file=../data/20130722/F001265.dat&query=860&hit=1&index=gene_51|putative&px=1&section=5&ave_thresh=3&_ignoreionsscorebelow=20&report=0&_sigthreshold=0.05&_msresflags=1089&_msresflags2=2&percolate=-1&percolate_rt=0))

**203 - 224 840.0975 2517.2708 2517.2686 1 1 K.QNLIDFIPDARGEVNIPMYQGK.T**  ([Ions score 47](http://gary/mascot/cgi/peptide_view.pl?file=../data/20130722/F001265.dat&query=862&hit=1&index=gene_51|putative&px=1&section=5&ave_thresh=3&_ignoreionsscorebelow=20&report=0&_sigthreshold=0.05&_msresflags=1089&_msresflags2=2&percolate=-1&percolate_rt=0))

**203 - 224 840.0980 2517.2721 2517.2686 1 1 K.QNLIDFIPDARGEVNIPMYQGK.T**  ([Ions score 43](http://gary/mascot/cgi/peptide_view.pl?file=../data/20130722/F001265.dat&query=863&hit=1&index=gene_51|putative&px=1&section=5&ave_thresh=3&_ignoreionsscorebelow=20&report=0&_sigthreshold=0.05&_msresflags=1089&_msresflags2=2&percolate=-1&percolate_rt=0))

**203 - 224 840.0983 2517.2732 2517.2686 2 1 K.QNLIDFIPDARGEVNIPMYQGK.T**  ([Ions score 42](http://gary/mascot/cgi/peptide_view.pl?file=../data/20130722/F001265.dat&query=864&hit=1&index=gene_51|putative&px=1&section=5&ave_thresh=3&_ignoreionsscorebelow=20&report=0&_sigthreshold=0.05&_msresflags=1089&_msresflags2=2&percolate=-1&percolate_rt=0))

**203 - 224 840.0993 2517.2761 2517.2686 3 1 K.QNLIDFIPDARGEVNIPMYQGK.T**  ([Ions score 65](http://gary/mascot/cgi/peptide_view.pl?file=../data/20130722/F001265.dat&query=865&hit=1&index=gene_51|putative&px=1&section=5&ave_thresh=3&_ignoreionsscorebelow=20&report=0&_sigthreshold=0.05&_msresflags=1089&_msresflags2=2&percolate=-1&percolate_rt=0))

**203 - 224 840.1000 2517.2783 2517.2686 4 1 K.QNLIDFIPDARGEVNIPMYQGK.T**  ([Ions score 58](http://gary/mascot/cgi/peptide_view.pl?file=../data/20130722/F001265.dat&query=866&hit=1&index=gene_51|putative&px=1&section=5&ave_thresh=3&_ignoreionsscorebelow=20&report=0&_sigthreshold=0.05&_msresflags=1089&_msresflags2=2&percolate=-1&percolate_rt=0))

**203 - 224 840.4284 2518.2634 2518.2526 4 1 K.QNLIDFIPDARGEVNIPMYQGK.T**  Deamidated (NQ) ([Ions score 55](http://gary/mascot/cgi/peptide_view.pl?file=../data/20130722/F001265.dat&query=867&hit=1&index=gene_51|putative&px=1&section=5&ave_thresh=3&_ignoreionsscorebelow=20&report=0&_sigthreshold=0.05&_msresflags=1089&_msresflags2=2&percolate=-1&percolate_rt=0))

**203 - 224 630.5751 2518.2714 2518.2526 7 1 K.QNLIDFIPDARGEVNIPMYQGK.T**  Deamidated (NQ) ([Ions score 26](http://gary/mascot/cgi/peptide_view.pl?file=../data/20130722/F001265.dat&query=868&hit=1&index=gene_51|putative&px=1&section=5&ave_thresh=3&_ignoreionsscorebelow=20&report=0&_sigthreshold=0.05&_msresflags=1089&_msresflags2=2&percolate=-1&percolate_rt=0))

**203 - 224 845.4282 2533.2628 2533.2635 0 1 K.QNLIDFIPDARGEVNIPMYQGK.T**  Oxidation (M) ([Ions score 25](http://gary/mascot/cgi/peptide_view.pl?file=../data/20130722/F001265.dat&query=873&hit=1&index=gene_51|putative&px=1&section=5&ave_thresh=3&_ignoreionsscorebelow=20&report=0&_sigthreshold=0.05&_msresflags=1089&_msresflags2=2&percolate=-1&percolate_rt=0))

**203 - 224 1267.6393 2533.2640 2533.2635 0 1 K.QNLIDFIPDARGEVNIPMYQGK.T**  Oxidation (M) ([Ions score 68](http://gary/mascot/cgi/peptide_view.pl?file=../data/20130722/F001265.dat&query=874&hit=1&index=gene_51|putative&px=1&section=5&ave_thresh=3&_ignoreionsscorebelow=20&report=0&_sigthreshold=0.05&_msresflags=1089&_msresflags2=2&percolate=-1&percolate_rt=0))

**203 - 224 845.4293 2533.2660 2533.2635 1 1 K.QNLIDFIPDARGEVNIPMYQGK.T**  Oxidation (M) ([Ions score 36](http://gary/mascot/cgi/peptide_view.pl?file=../data/20130722/F001265.dat&query=875&hit=1&index=gene_51|putative&px=1&section=5&ave_thresh=3&_ignoreionsscorebelow=20&report=0&_sigthreshold=0.05&_msresflags=1089&_msresflags2=2&percolate=-1&percolate_rt=0))

**214 - 224 618.3027 1234.5908 1234.6016 -9 0 R.GEVNIPMYQGK.T**  ([Ions score 57](http://gary/mascot/cgi/peptide_view.pl?file=../data/20130722/F001265.dat&query=420&hit=1&index=gene_51|putative&px=1&section=5&ave_thresh=3&_ignoreionsscorebelow=20&report=0&_sigthreshold=0.05&_msresflags=1089&_msresflags2=2&percolate=-1&percolate_rt=0))

**214 - 224 618.3029 1234.5912 1234.6016 -8 0 R.GEVNIPMYQGK.T**  ([Ions score 52](http://gary/mascot/cgi/peptide_view.pl?file=../data/20130722/F001265.dat&query=421&hit=1&index=gene_51|putative&px=1&section=5&ave_thresh=3&_ignoreionsscorebelow=20&report=0&_sigthreshold=0.05&_msresflags=1089&_msresflags2=2&percolate=-1&percolate_rt=0))

**214 - 224 618.3057 1234.5968 1234.6016 -4 0 R.GEVNIPMYQGK.T**  ([Ions score 40](http://gary/mascot/cgi/peptide_view.pl?file=../data/20130722/F001265.dat&query=422&hit=1&index=gene_51|putative&px=1&section=5&ave_thresh=3&_ignoreionsscorebelow=20&report=0&_sigthreshold=0.05&_msresflags=1089&_msresflags2=2&percolate=-1&percolate_rt=0))

**214 - 224 618.3071 1234.5996 1234.6016 -2 0 R.GEVNIPMYQGK.T**  ([Ions score 54](http://gary/mascot/cgi/peptide_view.pl?file=../data/20130722/F001265.dat&query=423&hit=1&index=gene_51|putative&px=1&section=5&ave_thresh=3&_ignoreionsscorebelow=20&report=0&_sigthreshold=0.05&_msresflags=1089&_msresflags2=2&percolate=-1&percolate_rt=0))

**214 - 224 618.3074 1234.6003 1234.6016 -1 0 R.GEVNIPMYQGK.T**  ([Ions score 42](http://gary/mascot/cgi/peptide_view.pl?file=../data/20130722/F001265.dat&query=424&hit=1&index=gene_51|putative&px=1&section=5&ave_thresh=3&_ignoreionsscorebelow=20&report=0&_sigthreshold=0.05&_msresflags=1089&_msresflags2=2&percolate=-1&percolate_rt=0))

**214 - 224 618.3076 1234.6006 1234.6016 -1 0 R.GEVNIPMYQGK.T**  ([Ions score 61](http://gary/mascot/cgi/peptide_view.pl?file=../data/20130722/F001265.dat&query=425&hit=1&index=gene_51|putative&px=1&section=5&ave_thresh=3&_ignoreionsscorebelow=20&report=0&_sigthreshold=0.05&_msresflags=1089&_msresflags2=2&percolate=-1&percolate_rt=0))

**214 - 224 412.5409 1234.6008 1234.6016 -1 0 R.GEVNIPMYQGK.T**  ([Ions score 42](http://gary/mascot/cgi/peptide_view.pl?file=../data/20130722/F001265.dat&query=426&hit=1&index=gene_51|putative&px=1&section=5&ave_thresh=3&_ignoreionsscorebelow=20&report=0&_sigthreshold=0.05&_msresflags=1089&_msresflags2=2&percolate=-1&percolate_rt=0))

**214 - 224 618.3081 1234.6017 1234.6016 0 0 R.GEVNIPMYQGK.T**  ([Ions score 55](http://gary/mascot/cgi/peptide_view.pl?file=../data/20130722/F001265.dat&query=427&hit=1&index=gene_51|putative&px=1&section=5&ave_thresh=3&_ignoreionsscorebelow=20&report=0&_sigthreshold=0.05&_msresflags=1089&_msresflags2=2&percolate=-1&percolate_rt=0))

**214 - 224 618.3093 1234.6041 1234.6016 2 0 R.GEVNIPMYQGK.T**  ([Ions score 53](http://gary/mascot/cgi/peptide_view.pl?file=../data/20130722/F001265.dat&query=428&hit=1&index=gene_51|putative&px=1&section=5&ave_thresh=3&_ignoreionsscorebelow=20&report=0&_sigthreshold=0.05&_msresflags=1089&_msresflags2=2&percolate=-1&percolate_rt=0))

**214 - 224 618.3101 1234.6057 1234.6016 3 0 R.GEVNIPMYQGK.T**  ([Ions score 54](http://gary/mascot/cgi/peptide_view.pl?file=../data/20130722/F001265.dat&query=429&hit=1&index=gene_51|putative&px=1&section=5&ave_thresh=3&_ignoreionsscorebelow=20&report=0&_sigthreshold=0.05&_msresflags=1089&_msresflags2=2&percolate=-1&percolate_rt=0))

**214 - 224 618.3102 1234.6059 1234.6016 4 0 R.GEVNIPMYQGK.T**  ([Ions score 64](http://gary/mascot/cgi/peptide_view.pl?file=../data/20130722/F001265.dat&query=430&hit=1&index=gene_51|putative&px=1&section=5&ave_thresh=3&_ignoreionsscorebelow=20&report=0&_sigthreshold=0.05&_msresflags=1089&_msresflags2=2&percolate=-1&percolate_rt=0))

**214 - 224 618.3105 1234.6064 1234.6016 4 0 R.GEVNIPMYQGK.T**  ([Ions score 69](http://gary/mascot/cgi/peptide_view.pl?file=../data/20130722/F001265.dat&query=431&hit=1&index=gene_51|putative&px=1&section=5&ave_thresh=3&_ignoreionsscorebelow=20&report=0&_sigthreshold=0.05&_msresflags=1089&_msresflags2=2&percolate=-1&percolate_rt=0))

**214 - 224 618.8055 1235.5965 1235.5856 9 0 R.GEVNIPMYQGK.T**  Deamidated (NQ) ([Ions score 52](http://gary/mascot/cgi/peptide_view.pl?file=../data/20130722/F001265.dat&query=433&hit=1&index=gene_51|putative&px=1&section=5&ave_thresh=3&_ignoreionsscorebelow=20&report=0&_sigthreshold=0.05&_msresflags=1089&_msresflags2=2&percolate=-1&percolate_rt=0))

**214 - 224 626.3020 1250.5894 1250.5965 -6 0 R.GEVNIPMYQGK.T**  Oxidation (M) ([Ions score 44](http://gary/mascot/cgi/peptide_view.pl?file=../data/20130722/F001265.dat&query=449&hit=1&index=gene_51|putative&px=1&section=5&ave_thresh=3&_ignoreionsscorebelow=20&report=0&_sigthreshold=0.05&_msresflags=1089&_msresflags2=2&percolate=-1&percolate_rt=0))

**214 - 224 417.8734 1250.5984 1250.5965 2 0 R.GEVNIPMYQGK.T**  Oxidation (M) ([Ions score 25](http://gary/mascot/cgi/peptide_view.pl?file=../data/20130722/F001265.dat&query=451&hit=1&index=gene_51|putative&px=1&section=5&ave_thresh=3&_ignoreionsscorebelow=20&report=0&_sigthreshold=0.05&_msresflags=1089&_msresflags2=2&percolate=-1&percolate_rt=0))

**214 - 224 626.3065 1250.5985 1250.5965 2 0 R.GEVNIPMYQGK.T**  Oxidation (M) ([Ions score 45](http://gary/mascot/cgi/peptide_view.pl?file=../data/20130722/F001265.dat&query=452&hit=1&index=gene_51|putative&px=1&section=5&ave_thresh=3&_ignoreionsscorebelow=20&report=0&_sigthreshold=0.05&_msresflags=1089&_msresflags2=2&percolate=-1&percolate_rt=0))

**214 - 224 626.3068 1250.5990 1250.5965 2 0 R.GEVNIPMYQGK.T**  Oxidation (M) ([Ions score 53](http://gary/mascot/cgi/peptide_view.pl?file=../data/20130722/F001265.dat&query=453&hit=1&index=gene_51|putative&px=1&section=5&ave_thresh=3&_ignoreionsscorebelow=20&report=0&_sigthreshold=0.05&_msresflags=1089&_msresflags2=2&percolate=-1&percolate_rt=0))

**258 - 271 721.3676 1440.7207 1440.7209 0 0 R.LGEGQPTTPSAIDR.V**  ([Ions score 62](http://gary/mascot/cgi/peptide_view.pl?file=../data/20130722/F001265.dat&query=621&hit=1&index=gene_51|putative&px=1&section=5&ave_thresh=3&_ignoreionsscorebelow=20&report=0&_sigthreshold=0.05&_msresflags=1089&_msresflags2=2&percolate=-1&percolate_rt=0))

**258 - 287 1515.2439 3028.4732 3028.4850 -4 1 R.LGEGQPTTPSAIDRVEAAGNGGGQDIIYSR.R**  ([Ions score 88](http://gary/mascot/cgi/peptide_view.pl?file=../data/20130722/F001265.dat&query=972&hit=1&index=gene_51|putative&px=1&section=5&ave_thresh=3&_ignoreionsscorebelow=20&report=0&_sigthreshold=0.05&_msresflags=1089&_msresflags2=2&percolate=-1&percolate_rt=0))

**258 - 287 1010.5020 3028.4842 3028.4850 0 1 R.LGEGQPTTPSAIDRVEAAGNGGGQDIIYSR.R**  ([Ions score 87](http://gary/mascot/cgi/peptide_view.pl?file=../data/20130722/F001265.dat&query=973&hit=1&index=gene_51|putative&px=1&section=5&ave_thresh=3&_ignoreionsscorebelow=20&report=0&_sigthreshold=0.05&_msresflags=1089&_msresflags2=2&percolate=-1&percolate_rt=0))

**258 - 287 758.1293 3028.4882 3028.4850 1 1 R.LGEGQPTTPSAIDRVEAAGNGGGQDIIYSR.R**  ([Ions score 60](http://gary/mascot/cgi/peptide_view.pl?file=../data/20130722/F001265.dat&query=974&hit=1&index=gene_51|putative&px=1&section=5&ave_thresh=3&_ignoreionsscorebelow=20&report=0&_sigthreshold=0.05&_msresflags=1089&_msresflags2=2&percolate=-1&percolate_rt=0))

**258 - 287 1010.8304 3029.4695 3029.4690 0 1 R.LGEGQPTTPSAIDRVEAAGNGGGQDIIYSR.R**

Deamidated (NQ) ([Ions score 85](http://gary/mascot/cgi/peptide_view.pl?file=../data/20130722/F001265.dat&query=975&hit=1&index=gene_51|putative&px=1&section=5&ave_thresh=3&_ignoreionsscorebelow=20&report=0&_sigthreshold=0.05&_msresflags=1089&_msresflags2=2&percolate=-1&percolate_rt=0))

**258 - 287 1010.8314 3029.4724 3029.4690 1 1 R.LGEGQPTTPSAIDRVEAAGNGGGQDIIYSR.R**

Deamidated (NQ) ([Ions score 64](http://gary/mascot/cgi/peptide_view.pl?file=../data/20130722/F001265.dat&query=976&hit=1&index=gene_51|putative&px=1&section=5&ave_thresh=3&_ignoreionsscorebelow=20&report=0&_sigthreshold=0.05&_msresflags=1089&_msresflags2=2&percolate=-1&percolate_rt=0))

**258 - 287 758.3757 3029.4738 3029.4690 2 1 R.LGEGQPTTPSAIDRVEAAGNGGGQDIIYSR.R**

Deamidated (NQ) ([Ions score 72](http://gary/mascot/cgi/peptide_view.pl?file=../data/20130722/F001265.dat&query=977&hit=1&index=gene_51|putative&px=1&section=5&ave_thresh=3&_ignoreionsscorebelow=20&report=0&_sigthreshold=0.05&_msresflags=1089&_msresflags2=2&percolate=-1&percolate_rt=0))

**258 - 287 1515.7474 3029.4803 3029.4690 4 1 R.LGEGQPTTPSAIDRVEAAGNGGGQDIIYSR.R**

Deamidated (NQ) ([Ions score 80](http://gary/mascot/cgi/peptide_view.pl?file=../data/20130722/F001265.dat&query=978&hit=1&index=gene_51|putative&px=1&section=5&ave_thresh=3&_ignoreionsscorebelow=20&report=0&_sigthreshold=0.05&_msresflags=1089&_msresflags2=2&percolate=-1&percolate_rt=0))

**258 - 288 1062.5388 3184.5946 3184.5861 3 2 R.LGEGQPTTPSAIDRVEAAGNGGGQDIIYSRR.S**  ([Ions score 53](http://gary/mascot/cgi/peptide_view.pl?file=../data/20130722/F001265.dat&query=994&hit=1&index=gene_51|putative&px=1&section=5&ave_thresh=3&_ignoreionsscorebelow=20&report=0&_sigthreshold=0.05&_msresflags=1089&_msresflags2=2&percolate=-1&percolate_rt=0))

**258 - 288 797.3998 3185.5703 3185.5701 0 2 R.LGEGQPTTPSAIDRVEAAGNGGGQDIIYSRR.S**

Deamidated (NQ) ([Ions score 28](http://gary/mascot/cgi/peptide_view.pl?file=../data/20130722/F001265.dat&query=995&hit=1&index=gene_51|putative&px=1&section=5&ave_thresh=3&_ignoreionsscorebelow=20&report=0&_sigthreshold=0.05&_msresflags=1089&_msresflags2=2&percolate=-1&percolate_rt=0))

**258 - 288 1062.8668 3185.5786 3185.5701 3 2 R.LGEGQPTTPSAIDRVEAAGNGGGQDIIYSRR.S**

Deamidated (NQ) ([Ions score 54](http://gary/mascot/cgi/peptide_view.pl?file=../data/20130722/F001265.dat&query=996&hit=1&index=gene_51|putative&px=1&section=5&ave_thresh=3&_ignoreionsscorebelow=20&report=0&_sigthreshold=0.05&_msresflags=1089&_msresflags2=2&percolate=-1&percolate_rt=0))

**272 - 287 803.8938 1605.7730 1605.7747 -1 0 R.VEAAGNGGGQDIIYSR.R**  ([Ions score 114](http://gary/mascot/cgi/peptide_view.pl?file=../data/20130722/F001265.dat&query=685&hit=1&index=gene_51|putative&px=1&section=5&ave_thresh=3&_ignoreionsscorebelow=20&report=0&_sigthreshold=0.05&_msresflags=1089&_msresflags2=2&percolate=-1&percolate_rt=0))

**272 - 287 536.2667 1605.7782 1605.7747 2 0 R.VEAAGNGGGQDIIYSR.R**  ([Ions score 56](http://gary/mascot/cgi/peptide_view.pl?file=../data/20130722/F001265.dat&query=686&hit=1&index=gene_51|putative&px=1&section=5&ave_thresh=3&_ignoreionsscorebelow=20&report=0&_sigthreshold=0.05&_msresflags=1089&_msresflags2=2&percolate=-1&percolate_rt=0))

**272 - 287 804.3865 1606.7584 1606.7587 0 0 R.VEAAGNGGGQDIIYSR.R**  Deamidated (NQ) ([Ions score 119](http://gary/mascot/cgi/peptide_view.pl?file=../data/20130722/F001265.dat&query=688&hit=1&index=gene_51|putative&px=1&section=5&ave_thresh=3&_ignoreionsscorebelow=20&report=0&_sigthreshold=0.05&_msresflags=1089&_msresflags2=2&percolate=-1&percolate_rt=0))

**272 - 288 588.3002 1761.8789 1761.8758 2 1 R.VEAAGNGGGQDIIYSRR.S**  ([Ions score 29](http://gary/mascot/cgi/peptide_view.pl?file=../data/20130722/F001265.dat&query=716&hit=1&index=gene_51|putative&px=1&section=5&ave_thresh=3&_ignoreionsscorebelow=20&report=0&_sigthreshold=0.05&_msresflags=1089&_msresflags2=2&percolate=-1&percolate_rt=0))

**272 - 288 588.6276 1762.8610 1762.8598 1 1 R.VEAAGNGGGQDIIYSRR.S**  Deamidated (NQ) ([Ions score 41](http://gary/mascot/cgi/peptide_view.pl?file=../data/20130722/F001265.dat&query=717&hit=1&index=gene_51|putative&px=1&section=5&ave_thresh=3&_ignoreionsscorebelow=20&report=0&_sigthreshold=0.05&_msresflags=1089&_msresflags2=2&percolate=-1&percolate_rt=0))

**288 - 322 922.9639 3687.8266 3687.8394 -3 1 R.RSDIIHPLGFQFTSASVAGTSATQAELATAANWNR.V**  ([Ions score 57](http://gary/mascot/cgi/peptide_view.pl?file=../data/20130722/F001265.dat&query=1014&hit=1&index=gene_51|putative&px=1&section=5&ave_thresh=3&_ignoreionsscorebelow=20&report=0&_sigthreshold=0.05&_msresflags=1089&_msresflags2=2&percolate=-1&percolate_rt=0))

**288 - 322 922.9643 3687.8281 3687.8394 -3 1 R.RSDIIHPLGFQFTSASVAGTSATQAELATAANWNR.V**  ([Ions score 49](http://gary/mascot/cgi/peptide_view.pl?file=../data/20130722/F001265.dat&query=1015&hit=1&index=gene_51|putative&px=1&section=5&ave_thresh=3&_ignoreionsscorebelow=20&report=0&_sigthreshold=0.05&_msresflags=1089&_msresflags2=2&percolate=-1&percolate_rt=0))

**288 - 322 922.9646 3687.8293 3687.8394 -3 1 R.RSDIIHPLGFQFTSASVAGTSATQAELATAANWNR.V**  ([Ions score 66](http://gary/mascot/cgi/peptide_view.pl?file=../data/20130722/F001265.dat&query=1016&hit=1&index=gene_51|putative&px=1&section=5&ave_thresh=3&_ignoreionsscorebelow=20&report=0&_sigthreshold=0.05&_msresflags=1089&_msresflags2=2&percolate=-1&percolate_rt=0))

**288 - 322 922.9646 3687.8293 3687.8394 -3 1 R.RSDIIHPLGFQFTSASVAGTSATQAELATAANWNR.V**  ([Ions score 70](http://gary/mascot/cgi/peptide_view.pl?file=../data/20130722/F001265.dat&query=1017&hit=1&index=gene_51|putative&px=1&section=5&ave_thresh=3&_ignoreionsscorebelow=20&report=0&_sigthreshold=0.05&_msresflags=1089&_msresflags2=2&percolate=-1&percolate_rt=0))

**288 - 322 922.9655 3687.8330 3687.8394 -2 1 R.RSDIIHPLGFQFTSASVAGTSATQAELATAANWNR.V**  ([Ions score 78](http://gary/mascot/cgi/peptide_view.pl?file=../data/20130722/F001265.dat&query=1018&hit=1&index=gene_51|putative&px=1&section=5&ave_thresh=3&_ignoreionsscorebelow=20&report=0&_sigthreshold=0.05&_msresflags=1089&_msresflags2=2&percolate=-1&percolate_rt=0))

**288 - 322 922.9659 3687.8347 3687.8394 -1 1 R.RSDIIHPLGFQFTSASVAGTSATQAELATAANWNR.V**  ([Ions score 44](http://gary/mascot/cgi/peptide_view.pl?file=../data/20130722/F001265.dat&query=1019&hit=1&index=gene_51|putative&px=1&section=5&ave_thresh=3&_ignoreionsscorebelow=20&report=0&_sigthreshold=0.05&_msresflags=1089&_msresflags2=2&percolate=-1&percolate_rt=0))

**288 - 322 922.9661 3687.8351 3687.8394 -1 1 R.RSDIIHPLGFQFTSASVAGTSATQAELATAANWNR.V**  ([Ions score 69](http://gary/mascot/cgi/peptide_view.pl?file=../data/20130722/F001265.dat&query=1020&hit=1&index=gene_51|putative&px=1&section=5&ave_thresh=3&_ignoreionsscorebelow=20&report=0&_sigthreshold=0.05&_msresflags=1089&_msresflags2=2&percolate=-1&percolate_rt=0))

**288 - 322 922.9663 3687.8361 3687.8394 -1 1 R.RSDIIHPLGFQFTSASVAGTSATQAELATAANWNR.V**  ([Ions score 101](http://gary/mascot/cgi/peptide_view.pl?file=../data/20130722/F001265.dat&query=1021&hit=1&index=gene_51|putative&px=1&section=5&ave_thresh=3&_ignoreionsscorebelow=20&report=0&_sigthreshold=0.05&_msresflags=1089&_msresflags2=2&percolate=-1&percolate_rt=0))

**288 - 322 922.9665 3687.8369 3687.8394 -1 1 R.RSDIIHPLGFQFTSASVAGTSATQAELATAANWNR.V**  ([Ions score 66](http://gary/mascot/cgi/peptide_view.pl?file=../data/20130722/F001265.dat&query=1022&hit=1&index=gene_51|putative&px=1&section=5&ave_thresh=3&_ignoreionsscorebelow=20&report=0&_sigthreshold=0.05&_msresflags=1089&_msresflags2=2&percolate=-1&percolate_rt=0))

**288 - 322 922.9665 3687.8371 3687.8394 -1 1 R.RSDIIHPLGFQFTSASVAGTSATQAELATAANWNR.V**  ([Ions score 72](http://gary/mascot/cgi/peptide_view.pl?file=../data/20130722/F001265.dat&query=1023&hit=1&index=gene_51|putative&px=1&section=5&ave_thresh=3&_ignoreionsscorebelow=20&report=0&_sigthreshold=0.05&_msresflags=1089&_msresflags2=2&percolate=-1&percolate_rt=0))

**288 - 322 922.9668 3687.8381 3687.8394 0 1 R.RSDIIHPLGFQFTSASVAGTSATQAELATAANWNR.V**  ([Ions score 74](http://gary/mascot/cgi/peptide_view.pl?file=../data/20130722/F001265.dat&query=1024&hit=1&index=gene_51|putative&px=1&section=5&ave_thresh=3&_ignoreionsscorebelow=20&report=0&_sigthreshold=0.05&_msresflags=1089&_msresflags2=2&percolate=-1&percolate_rt=0))

**288 - 322 922.9681 3687.8432 3687.8394 1 1 R.RSDIIHPLGFQFTSASVAGTSATQAELATAANWNR.V**  ([Ions score 41](http://gary/mascot/cgi/peptide_view.pl?file=../data/20130722/F001265.dat&query=1025&hit=1&index=gene_51|putative&px=1&section=5&ave_thresh=3&_ignoreionsscorebelow=20&report=0&_sigthreshold=0.05&_msresflags=1089&_msresflags2=2&percolate=-1&percolate_rt=0))

**289 - 322 1178.2566 3531.7479 3531.7383 3 0 R.SDIIHPLGFQFTSASVAGTSATQAELATAANWNR.V**  ([Ions score 83](http://gary/mascot/cgi/peptide_view.pl?file=../data/20130722/F001265.dat&query=1012&hit=1&index=gene_51|putative&px=1&section=5&ave_thresh=3&_ignoreionsscorebelow=20&report=0&_sigthreshold=0.05&_msresflags=1089&_msresflags2=2&percolate=-1&percolate_rt=0))

**323 - 327 347.6974 693.3803 693.3810 -1 1 R.VYERK.N**  ([Ions score 25](http://gary/mascot/cgi/peptide_view.pl?file=../data/20130722/F001265.dat&query=9&hit=1&index=gene_51|putative&px=1&section=5&ave_thresh=3&_ignoreionsscorebelow=20&report=0&_sigthreshold=0.05&_msresflags=1089&_msresflags2=2&percolate=-1&percolate_rt=0))

**327 - 335 486.3165 970.6185 970.6175 1 1 R.KNVSLAVLK.S**  ([Ions score 32](http://gary/mascot/cgi/peptide_view.pl?file=../data/20130722/F001265.dat&query=162&hit=1&index=gene_51|putative&px=1&section=5&ave_thresh=3&_ignoreionsscorebelow=20&report=0&_sigthreshold=0.05&_msresflags=1089&_msresflags2=2&percolate=-1&percolate_rt=0))

**327 - 335 324.5472 970.6199 970.6175 2 1 R.KNVSLAVLK.S**  ([Ions score 31](http://gary/mascot/cgi/peptide_view.pl?file=../data/20130722/F001265.dat&query=163&hit=1&index=gene_51|putative&px=1&section=5&ave_thresh=3&_ignoreionsscorebelow=20&report=0&_sigthreshold=0.05&_msresflags=1089&_msresflags2=2&percolate=-1&percolate_rt=0))

**327 - 335 486.8077 971.6009 971.6015 -1 1 R.KNVSLAVLK.S**  Deamidated (NQ) ([Ions score 46](http://gary/mascot/cgi/peptide_view.pl?file=../data/20130722/F001265.dat&query=164&hit=1&index=gene_51|putative&px=1&section=5&ave_thresh=3&_ignoreionsscorebelow=20&report=0&_sigthreshold=0.05&_msresflags=1089&_msresflags2=2&percolate=-1&percolate_rt=0))

**328 - 335 422.2683 842.5220 842.5225 -1 0 K.NVSLAVLK.S**  ([Ions score 35](http://gary/mascot/cgi/peptide_view.pl?file=../data/20130722/F001265.dat&query=84&hit=1&index=gene_51|putative&px=1&section=5&ave_thresh=3&_ignoreionsscorebelow=20&report=0&_sigthreshold=0.05&_msresflags=1089&_msresflags2=2&percolate=-1&percolate_rt=0))

**328 - 335 422.2683 842.5221 842.5225 0 0 K.NVSLAVLK.S**  ([Ions score 20](http://gary/mascot/cgi/peptide_view.pl?file=../data/20130722/F001265.dat&query=85&hit=1&index=gene_51|putative&px=1&section=5&ave_thresh=3&_ignoreionsscorebelow=20&report=0&_sigthreshold=0.05&_msresflags=1089&_msresflags2=2&percolate=-1&percolate_rt=0))

**328 - 335 422.2684 842.5222 842.5225 0 0 K.NVSLAVLK.S**  ([Ions score 62](http://gary/mascot/cgi/peptide_view.pl?file=../data/20130722/F001265.dat&query=86&hit=1&index=gene_51|putative&px=1&section=5&ave_thresh=3&_ignoreionsscorebelow=20&report=0&_sigthreshold=0.05&_msresflags=1089&_msresflags2=2&percolate=-1&percolate_rt=0))

**328 - 335 422.2684 842.5222 842.5225 0 0 K.NVSLAVLK.S**  ([Ions score 24](http://gary/mascot/cgi/peptide_view.pl?file=../data/20130722/F001265.dat&query=87&hit=1&index=gene_51|putative&px=1&section=5&ave_thresh=3&_ignoreionsscorebelow=20&report=0&_sigthreshold=0.05&_msresflags=1089&_msresflags2=2&percolate=-1&percolate_rt=0))

**328 - 335 422.2684 842.5222 842.5225 0 0 K.NVSLAVLK.S**  ([Ions score 32](http://gary/mascot/cgi/peptide_view.pl?file=../data/20130722/F001265.dat&query=88&hit=1&index=gene_51|putative&px=1&section=5&ave_thresh=3&_ignoreionsscorebelow=20&report=0&_sigthreshold=0.05&_msresflags=1089&_msresflags2=2&percolate=-1&percolate_rt=0))

**328 - 335 422.2685 842.5225 842.5225 0 0 K.NVSLAVLK.S**  ([Ions score 27](http://gary/mascot/cgi/peptide_view.pl?file=../data/20130722/F001265.dat&query=90&hit=1&index=gene_51|putative&px=1&section=5&ave_thresh=3&_ignoreionsscorebelow=20&report=0&_sigthreshold=0.05&_msresflags=1089&_msresflags2=2&percolate=-1&percolate_rt=0))

**328 - 335 422.2687 842.5229 842.5225 0 0 K.NVSLAVLK.S**  ([Ions score 46](http://gary/mascot/cgi/peptide_view.pl?file=../data/20130722/F001265.dat&query=92&hit=1&index=gene_51|putative&px=1&section=5&ave_thresh=3&_ignoreionsscorebelow=20&report=0&_sigthreshold=0.05&_msresflags=1089&_msresflags2=2&percolate=-1&percolate_rt=0))

**328 - 335 422.7600 843.5053 843.5065 -1 0 K.NVSLAVLK.S**  Deamidated (NQ) ([Ions score 39](http://gary/mascot/cgi/peptide_view.pl?file=../data/20130722/F001265.dat&query=96&hit=1&index=gene_51|putative&px=1&section=5&ave_thresh=3&_ignoreionsscorebelow=20&report=0&_sigthreshold=0.05&_msresflags=1089&_msresflags2=2&percolate=-1&percolate_rt=0))

**328 - 338 551.3174 1100.6203 1100.6190 1 1 K.NVSLAVLKSNG.-**  ([Ions score 50](http://gary/mascot/cgi/peptide_view.pl?file=../data/20130722/F001265.dat&query=273&hit=1&index=gene_51|putative&px=1&section=5&ave_thresh=3&_ignoreionsscorebelow=20&report=0&_sigthreshold=0.05&_msresflags=1089&_msresflags2=2&percolate=-1&percolate_rt=0))

**2.**[gene_59|putative](http://10.139.25.109/mascot/cgi/protein_view.pl?file=../data/20100316/F010066.dat&hit=gene_59|putative&px=1&ave_thresh=1&_sigthreshold=0.05&_server_mudpit_switch=1e-009&_ignoreionsscorebelow=20) tail protein|[Serratia phage Eta] **Mass:** 40531    **Score:** 2376   **Queries**

**matched:** 49(49) **emPAI:** 13.41; Sequence Coverage: **53%**; Matched peptides shown in **Bold Red**

**1** **MSIQPFKGAN TANFYIPEVT AGVTPASPAW YPLRNTGGVP ALTRDALVSN ELDGSREVSS IRTGNK**QVSG EFAIELSAQS QDELLAGAMT SSWVAGSTVT

**101** GLTIDVSASG KTFTRSAGDF TTTVEVGDLI AFPDLAGDNA KPFIVTAVSA LVVTGAGIPH ALTDETDTTT DLIIGDK**LET GNLCKTYSVL TVFTGKCGTV**

**201 DAYLLTK**GVE FSGFSIEQAV NAMVTGSLPF IGLSQEVLSA LPAGSTFPYS YDAEPFSSVD VSAFNGTALL K**LIDTFTITN DNATSAQFEL GNDSVAFVER**

**301 GRASNTFSLA GKLYDMTLLN LFLNETKVEM TSILSGVTGA MSFTLKRADL TAATPEVGGP ESVTLTIEGQ ATGNNTLSSI VIQR**IAYA

**Start - End Observed Mr(expt) Mr(calc) ppm Miss Sequence**

**1 - 7 433.7256 865.4366 865.4368 0 0 -.MSIQPFK.G**  Oxidation (M) ([Ions score 29](http://gary/mascot/cgi/peptide_view.pl?file=../data/20130722/F001266.dat&query=114&hit=1&index=gene_59|putative&px=1&section=5&ave_thresh=2&_ignoreionsscorebelow=20&report=0&_sigthreshold=0.05&_msresflags=1089&_msresflags2=2&percolate=-1&percolate_rt=0))

**2 - 7 360.2077 718.4008 718.4014 -1 0 M.SIQPFK.G**  ([Ions score 39](http://gary/mascot/cgi/peptide_view.pl?file=../data/20130722/F001266.dat&query=23&hit=1&index=gene_59|putative&px=1&section=5&ave_thresh=2&_ignoreionsscorebelow=20&report=0&_sigthreshold=0.05&_msresflags=1089&_msresflags2=2&percolate=-1&percolate_rt=0))

**2 - 34 1188.6193 3562.8360 3562.8249 3 1 M.SIQPFKGANTANFYIPEVTAGVTPASPAWYPLR.N**  ([Ions score 68](http://gary/mascot/cgi/peptide_view.pl?file=../data/20130722/F001266.dat&query=930&hit=1&index=gene_59|putative&px=1&section=5&ave_thresh=2&_ignoreionsscorebelow=20&report=0&_sigthreshold=0.05&_msresflags=1089&_msresflags2=2&percolate=-1&percolate_rt=0))

**2 - 44 1133.3423 4529.3401 4529.3496 -2 2 M.SIQPFKGANTANFYIPEVTAGVTPASPAWYPLRNTGGVPALTR.D**([Ions score 86](http://gary/mascot/cgi/peptide_view.pl?file=../data/20130722/F001266.dat&query=958&hit=1&index=gene_59|putative&px=1&section=5&ave_thresh=2&_ignoreionsscorebelow=20&report=0&_sigthreshold=0.05&_msresflags=1089&_msresflags2=2&percolate=-1&percolate_rt=0))

**8 - 34 955.1514 2862.4323 2862.4341 -1 0 K.GANTANFYIPEVTAGVTPASPAWYPLR.N**  ([Ions score 58](http://gary/mascot/cgi/peptide_view.pl?file=../data/20130722/F001266.dat&query=894&hit=1&index=gene_59|putative&px=1&section=5&ave_thresh=2&_ignoreionsscorebelow=20&report=0&_sigthreshold=0.05&_msresflags=1089&_msresflags2=2&percolate=-1&percolate_rt=0))

**8 - 44 1277.3281 3828.9626 3828.9588 1 1 K.GANTANFYIPEVTAGVTPASPAWYPLRNTGGVPALTR.D**  ([Ions score 126](http://gary/mascot/cgi/peptide_view.pl?file=../data/20130722/F001266.dat&query=948&hit=1&index=gene_59|putative&px=1&section=5&ave_thresh=2&_ignoreionsscorebelow=20&report=0&_sigthreshold=0.05&_msresflags=1089&_msresflags2=2&percolate=-1&percolate_rt=0))

**35 - 44 493.2745 984.5344 984.5352 -1 0 R.NTGGVPALTR.D**  ([Ions score 37](http://gary/mascot/cgi/peptide_view.pl?file=../data/20130722/F001266.dat&query=193&hit=1&index=gene_59|putative&px=1&section=5&ave_thresh=2&_ignoreionsscorebelow=20&report=0&_sigthreshold=0.05&_msresflags=1089&_msresflags2=2&percolate=-1&percolate_rt=0))

**35 - 56 748.0558 2241.1457 2241.1349 5 1 R.NTGGVPALTRDALVSNELDGSR.E**  ([Ions score 50](http://gary/mascot/cgi/peptide_view.pl?file=../data/20130722/F001266.dat&query=798&hit=1&index=gene_59|putative&px=1&section=5&ave_thresh=2&_ignoreionsscorebelow=20&report=0&_sigthreshold=0.05&_msresflags=1089&_msresflags2=2&percolate=-1&percolate_rt=0))

**45 - 56 638.3133 1274.6120 1274.6102 1 0 R.DALVSNELDGSR.E**  ([Ions score 73](http://gary/mascot/cgi/peptide_view.pl?file=../data/20130722/F001266.dat&query=516&hit=1&index=gene_59|putative&px=1&section=5&ave_thresh=2&_ignoreionsscorebelow=20&report=0&_sigthreshold=0.05&_msresflags=1089&_msresflags2=2&percolate=-1&percolate_rt=0))

**45 - 56 638.8054 1275.5962 1275.5943 2 0 R.DALVSNELDGSR.E**  Deamidated (NQ) ([Ions score 72](http://gary/mascot/cgi/peptide_view.pl?file=../data/20130722/F001266.dat&query=517&hit=1&index=gene_59|putative&px=1&section=5&ave_thresh=2&_ignoreionsscorebelow=20&report=0&_sigthreshold=0.05&_msresflags=1089&_msresflags2=2&percolate=-1&percolate_rt=0))

**45 - 62 973.9921 1945.9697 1945.9705 0 1 R.DALVSNELDGSREVSSIR.T**  ([Ions score 68](http://gary/mascot/cgi/peptide_view.pl?file=../data/20130722/F001266.dat&query=753&hit=1&index=gene_59|putative&px=1&section=5&ave_thresh=2&_ignoreionsscorebelow=20&report=0&_sigthreshold=0.05&_msresflags=1089&_msresflags2=2&percolate=-1&percolate_rt=0))

**45 - 62 649.6644 1945.9713 1945.9705 0 1 R.DALVSNELDGSREVSSIR.T**  ([Ions score 67](http://gary/mascot/cgi/peptide_view.pl?file=../data/20130722/F001266.dat&query=754&hit=1&index=gene_59|putative&px=1&section=5&ave_thresh=2&_ignoreionsscorebelow=20&report=0&_sigthreshold=0.05&_msresflags=1089&_msresflags2=2&percolate=-1&percolate_rt=0))

**45 - 62 649.6651 1945.9735 1945.9705 2 1 R.DALVSNELDGSREVSSIR.T**  ([Ions score 56](http://gary/mascot/cgi/peptide_view.pl?file=../data/20130722/F001266.dat&query=755&hit=1&index=gene_59|putative&px=1&section=5&ave_thresh=2&_ignoreionsscorebelow=20&report=0&_sigthreshold=0.05&_msresflags=1089&_msresflags2=2&percolate=-1&percolate_rt=0))

**45 - 62 649.9947 1946.9622 1946.9545 4 1 R.DALVSNELDGSREVSSIR.T**  Deamidated (NQ) ([Ions score 47](http://gary/mascot/cgi/peptide_view.pl?file=../data/20130722/F001266.dat&query=756&hit=1&index=gene_59|putative&px=1&section=5&ave_thresh=2&_ignoreionsscorebelow=20&report=0&_sigthreshold=0.05&_msresflags=1089&_msresflags2=2&percolate=-1&percolate_rt=0))

**45 - 66 783.0653 2346.1741 2346.1775 -1 2 R.DALVSNELDGSREVSSIRTGNK.Q**  ([Ions score 82](http://gary/mascot/cgi/peptide_view.pl?file=../data/20130722/F001266.dat&query=820&hit=1&index=gene_59|putative&px=1&section=5&ave_thresh=2&_ignoreionsscorebelow=20&report=0&_sigthreshold=0.05&_msresflags=1089&_msresflags2=2&percolate=-1&percolate_rt=0))

**45 - 66 783.0664 2346.1772 2346.1775 0 2 R.DALVSNELDGSREVSSIRTGNK.Q**  ([Ions score 56](http://gary/mascot/cgi/peptide_view.pl?file=../data/20130722/F001266.dat&query=822&hit=1&index=gene_59|putative&px=1&section=5&ave_thresh=2&_ignoreionsscorebelow=20&report=0&_sigthreshold=0.05&_msresflags=1089&_msresflags2=2&percolate=-1&percolate_rt=0))

**45 - 66 783.3961 2347.1665 2347.1615 2 2 R.DALVSNELDGSREVSSIRTGNK.Q**  Deamidated (NQ) ([Ions score 51](http://gary/mascot/cgi/peptide_view.pl?file=../data/20130722/F001266.dat&query=824&hit=1&index=gene_59|putative&px=1&section=5&ave_thresh=2&_ignoreionsscorebelow=20&report=0&_sigthreshold=0.05&_msresflags=1089&_msresflags2=2&percolate=-1&percolate_rt=0))

**57 - 62 345.6927 689.3709 689.3708 0 0 R.EVSSIR.T**  ([Ions score 30](http://gary/mascot/cgi/peptide_view.pl?file=../data/20130722/F001266.dat&query=14&hit=1&index=gene_59|putative&px=1&section=5&ave_thresh=2&_ignoreionsscorebelow=20&report=0&_sigthreshold=0.05&_msresflags=1089&_msresflags2=2&percolate=-1&percolate_rt=0))

**57 - 66 364.1999 1089.5778 1089.5778 0 1 R.EVSSIRTGNK.Q**  ([Ions score 21](http://gary/mascot/cgi/peptide_view.pl?file=../data/20130722/F001266.dat&query=288&hit=1&index=gene_59|putative&px=1&section=5&ave_thresh=2&_ignoreionsscorebelow=20&report=0&_sigthreshold=0.05&_msresflags=1089&_msresflags2=2&percolate=-1&percolate_rt=0))

**57 - 66 545.7962 1089.5778 1089.5778 0 1 R.EVSSIRTGNK.Q**  ([Ions score 51](http://gary/mascot/cgi/peptide_view.pl?file=../data/20130722/F001266.dat&query=289&hit=1&index=gene_59|putative&px=1&section=5&ave_thresh=2&_ignoreionsscorebelow=20&report=0&_sigthreshold=0.05&_msresflags=1089&_msresflags2=2&percolate=-1&percolate_rt=0))

**186 - 196 608.3339 1214.6532 1214.6547 -1 0 K.TYSVLTVFTGK.C**  ([Ions score 50](http://gary/mascot/cgi/peptide_view.pl?file=../data/20130722/F001266.dat&query=434&hit=1&index=gene_59|putative&px=1&section=5&ave_thresh=2&_ignoreionsscorebelow=20&report=0&_sigthreshold=0.05&_msresflags=1089&_msresflags2=2&percolate=-1&percolate_rt=0))

**186 - 196 608.3339 1214.6533 1214.6547 -1 0 K.TYSVLTVFTGK.C**  ([Ions score 64](http://gary/mascot/cgi/peptide_view.pl?file=../data/20130722/F001266.dat&query=435&hit=1&index=gene_59|putative&px=1&section=5&ave_thresh=2&_ignoreionsscorebelow=20&report=0&_sigthreshold=0.05&_msresflags=1089&_msresflags2=2&percolate=-1&percolate_rt=0))

**186 - 196 608.3341 1214.6537 1214.6547 -1 0 K.TYSVLTVFTGK.C**  ([Ions score 40](http://gary/mascot/cgi/peptide_view.pl?file=../data/20130722/F001266.dat&query=436&hit=1&index=gene_59|putative&px=1&section=5&ave_thresh=2&_ignoreionsscorebelow=20&report=0&_sigthreshold=0.05&_msresflags=1089&_msresflags2=2&percolate=-1&percolate_rt=0))

**186 - 196 608.3348 1214.6551 1214.6547 0 0 K.TYSVLTVFTGK.C**  ([Ions score 89](http://gary/mascot/cgi/peptide_view.pl?file=../data/20130722/F001266.dat&query=437&hit=1&index=gene_59|putative&px=1&section=5&ave_thresh=2&_ignoreionsscorebelow=20&report=0&_sigthreshold=0.05&_msresflags=1089&_msresflags2=2&percolate=-1&percolate_rt=0))

**186 - 196 608.3363 1214.6580 1214.6547 3 0 K.TYSVLTVFTGK.C**  ([Ions score 51](http://gary/mascot/cgi/peptide_view.pl?file=../data/20130722/F001266.dat&query=438&hit=1&index=gene_59|putative&px=1&section=5&ave_thresh=2&_ignoreionsscorebelow=20&report=0&_sigthreshold=0.05&_msresflags=1089&_msresflags2=2&percolate=-1&percolate_rt=0))

**186 - 196 608.3364 1214.6583 1214.6547 3 0 K.TYSVLTVFTGK.C**  ([Ions score 51](http://gary/mascot/cgi/peptide_view.pl?file=../data/20130722/F001266.dat&query=439&hit=1&index=gene_59|putative&px=1&section=5&ave_thresh=2&_ignoreionsscorebelow=20&report=0&_sigthreshold=0.05&_msresflags=1089&_msresflags2=2&percolate=-1&percolate_rt=0))

**186 - 196 608.3369 1214.6593 1214.6547 4 0 K.TYSVLTVFTGK.C**  ([Ions score 51](http://gary/mascot/cgi/peptide_view.pl?file=../data/20130722/F001266.dat&query=440&hit=1&index=gene_59|putative&px=1&section=5&ave_thresh=2&_ignoreionsscorebelow=20&report=0&_sigthreshold=0.05&_msresflags=1089&_msresflags2=2&percolate=-1&percolate_rt=0))

**197 - 207 620.8150 1239.6154 1239.6169 -1 0 K.CGTVDAYLLTK.G**  ([Ions score 53](http://gary/mascot/cgi/peptide_view.pl?file=../data/20130722/F001266.dat&query=473&hit=1&index=gene_59|putative&px=1&section=5&ave_thresh=2&_ignoreionsscorebelow=20&report=0&_sigthreshold=0.05&_msresflags=1089&_msresflags2=2&percolate=-1&percolate_rt=0))

**197 - 207 620.8154 1239.6163 1239.6169 -1 0 K.CGTVDAYLLTK.G**  ([Ions score 67](http://gary/mascot/cgi/peptide_view.pl?file=../data/20130722/F001266.dat&query=474&hit=1&index=gene_59|putative&px=1&section=5&ave_thresh=2&_ignoreionsscorebelow=20&report=0&_sigthreshold=0.05&_msresflags=1089&_msresflags2=2&percolate=-1&percolate_rt=0))

**197 - 207 620.8158 1239.6170 1239.6169 0 0 K.CGTVDAYLLTK.G**  ([Ions score 23](http://gary/mascot/cgi/peptide_view.pl?file=../data/20130722/F001266.dat&query=475&hit=1&index=gene_59|putative&px=1&section=5&ave_thresh=2&_ignoreionsscorebelow=20&report=0&_sigthreshold=0.05&_msresflags=1089&_msresflags2=2&percolate=-1&percolate_rt=0))

**197 - 207 620.8163 1239.6180 1239.6169 1 0 K.CGTVDAYLLTK.G**  ([Ions score 48](http://gary/mascot/cgi/peptide_view.pl?file=../data/20130722/F001266.dat&query=476&hit=1&index=gene_59|putative&px=1&section=5&ave_thresh=2&_ignoreionsscorebelow=20&report=0&_sigthreshold=0.05&_msresflags=1089&_msresflags2=2&percolate=-1&percolate_rt=0))

**197 - 207 620.8171 1239.6197 1239.6169 2 0 K.CGTVDAYLLTK.G**  ([Ions score 46](http://gary/mascot/cgi/peptide_view.pl?file=../data/20130722/F001266.dat&query=477&hit=1&index=gene_59|putative&px=1&section=5&ave_thresh=2&_ignoreionsscorebelow=20&report=0&_sigthreshold=0.05&_msresflags=1089&_msresflags2=2&percolate=-1&percolate_rt=0))

**197 - 207 620.8192 1239.6237 1239.6169 6 0 K.CGTVDAYLLTK.G**  ([Ions score 50](http://gary/mascot/cgi/peptide_view.pl?file=../data/20130722/F001266.dat&query=478&hit=1&index=gene_59|putative&px=1&section=5&ave_thresh=2&_ignoreionsscorebelow=20&report=0&_sigthreshold=0.05&_msresflags=1089&_msresflags2=2&percolate=-1&percolate_rt=0))

**272 - 300 1063.5142 3187.5207 3187.5310 -3 0 K.LIDTFTITNDNATSAQFELGNDSVAFVER.G**  ([Ions score 57](http://gary/mascot/cgi/peptide_view.pl?file=../data/20130722/F001266.dat&query=918&hit=1&index=gene_59|putative&px=1&section=5&ave_thresh=2&_ignoreionsscorebelow=20&report=0&_sigthreshold=0.05&_msresflags=1089&_msresflags2=2&percolate=-1&percolate_rt=0))

**272 - 300 1063.5166 3187.5280 3187.5310 -1 0 K.LIDTFTITNDNATSAQFELGNDSVAFVER.G**  ([Ions score 80](http://gary/mascot/cgi/peptide_view.pl?file=../data/20130722/F001266.dat&query=919&hit=1&index=gene_59|putative&px=1&section=5&ave_thresh=2&_ignoreionsscorebelow=20&report=0&_sigthreshold=0.05&_msresflags=1089&_msresflags2=2&percolate=-1&percolate_rt=0))

**272 - 300 1063.5179 3187.5320 3187.5310 0 0 K.LIDTFTITNDNATSAQFELGNDSVAFVER.G**  ([Ions score 67](http://gary/mascot/cgi/peptide_view.pl?file=../data/20130722/F001266.dat&query=920&hit=1&index=gene_59|putative&px=1&section=5&ave_thresh=2&_ignoreionsscorebelow=20&report=0&_sigthreshold=0.05&_msresflags=1089&_msresflags2=2&percolate=-1&percolate_rt=0))

**301 - 312 604.8235 1207.6325 1207.6309 1 1 R.GRASNTFSLAGK.L**  ([Ions score 47](http://gary/mascot/cgi/peptide_view.pl?file=../data/20130722/F001266.dat&query=429&hit=1&index=gene_59|putative&px=1&section=5&ave_thresh=2&_ignoreionsscorebelow=20&report=0&_sigthreshold=0.05&_msresflags=1089&_msresflags2=2&percolate=-1&percolate_rt=0))

**303 - 312 498.2617 994.5088 994.5083 0 0 R.ASNTFSLAGK.L**  ([Ions score 69](http://gary/mascot/cgi/peptide_view.pl?file=../data/20130722/F001266.dat&query=199&hit=1&index=gene_59|putative&px=1&section=5&ave_thresh=2&_ignoreionsscorebelow=20&report=0&_sigthreshold=0.05&_msresflags=1089&_msresflags2=2&percolate=-1&percolate_rt=0))

**303 - 312 498.7509 995.4873 995.4924 -5 0 R.ASNTFSLAGK.L**  Deamidated (NQ) ([Ions score 64](http://gary/mascot/cgi/peptide_view.pl?file=../data/20130722/F001266.dat&query=200&hit=1&index=gene_59|putative&px=1&section=5&ave_thresh=2&_ignoreionsscorebelow=20&report=0&_sigthreshold=0.05&_msresflags=1089&_msresflags2=2&percolate=-1&percolate_rt=0))

**313 - 327 914.4796 1826.9445 1826.9488 -2 0 K.LYDMTLLNLFLNETK.V**  ([Ions score 97](http://gary/mascot/cgi/peptide_view.pl?file=../data/20130722/F001266.dat&query=715&hit=1&index=gene_59|putative&px=1&section=5&ave_thresh=2&_ignoreionsscorebelow=20&report=0&_sigthreshold=0.05&_msresflags=1089&_msresflags2=2&percolate=-1&percolate_rt=0))

**313 - 327 609.9905 1826.9498 1826.9488 1 0 K.LYDMTLLNLFLNETK.V**  ([Ions score 66](http://gary/mascot/cgi/peptide_view.pl?file=../data/20130722/F001266.dat&query=716&hit=1&index=gene_59|putative&px=1&section=5&ave_thresh=2&_ignoreionsscorebelow=20&report=0&_sigthreshold=0.05&_msresflags=1089&_msresflags2=2&percolate=-1&percolate_rt=0))

**313 - 327 615.3204 1842.9395 1842.9437 -2 0 K.LYDMTLLNLFLNETK.V**  Oxidation (M) ([Ions score 53](http://gary/mascot/cgi/peptide_view.pl?file=../data/20130722/F001266.dat&query=719&hit=1&index=gene_59|putative&px=1&section=5&ave_thresh=2&_ignoreionsscorebelow=20&report=0&_sigthreshold=0.05&_msresflags=1089&_msresflags2=2&percolate=-1&percolate_rt=0))

**328 - 346 994.5081 1987.0016 1987.0006 0 0 K.VEMTSILSGVTGAMSFTLK.R**  Oxidation (M) ([Ions score 125](http://gary/mascot/cgi/peptide_view.pl?file=../data/20130722/F001266.dat&query=767&hit=1&index=gene_59|putative&px=1&section=5&ave_thresh=2&_ignoreionsscorebelow=20&report=0&_sigthreshold=0.05&_msresflags=1089&_msresflags2=2&percolate=-1&percolate_rt=0))

**328 - 346 668.6717 2002.9934 2002.9955 -1 0 K.VEMTSILSGVTGAMSFTLK.R**  2 Oxidation (M) ([Ions score 88](http://gary/mascot/cgi/peptide_view.pl?file=../data/20130722/F001266.dat&query=772&hit=1&index=gene_59|putative&px=1&section=5&ave_thresh=2&_ignoreionsscorebelow=20&report=0&_sigthreshold=0.05&_msresflags=1089&_msresflags2=2&percolate=-1&percolate_rt=0))

**328 - 346 1002.5060 2002.9975 2002.9955 1 0 K.VEMTSILSGVTGAMSFTLK.R**  2 Oxidation (M) ([Ions score 113](http://gary/mascot/cgi/peptide_view.pl?file=../data/20130722/F001266.dat&query=773&hit=1&index=gene_59|putative&px=1&section=5&ave_thresh=2&_ignoreionsscorebelow=20&report=0&_sigthreshold=0.05&_msresflags=1089&_msresflags2=2&percolate=-1&percolate_rt=0))

**347 - 384 967.5064 3865.9965 3866.0021 -1 1 K.RADLTAATPEVGGPESVTLTIEGQATGNNTLSSIVIQR.I**  ([Ions score 63](http://gary/mascot/cgi/peptide_view.pl?file=../data/20130722/F001266.dat&query=953&hit=1&index=gene_59|putative&px=1&section=5&ave_thresh=2&_ignoreionsscorebelow=20&report=0&_sigthreshold=0.05&_msresflags=1089&_msresflags2=2&percolate=-1&percolate_rt=0))

**348 - 384 1237.6427 3709.9063 3709.9010 1 0 R.ADLTAATPEVGGPESVTLTIEGQATGNNTLSSIVIQR.I**  ([Ions score 98](http://gary/mascot/cgi/peptide_view.pl?file=../data/20130722/F001266.dat&query=944&hit=1&index=gene_59|putative&px=1&section=5&ave_thresh=2&_ignoreionsscorebelow=20&report=0&_sigthreshold=0.05&_msresflags=1089&_msresflags2=2&percolate=-1&percolate_rt=0))

**348 - 384 928.4861 3709.9155 3709.9010 4 0 R.ADLTAATPEVGGPESVTLTIEGQATGNNTLSSIVIQR.I**  ([Ions score 36](http://gary/mascot/cgi/peptide_view.pl?file=../data/20130722/F001266.dat&query=945&hit=1&index=gene_59|putative&px=1&section=5&ave_thresh=2&_ignoreionsscorebelow=20&report=0&_sigthreshold=0.05&_msresflags=1089&_msresflags2=2&percolate=-1&percolate_rt=0))

**3.**[gene_43|putative](http://10.139.25.109/mascot/cgi/protein_view.pl?file=../data/20100316/F010066.dat&hit=gene_43|putative&px=1&ave_thresh=1&_sigthreshold=0.05&_server_mudpit_switch=1e-009&_ignoreionsscorebelow=20) head morphogenesis protein|[Serratia phage Eta] **Mass:** 39344    **Score:** 732    **Queries**

**matched:** 17(17)  **emPAI:** 2.95; Sequence Coverage: **42%**; Matched peptides shown in **Bold Red**

**1** MSLTSAFISH QIWLQR**TASH EANLVEPFMK** QMRDEIR**ASV LSFGDDSRTA AR**LNKMLREI EGVLYGITGA WDDKLLEDMK ELAKYEAGWT TRTLTANVDA

**101** SFTTPSPEQV WSAIKFQPLA LDNRPVDFIK LLDNWSDVEV SRLVTGVK**SG FVQGLTTRDI VKQVVGAGGL ADVSERNAK**A VVK**TAINHVS TVAK**DAAYAK

**201** NSDVIEGYEL VVTLDSRTSA ICRGWPQGKV YR**LTDDYQPK PPFHVACRTT TAPVVSSEFD FLDAGAKR**AA R**GADGGTQVD ANTSYYDFLK QQPAWFQDEA**

**301 LGPTRGK**IFR NSGMTPDEFR AASVDGFGRP LTLKEMADMD NK**VADYLAK**I DYKTN

**Start - End Observed Mr(expt) Mr(calc) ppm Miss Sequence**

**17 - 30 530.5920 1588.7541 1588.7555 -1 0 R.TASHEANLVEPFMK.Q**  Oxidation (M) ([Ions score 25](http://gary/mascot/cgi/peptide_view.pl?file=../data/20130722/F001266.dat&query=666&hit=1&index=gene_43|putative&px=1&section=5&ave_thresh=2&_ignoreionsscorebelow=20&report=0&_sigthreshold=0.05&_msresflags=1089&_msresflags2=2&percolate=-1&percolate_rt=0))

**38 - 52 518.2606 1551.7600 1551.7641 -3 1 R.ASVLSFGDDSRTAAR.L**  ([Ions score 29](http://gary/mascot/cgi/peptide_view.pl?file=../data/20130722/F001266.dat&query=656&hit=1&index=gene_43|putative&px=1&section=5&ave_thresh=2&_ignoreionsscorebelow=20&report=0&_sigthreshold=0.05&_msresflags=1089&_msresflags2=2&percolate=-1&percolate_rt=0))

**149 - 158 533.2877 1064.5608 1064.5615 -1 0 K.SGFVQGLTTR.D**  ([Ions score 49](http://gary/mascot/cgi/peptide_view.pl?file=../data/20130722/F001266.dat&query=258&hit=1&index=gene_43|putative&px=1&section=5&ave_thresh=2&_ignoreionsscorebelow=20&report=0&_sigthreshold=0.05&_msresflags=1089&_msresflags2=2&percolate=-1&percolate_rt=0))

**149 - 162 507.6201 1519.8384 1519.8359 2 1 K.SGFVQGLTTRDIVK.Q**  ([Ions score 40](http://gary/mascot/cgi/peptide_view.pl?file=../data/20130722/F001266.dat&query=645&hit=1&index=gene_43|putative&px=1&section=5&ave_thresh=2&_ignoreionsscorebelow=20&report=0&_sigthreshold=0.05&_msresflags=1089&_msresflags2=2&percolate=-1&percolate_rt=0))

**163 - 176 679.3581 1356.7016 1356.6998 1 0 K.QVVGAGGLADVSER.N**  ([Ions score 89](http://gary/mascot/cgi/peptide_view.pl?file=../data/20130722/F001266.dat&query=587&hit=1&index=gene_43|putative&px=1&section=5&ave_thresh=2&_ignoreionsscorebelow=20&report=0&_sigthreshold=0.05&_msresflags=1089&_msresflags2=2&percolate=-1&percolate_rt=0))

**163 - 179 827.4323 1652.8501 1652.8482 1 1 K.QVVGAGGLADVSERNAK.A**  Gln->pyro-Glu(N-term Q)([Ions score 78](http://gary/mascot/cgi/peptide_view.pl?file=../data/20130722/F001266.dat&query=685&hit=1&index=gene_43|putative&px=1&section=5&ave_thresh=2&_ignoreionsscorebelow=20&report=0&_sigthreshold=0.05&_msresflags=1089&_msresflags2=2&percolate=-1&percolate_rt=0))

**163 - 179 557.6324 1669.8755 1669.8747 0 1 K.QVVGAGGLADVSERNAK.A**  ([Ions score 57](http://gary/mascot/cgi/peptide_view.pl?file=../data/20130722/F001266.dat&query=690&hit=1&index=gene_43|putative&px=1&section=5&ave_thresh=2&_ignoreionsscorebelow=20&report=0&_sigthreshold=0.05&_msresflags=1089&_msresflags2=2&percolate=-1&percolate_rt=0))

**184 - 194 380.8840 1139.6301 1139.6299 0 0 K.TAINHVSTVAK.D**  ([Ions score 28](http://gary/mascot/cgi/peptide_view.pl?file=../data/20130722/F001266.dat&query=339&hit=1&index=gene_43|putative&px=1&section=5&ave_thresh=2&_ignoreionsscorebelow=20&report=0&_sigthreshold=0.05&_msresflags=1089&_msresflags2=2&percolate=-1&percolate_rt=0))

**184 - 194 570.8226 1139.6306 1139.6299 1 0 K.TAINHVSTVAK.D**  ([Ions score 64](http://gary/mascot/cgi/peptide_view.pl?file=../data/20130722/F001266.dat&query=340&hit=1&index=gene_43|putative&px=1&section=5&ave_thresh=2&_ignoreionsscorebelow=20&report=0&_sigthreshold=0.05&_msresflags=1089&_msresflags2=2&percolate=-1&percolate_rt=0))

**233 - 248 486.7418 1942.9382 1942.9360 1 0 R.LTDDYQPKPPFHVACR.T**  ([Ions score 23](http://gary/mascot/cgi/peptide_view.pl?file=../data/20130722/F001266.dat&query=752&hit=1&index=gene_43|putative&px=1&section=5&ave_thresh=2&_ignoreionsscorebelow=20&report=0&_sigthreshold=0.05&_msresflags=1089&_msresflags2=2&percolate=-1&percolate_rt=0))

**249 - 267 978.4836 1954.9527 1954.9524 0 0 R.TTTAPVVSSEFDFLDAGAK.R**  ([Ions score 96](http://gary/mascot/cgi/peptide_view.pl?file=../data/20130722/F001266.dat&query=760&hit=1&index=gene_43|putative&px=1&section=5&ave_thresh=2&_ignoreionsscorebelow=20&report=0&_sigthreshold=0.05&_msresflags=1089&_msresflags2=2&percolate=-1&percolate_rt=0))

**249 - 267 652.6583 1954.9530 1954.9524 0 0 R.TTTAPVVSSEFDFLDAGAK.R**  ([Ions score 55](http://gary/mascot/cgi/peptide_view.pl?file=../data/20130722/F001266.dat&query=761&hit=1&index=gene_43|putative&px=1&section=5&ave_thresh=2&_ignoreionsscorebelow=20&report=0&_sigthreshold=0.05&_msresflags=1089&_msresflags2=2&percolate=-1&percolate_rt=0))

**249 - 268 704.6894 2111.0463 2111.0535 -3 1 R.TTTAPVVSSEFDFLDAGAKR.A**  ([Ions score 58](http://gary/mascot/cgi/peptide_view.pl?file=../data/20130722/F001266.dat&query=789&hit=1&index=gene_43|putative&px=1&section=5&ave_thresh=2&_ignoreionsscorebelow=20&report=0&_sigthreshold=0.05&_msresflags=1089&_msresflags2=2&percolate=-1&percolate_rt=0))

**272 - 290 1011.4573 2020.9000 2020.9014 -1 0 R.GADGGTQVDANTSYYDFLK.Q**  ([Ions score 106](http://gary/mascot/cgi/peptide_view.pl?file=../data/20130722/F001266.dat&query=776&hit=1&index=gene_43|putative&px=1&section=5&ave_thresh=2&_ignoreionsscorebelow=20&report=0&_sigthreshold=0.05&_msresflags=1089&_msresflags2=2&percolate=-1&percolate_rt=0))

**272 - 290 674.6417 2020.9033 2020.9014 1 0 R.GADGGTQVDANTSYYDFLK.Q**  ([Ions score 63](http://gary/mascot/cgi/peptide_view.pl?file=../data/20130722/F001266.dat&query=777&hit=1&index=gene_43|putative&px=1&section=5&ave_thresh=2&_ignoreionsscorebelow=20&report=0&_sigthreshold=0.05&_msresflags=1089&_msresflags2=2&percolate=-1&percolate_rt=0))

**291 - 307 643.6579 1927.9519 1927.9541 -1 1 K.QQPAWFQDEALGPTRGK.I**  ([Ions score 44](http://gary/mascot/cgi/peptide_view.pl?file=../data/20130722/F001266.dat&query=747&hit=1&index=gene_43|putative&px=1&section=5&ave_thresh=2&_ignoreionsscorebelow=20&report=0&_sigthreshold=0.05&_msresflags=1089&_msresflags2=2&percolate=-1&percolate_rt=0))

**343 - 349 390.2190 778.4235 778.4225 1 0 K.VADYLAK.I**  ([Ions score 36](http://gary/mascot/cgi/peptide_view.pl?file=../data/20130722/F001266.dat&query=49&hit=1&index=gene_43|putative&px=1&section=5&ave_thresh=2&_ignoreionsscorebelow=20&report=0&_sigthreshold=0.05&_msresflags=1089&_msresflags2=2&percolate=-1&percolate_rt=0))

**4.**[gene_42|](http://10.139.25.109/mascot/cgi/protein_view.pl?file=../data/20100316/F010069.dat&hit=gene_42|conserved&px=1&ave_thresh=1&_sigthreshold=0.05&_server_mudpit_switch=1e-009&_ignoreionsscorebelow=20) putative portal protein|[Serratia phage Eta] **Mass:** 55097    **Score:** 3901    **Queries**

**matched:** 43(43); **emPAI:** 174.78; Sequence Coverage: **68%;** Matched peptides shown in **Bold Red**

**1** **MTTGFDTVKT AHREYTANAA KWK**K**VRDAIS GEMRKYLRNV GANEQDAAYG KQRQAEYEDG AIVYNFTKRT LSGMVGSVMR KDPEQQFPTR** MEYLNDDASG

**101** AGVGLWQHVQ DTLMEIDSVG R**GGLLVDAPN VDVATMAQQN AGQLNPIIAF YTTENIINWK** LK**RVGSVNK**V VMVVLR**ELYE YNDSPDEFTT LVGEQYRVLD**

**201 IDENGNYRQR VYKFDNSGSL QGGVLELFPK LNGVPK**GEIP FTFVGASNND DTIDDAPLLP LADLQVGHFS NSADNEEMLH TLAQAMLIIA PGTNISPQQW

**301** LELNPNGVMY GSRR**GLNVGA GGSALLLQMQ ESTALQAALT AKEQQAIQIG AQLITPTTQI TAESARLQRG ADSSVMATIA RNVSQAYEDC LKWVAQMLGL**

**401 PDSGIEFK**LN MEFFLQQMTP QEAQQWMAMV QSGYVPTEAM WEAMR**RGGWT NWTNAEMKEK IEAAPVPSIS AATPVAGDIP ASAQQPQADN QQQNDQQAQQ**

**Start - End Observed Mr(expt) Mr(calc) ppm Miss Sequence**

**1 - 9 500.2442 998.4739 998.4743 0 0 -.MTTGFDTVK.T**  ([Ions score 40](http://gary/mascot/cgi/peptide_view.pl?file=../data/20130722/F001267.dat&query=182&hit=1&index=gene_42|conserved&px=1&section=5&ave_thresh=3&_ignoreionsscorebelow=20&report=0&_sigthreshold=0.05&_msresflags=1089&_msresflags2=2&percolate=-1&percolate_rt=0))

**1 - 9 508.2433 1014.4720 1014.4692 3 0 -.MTTGFDTVK.T**  Oxidation (M) ([Ions score 45](http://gary/mascot/cgi/peptide_view.pl?file=../data/20130722/F001267.dat&query=196&hit=1&index=gene_42|conserved&px=1&section=5&ave_thresh=3&_ignoreionsscorebelow=20&report=0&_sigthreshold=0.05&_msresflags=1089&_msresflags2=2&percolate=-1&percolate_rt=0))

**2 - 9 434.7242 867.4339 867.4338 0 0 M.TTGFDTVK.T**  ([Ions score 28](http://gary/mascot/cgi/peptide_view.pl?file=../data/20130722/F001267.dat&query=91&hit=1&index=gene_42|conserved&px=1&section=5&ave_thresh=3&_ignoreionsscorebelow=20&report=0&_sigthreshold=0.05&_msresflags=1089&_msresflags2=2&percolate=-1&percolate_rt=0))

**10 - 21 444.8934 1331.6583 1331.6582 0 1 K.TAHREYTANAAK.W**  ([Ions score 41](http://gary/mascot/cgi/peptide_view.pl?file=../data/20130722/F001267.dat&query=522&hit=1&index=gene_42|conserved&px=1&section=5&ave_thresh=3&_ignoreionsscorebelow=20&report=0&_sigthreshold=0.05&_msresflags=1089&_msresflags2=2&percolate=-1&percolate_rt=0))

**10 - 21 666.8371 1331.6596 1331.6582 1 1 K.TAHREYTANAAK.W**  ([Ions score 53](http://gary/mascot/cgi/peptide_view.pl?file=../data/20130722/F001267.dat&query=523&hit=1&index=gene_42|conserved&px=1&section=5&ave_thresh=3&_ignoreionsscorebelow=20&report=0&_sigthreshold=0.05&_msresflags=1089&_msresflags2=2&percolate=-1&percolate_rt=0))

**10 - 23 412.4647 1645.8298 1645.8324 -2 2 K.TAHREYTANAAKWK.K**  ([Ions score 24](http://gary/mascot/cgi/peptide_view.pl?file=../data/20130722/F001267.dat&query=660&hit=1&index=gene_42|conserved&px=1&section=5&ave_thresh=3&_ignoreionsscorebelow=20&report=0&_sigthreshold=0.05&_msresflags=1089&_msresflags2=2&percolate=-1&percolate_rt=0))

**14 - 23 591.3012 1180.5877 1180.5876 0 1 R.EYTANAAKWK.K**  ([Ions score 38](http://gary/mascot/cgi/peptide_view.pl?file=../data/20130722/F001267.dat&query=363&hit=1&index=gene_42|conserved&px=1&section=5&ave_thresh=3&_ignoreionsscorebelow=20&report=0&_sigthreshold=0.05&_msresflags=1089&_msresflags2=2&percolate=-1&percolate_rt=0))

**25 - 34 378.5292 1132.5658 1132.5659 0 1 K.VRDAISGEMR.K**  ([Ions score 23](http://gary/mascot/cgi/peptide_view.pl?file=../data/20130722/F001267.dat&query=310&hit=1&index=gene_42|conserved&px=1&section=5&ave_thresh=3&_ignoreionsscorebelow=20&report=0&_sigthreshold=0.05&_msresflags=1089&_msresflags2=2&percolate=-1&percolate_rt=0))

**25 - 34 567.2913 1132.5680 1132.5659 2 1 K.VRDAISGEMR.K**  ([Ions score 57](http://gary/mascot/cgi/peptide_view.pl?file=../data/20130722/F001267.dat&query=311&hit=1&index=gene_42|conserved&px=1&section=5&ave_thresh=3&_ignoreionsscorebelow=20&report=0&_sigthreshold=0.05&_msresflags=1089&_msresflags2=2&percolate=-1&percolate_rt=0))

**25 - 34 575.2869 1148.5592 1148.5608 -1 1 K.VRDAISGEMR.K**  Oxidation (M) ([Ions score 61](http://gary/mascot/cgi/peptide_view.pl?file=../data/20130722/F001267.dat&query=328&hit=1&index=gene_42|conserved&px=1&section=5&ave_thresh=3&_ignoreionsscorebelow=20&report=0&_sigthreshold=0.05&_msresflags=1089&_msresflags2=2&percolate=-1&percolate_rt=0))

**25 - 34 383.8608 1148.5605 1148.5608 0 1 K.VRDAISGEMR.K**  Oxidation (M) ([Ions score 28](http://gary/mascot/cgi/peptide_view.pl?file=../data/20130722/F001267.dat&query=329&hit=1&index=gene_42|conserved&px=1&section=5&ave_thresh=3&_ignoreionsscorebelow=20&report=0&_sigthreshold=0.05&_msresflags=1089&_msresflags2=2&percolate=-1&percolate_rt=0))

**25 - 35 421.2280 1260.6623 1260.6608 1 2 K.VRDAISGEMRK.Y**  ([Ions score 34](http://gary/mascot/cgi/peptide_view.pl?file=../data/20130722/F001267.dat&query=445&hit=1&index=gene_42|conserved&px=1&section=5&ave_thresh=3&_ignoreionsscorebelow=20&report=0&_sigthreshold=0.05&_msresflags=1089&_msresflags2=2&percolate=-1&percolate_rt=0))

**25 - 35 639.3331 1276.6517 1276.6557 -3 2 K.VRDAISGEMRK.Y**  Oxidation (M) ([Ions score 35](http://gary/mascot/cgi/peptide_view.pl?file=../data/20130722/F001267.dat&query=461&hit=1&index=gene_42|conserved&px=1&section=5&ave_thresh=3&_ignoreionsscorebelow=20&report=0&_sigthreshold=0.05&_msresflags=1089&_msresflags2=2&percolate=-1&percolate_rt=0))

**25 - 35 426.5591 1276.6554 1276.6557 0 2 K.VRDAISGEMRK.Y**  Oxidation (M) ([Ions score 41](http://gary/mascot/cgi/peptide_view.pl?file=../data/20130722/F001267.dat&query=462&hit=1&index=gene_42|conserved&px=1&section=5&ave_thresh=3&_ignoreionsscorebelow=20&report=0&_sigthreshold=0.05&_msresflags=1089&_msresflags2=2&percolate=-1&percolate_rt=0))

**27 - 34 439.7057 877.3968 877.3963 1 0 R.DAISGEMR.K**  ([Ions score 38](http://gary/mascot/cgi/peptide_view.pl?file=../data/20130722/F001267.dat&query=97&hit=1&index=gene_42|conserved&px=1&section=5&ave_thresh=3&_ignoreionsscorebelow=20&report=0&_sigthreshold=0.05&_msresflags=1089&_msresflags2=2&percolate=-1&percolate_rt=0))

**27 - 35 336.1712 1005.4917 1005.4913 0 1 R.DAISGEMRK.Y**  ([Ions score 37](http://gary/mascot/cgi/peptide_view.pl?file=../data/20130722/F001267.dat&query=190&hit=1&index=gene_42|conserved&px=1&section=5&ave_thresh=3&_ignoreionsscorebelow=20&report=0&_sigthreshold=0.05&_msresflags=1089&_msresflags2=2&percolate=-1&percolate_rt=0))

**27 - 35 503.7534 1005.4923 1005.4913 1 1 R.DAISGEMRK.Y**  ([Ions score 39](http://gary/mascot/cgi/peptide_view.pl?file=../data/20130722/F001267.dat&query=191&hit=1&index=gene_42|conserved&px=1&section=5&ave_thresh=3&_ignoreionsscorebelow=20&report=0&_sigthreshold=0.05&_msresflags=1089&_msresflags2=2&percolate=-1&percolate_rt=0))

**27 - 35 511.7502 1021.4859 1021.4862 0 1 R.DAISGEMRK.Y**  Oxidation (M) ([Ions score 49](http://gary/mascot/cgi/peptide_view.pl?file=../data/20130722/F001267.dat&query=201&hit=1&index=gene_42|conserved&px=1&section=5&ave_thresh=3&_ignoreionsscorebelow=20&report=0&_sigthreshold=0.05&_msresflags=1089&_msresflags2=2&percolate=-1&percolate_rt=0))

**27 - 35 341.5026 1021.4860 1021.4862 0 1 R.DAISGEMRK.Y**  Oxidation (M) ([Ions score 22](http://gary/mascot/cgi/peptide_view.pl?file=../data/20130722/F001267.dat&query=202&hit=1&index=gene_42|conserved&px=1&section=5&ave_thresh=3&_ignoreionsscorebelow=20&report=0&_sigthreshold=0.05&_msresflags=1089&_msresflags2=2&percolate=-1&percolate_rt=0))

**36 - 51 884.9329 1767.8513 1767.8540 -2 1 K.YLRNVGANEQDAAYGK.Q**  ([Ions score 91](http://gary/mascot/cgi/peptide_view.pl?file=../data/20130722/F001267.dat&query=697&hit=1&index=gene_42|conserved&px=1&section=5&ave_thresh=3&_ignoreionsscorebelow=20&report=0&_sigthreshold=0.05&_msresflags=1089&_msresflags2=2&percolate=-1&percolate_rt=0))

**36 - 51 590.2931 1767.8574 1767.8540 2 1 K.YLRNVGANEQDAAYGK.Q**  ([Ions score 63](http://gary/mascot/cgi/peptide_view.pl?file=../data/20130722/F001267.dat&query=698&hit=1&index=gene_42|conserved&px=1&section=5&ave_thresh=3&_ignoreionsscorebelow=20&report=0&_sigthreshold=0.05&_msresflags=1089&_msresflags2=2&percolate=-1&percolate_rt=0))

**39 - 51 668.8098 1335.6051 1335.6055 0 0 R.NVGANEQDAAYGK.Q**  ([Ions score 85](http://gary/mascot/cgi/peptide_view.pl?file=../data/20130722/F001267.dat&query=525&hit=1&index=gene_42|conserved&px=1&section=5&ave_thresh=3&_ignoreionsscorebelow=20&report=0&_sigthreshold=0.05&_msresflags=1089&_msresflags2=2&percolate=-1&percolate_rt=0))

**39 - 51 669.3060 1336.5975 1336.5895 6 0 R.NVGANEQDAAYGK.Q**  Deamidated (NQ) ([Ions score 82](http://gary/mascot/cgi/peptide_view.pl?file=../data/20130722/F001267.dat&query=527&hit=1&index=gene_42|conserved&px=1&section=5&ave_thresh=3&_ignoreionsscorebelow=20&report=0&_sigthreshold=0.05&_msresflags=1089&_msresflags2=2&percolate=-1&percolate_rt=0))

**39 - 53 810.8888 1619.7630 1619.7652 -1 1 R.NVGANEQDAAYGKQR.Q**  ([Ions score 95](http://gary/mascot/cgi/peptide_view.pl?file=../data/20130722/F001267.dat&query=651&hit=1&index=gene_42|conserved&px=1&section=5&ave_thresh=3&_ignoreionsscorebelow=20&report=0&_sigthreshold=0.05&_msresflags=1089&_msresflags2=2&percolate=-1&percolate_rt=0))

**39 - 53 540.9293 1619.7660 1619.7652 0 1 R.NVGANEQDAAYGKQR.Q**  ([Ions score 20](http://gary/mascot/cgi/peptide_view.pl?file=../data/20130722/F001267.dat&query=652&hit=1&index=gene_42|conserved&px=1&section=5&ave_thresh=3&_ignoreionsscorebelow=20&report=0&_sigthreshold=0.05&_msresflags=1089&_msresflags2=2&percolate=-1&percolate_rt=0))

**52 - 68 1007.9778 2013.9411 2013.9432 -1 1 K.QRQAEYEDGAIVYNFTK.R** Gln->pyro-Glu(N-term Q)([Ions score 88](http://gary/mascot/cgi/peptide_view.pl?file=../data/20130722/F001267.dat&query=770&hit=1&index=gene_42|conserved&px=1&section=5&ave_thresh=3&_ignoreionsscorebelow=20&report=0&_sigthreshold=0.05&_msresflags=1089&_msresflags2=2&percolate=-1&percolate_rt=0))

**52 - 68 672.3246 2013.9519 2013.9432 4 1 K.QRQAEYEDGAIVYNFTK.R** Gln->pyro-Glu(N-term Q)([Ions score 55](http://gary/mascot/cgi/peptide_view.pl?file=../data/20130722/F001267.dat&query=771&hit=1&index=gene_42|conserved&px=1&section=5&ave_thresh=3&_ignoreionsscorebelow=20&report=0&_sigthreshold=0.05&_msresflags=1089&_msresflags2=2&percolate=-1&percolate_rt=0))

**52 - 68 1016.4901 2030.9655 2030.9697 -2 1 K.QRQAEYEDGAIVYNFTK.R**  ([Ions score 59](http://gary/mascot/cgi/peptide_view.pl?file=../data/20130722/F001267.dat&query=773&hit=1&index=gene_42|conserved&px=1&section=5&ave_thresh=3&_ignoreionsscorebelow=20&report=0&_sigthreshold=0.05&_msresflags=1089&_msresflags2=2&percolate=-1&percolate_rt=0))

**52 - 68 677.9972 2030.9697 2030.9697 0 1 K.QRQAEYEDGAIVYNFTK.R**  ([Ions score 61](http://gary/mascot/cgi/peptide_view.pl?file=../data/20130722/F001267.dat&query=774&hit=1&index=gene_42|conserved&px=1&section=5&ave_thresh=3&_ignoreionsscorebelow=20&report=0&_sigthreshold=0.05&_msresflags=1089&_msresflags2=2&percolate=-1&percolate_rt=0))

**54 - 68 865.9013 1729.7881 1729.7835 3 0 R.QAEYEDGAIVYNFTK.R**  Gln->pyro-Glu (N-term Q)([Ions score 46](http://gary/mascot/cgi/peptide_view.pl?file=../data/20130722/F001267.dat&query=682&hit=1&index=gene_42|conserved&px=1&section=5&ave_thresh=3&_ignoreionsscorebelow=20&report=0&_sigthreshold=0.05&_msresflags=1089&_msresflags2=2&percolate=-1&percolate_rt=0))

**54 - 68 583.2767 1746.8084 1746.8101 -1 0 R.QAEYEDGAIVYNFTK.R**  ([Ions score 44](http://gary/mascot/cgi/peptide_view.pl?file=../data/20130722/F001267.dat&query=686&hit=1&index=gene_42|conserved&px=1&section=5&ave_thresh=3&_ignoreionsscorebelow=20&report=0&_sigthreshold=0.05&_msresflags=1089&_msresflags2=2&percolate=-1&percolate_rt=0))

**54 - 68 874.4131 1746.8116 1746.8101 1 0 R.QAEYEDGAIVYNFTK.R**  ([Ions score 94](http://gary/mascot/cgi/peptide_view.pl?file=../data/20130722/F001267.dat&query=687&hit=1&index=gene_42|conserved&px=1&section=5&ave_thresh=3&_ignoreionsscorebelow=20&report=0&_sigthreshold=0.05&_msresflags=1089&_msresflags2=2&percolate=-1&percolate_rt=0))

**54 - 69 943.9514 1885.8881 1885.8846 2 1 R.QAEYEDGAIVYNFTKR.T**  Gln->pyro-Glu(N-term Q)([Ions score 99](http://gary/mascot/cgi/peptide_view.pl?file=../data/20130722/F001267.dat&query=739&hit=1&index=gene_42|conserved&px=1&section=5&ave_thresh=3&_ignoreionsscorebelow=20&report=0&_sigthreshold=0.05&_msresflags=1089&_msresflags2=2&percolate=-1&percolate_rt=0))

**54 - 69 952.4622 1902.9098 1902.9112 -1 1 R.QAEYEDGAIVYNFTKR.T**  ([Ions score 103](http://gary/mascot/cgi/peptide_view.pl?file=../data/20130722/F001267.dat&query=743&hit=1&index=gene_42|conserved&px=1&section=5&ave_thresh=3&_ignoreionsscorebelow=20&report=0&_sigthreshold=0.05&_msresflags=1089&_msresflags2=2&percolate=-1&percolate_rt=0))

**54 - 69 635.3126 1902.9159 1902.9112 2 1 R.QAEYEDGAIVYNFTKR.T**  ([Ions score 43](http://gary/mascot/cgi/peptide_view.pl?file=../data/20130722/F001267.dat&query=744&hit=1&index=gene_42|conserved&px=1&section=5&ave_thresh=3&_ignoreionsscorebelow=20&report=0&_sigthreshold=0.05&_msresflags=1089&_msresflags2=2&percolate=-1&percolate_rt=0))

**69 - 80 647.3436 1292.6727 1292.6693 3 1 K.RTLSGMVGSVMR.K**  ([Ions score 70](http://gary/mascot/cgi/peptide_view.pl?file=../data/20130722/F001267.dat&query=483&hit=1&index=gene_42|conserved&px=1&section=5&ave_thresh=3&_ignoreionsscorebelow=20&report=0&_sigthreshold=0.05&_msresflags=1089&_msresflags2=2&percolate=-1&percolate_rt=0))

**69 - 80 431.8988 1292.6745 1292.6693 4 1 K.RTLSGMVGSVMR.K**  ([Ions score 26](http://gary/mascot/cgi/peptide_view.pl?file=../data/20130722/F001267.dat&query=484&hit=1&index=gene_42|conserved&px=1&section=5&ave_thresh=3&_ignoreionsscorebelow=20&report=0&_sigthreshold=0.05&_msresflags=1089&_msresflags2=2&percolate=-1&percolate_rt=0))

**69 - 80 655.3409 1308.6672 1308.6642 2 1 K.RTLSGMVGSVMR.K**  Oxidation (M) ([Ions score 39](http://gary/mascot/cgi/peptide_view.pl?file=../data/20130722/F001267.dat&query=504&hit=1&index=gene_42|conserved&px=1&section=5&ave_thresh=3&_ignoreionsscorebelow=20&report=0&_sigthreshold=0.05&_msresflags=1089&_msresflags2=2&percolate=-1&percolate_rt=0))

**69 - 81 474.5947 1420.7623 1420.7643 -1 2 K.RTLSGMVGSVMRK.D**  ([Ions score 50](http://gary/mascot/cgi/peptide_view.pl?file=../data/20130722/F001267.dat&query=568&hit=1&index=gene_42|conserved&px=1&section=5&ave_thresh=3&_ignoreionsscorebelow=20&report=0&_sigthreshold=0.05&_msresflags=1089&_msresflags2=2&percolate=-1&percolate_rt=0))

**69 - 81 479.9269 1436.7588 1436.7592 0 2 K.RTLSGMVGSVMRK.D**  Oxidation (M) ([Ions score 56](http://gary/mascot/cgi/peptide_view.pl?file=../data/20130722/F001267.dat&query=574&hit=1&index=gene_42|conserved&px=1&section=5&ave_thresh=3&_ignoreionsscorebelow=20&report=0&_sigthreshold=0.05&_msresflags=1089&_msresflags2=2&percolate=-1&percolate_rt=0))

**69 - 81 485.2584 1452.7535 1452.7541 0 2 K.RTLSGMVGSVMRK.D**  2 Oxidation (M) ([Ions score 32](http://gary/mascot/cgi/peptide_view.pl?file=../data/20130722/F001267.dat&query=581&hit=1&index=gene_42|conserved&px=1&section=5&ave_thresh=3&_ignoreionsscorebelow=20&report=0&_sigthreshold=0.05&_msresflags=1089&_msresflags2=2&percolate=-1&percolate_rt=0))

**70 - 80 569.2918 1136.5691 1136.5682 1 0 R.TLSGMVGSVMR.K**  ([Ions score 95](http://gary/mascot/cgi/peptide_view.pl?file=../data/20130722/F001267.dat&query=315&hit=1&index=gene_42|conserved&px=1&section=5&ave_thresh=3&_ignoreionsscorebelow=20&report=0&_sigthreshold=0.05&_msresflags=1089&_msresflags2=2&percolate=-1&percolate_rt=0))

**70 - 80 585.2866 1168.5586 1168.5580 0 0 R.TLSGMVGSVMR.K**  2 Oxidation (M) ([Ions score 76](http://gary/mascot/cgi/peptide_view.pl?file=../data/20130722/F001267.dat&query=354&hit=1&index=gene_42|conserved&px=1&section=5&ave_thresh=3&_ignoreionsscorebelow=20&report=0&_sigthreshold=0.05&_msresflags=1089&_msresflags2=2&percolate=-1&percolate_rt=0))

**70 - 81 633.3400 1264.6655 1264.6632 2 1 R.TLSGMVGSVMRK.D**  ([Ions score 58](http://gary/mascot/cgi/peptide_view.pl?file=../data/20130722/F001267.dat&query=449&hit=1&index=gene_42|conserved&px=1&section=5&ave_thresh=3&_ignoreionsscorebelow=20&report=0&_sigthreshold=0.05&_msresflags=1089&_msresflags2=2&percolate=-1&percolate_rt=0))

**70 - 81 641.3345 1280.6544 1280.6581 -3 1 R.TLSGMVGSVMRK.D**  Oxidation (M) ([Ions score 42](http://gary/mascot/cgi/peptide_view.pl?file=../data/20130722/F001267.dat&query=468&hit=1&index=gene_42|conserved&px=1&section=5&ave_thresh=3&_ignoreionsscorebelow=20&report=0&_sigthreshold=0.05&_msresflags=1089&_msresflags2=2&percolate=-1&percolate_rt=0))

**81 - 90 415.8786 1244.6141 1244.6149 -1 1 R.KDPEQQFPTR.M**  ([Ions score 28](http://gary/mascot/cgi/peptide_view.pl?file=../data/20130722/F001267.dat&query=432&hit=1&index=gene_42|conserved&px=1&section=5&ave_thresh=3&_ignoreionsscorebelow=20&report=0&_sigthreshold=0.05&_msresflags=1089&_msresflags2=2&percolate=-1&percolate_rt=0))

**81 - 90 623.3156 1244.6165 1244.6149 1 1 R.KDPEQQFPTR.M**  ([Ions score 50](http://gary/mascot/cgi/peptide_view.pl?file=../data/20130722/F001267.dat&query=433&hit=1&index=gene_42|conserved&px=1&section=5&ave_thresh=3&_ignoreionsscorebelow=20&report=0&_sigthreshold=0.05&_msresflags=1089&_msresflags2=2&percolate=-1&percolate_rt=0))

**82 - 90 559.2672 1116.5199 1116.5200 0 0 K.DPEQQFPTR.M**  ([Ions score 52](http://gary/mascot/cgi/peptide_view.pl?file=../data/20130722/F001267.dat&query=287&hit=1&index=gene_42|conserved&px=1&section=5&ave_thresh=3&_ignoreionsscorebelow=20&report=0&_sigthreshold=0.05&_msresflags=1089&_msresflags2=2&percolate=-1&percolate_rt=0))

**122 - 160 1054.7966 4215.1574 4215.1310 6 0 R.GGLLVDAPNVDVATMAQQNAGQLNPIIAFYTTENIINWK.L**

Oxidation (M) ([Ions score 104](http://gary/mascot/cgi/peptide_view.pl?file=../data/20130722/F001267.dat&query=970&hit=1&index=gene_42|conserved&px=1&section=5&ave_thresh=3&_ignoreionsscorebelow=20&report=0&_sigthreshold=0.05&_msresflags=1089&_msresflags2=2&percolate=-1&percolate_rt=0))

**163 - 169 380.2273 758.4400 758.4399 0 1 K.RVGSVNK.V**  ([Ions score 24](http://gary/mascot/cgi/peptide_view.pl?file=../data/20130722/F001267.dat&query=38&hit=1&index=gene_42|conserved&px=1&section=5&ave_thresh=3&_ignoreionsscorebelow=20&report=0&_sigthreshold=0.05&_msresflags=1089&_msresflags2=2&percolate=-1&percolate_rt=0))

**177 - 197 856.7175 2567.1308 2567.1340 -1 0 R.ELYEYNDSPDEFTTLVGEQYR.V**  ([Ions score 46](http://gary/mascot/cgi/peptide_view.pl?file=../data/20130722/F001267.dat&query=893&hit=1&index=gene_42|conserved&px=1&section=5&ave_thresh=3&_ignoreionsscorebelow=20&report=0&_sigthreshold=0.05&_msresflags=1089&_msresflags2=2&percolate=-1&percolate_rt=0))

**177 - 197 1284.5762 2567.1378 2567.1340 1 0 R.ELYEYNDSPDEFTTLVGEQYR.V**  ([Ions score 99](http://gary/mascot/cgi/peptide_view.pl?file=../data/20130722/F001267.dat&query=894&hit=1&index=gene_42|conserved&px=1&section=5&ave_thresh=3&_ignoreionsscorebelow=20&report=0&_sigthreshold=0.05&_msresflags=1089&_msresflags2=2&percolate=-1&percolate_rt=0))

**198 - 208 654.3152 1306.6158 1306.6153 0 0 R.VLDIDENGNYR.Q**  ([Ions score 72](http://gary/mascot/cgi/peptide_view.pl?file=../data/20130722/F001267.dat&query=498&hit=1&index=gene_42|conserved&px=1&section=5&ave_thresh=3&_ignoreionsscorebelow=20&report=0&_sigthreshold=0.05&_msresflags=1089&_msresflags2=2&percolate=-1&percolate_rt=0))

**198 - 208 654.8070 1307.5995 1307.5993 0 0 R.VLDIDENGNYR.Q**  Deamidated (NQ) ([Ions score 81](http://gary/mascot/cgi/peptide_view.pl?file=../data/20130722/F001267.dat&query=500&hit=1&index=gene_42|conserved&px=1&section=5&ave_thresh=3&_ignoreionsscorebelow=20&report=0&_sigthreshold=0.05&_msresflags=1089&_msresflags2=2&percolate=-1&percolate_rt=0))

**198 - 210 531.2656 1590.7749 1590.7750 0 1 R.VLDIDENGNYRQR.V**  ([Ions score 34](http://gary/mascot/cgi/peptide_view.pl?file=../data/20130722/F001267.dat&query=634&hit=1&index=gene_42|conserved&px=1&section=5&ave_thresh=3&_ignoreionsscorebelow=20&report=0&_sigthreshold=0.05&_msresflags=1089&_msresflags2=2&percolate=-1&percolate_rt=0))

**198 - 210 796.3963 1590.7780 1590.7750 2 1 R.VLDIDENGNYRQR.V**  ([Ions score 37](http://gary/mascot/cgi/peptide_view.pl?file=../data/20130722/F001267.dat&query=635&hit=1&index=gene_42|conserved&px=1&section=5&ave_thresh=3&_ignoreionsscorebelow=20&report=0&_sigthreshold=0.05&_msresflags=1089&_msresflags2=2&percolate=-1&percolate_rt=0))

**198 - 210 531.5964 1591.7673 1591.7590 5 1 R.VLDIDENGNYRQR.V**  Deamidated (NQ) ([Ions score 33](http://gary/mascot/cgi/peptide_view.pl?file=../data/20130722/F001267.dat&query=636&hit=1&index=gene_42|conserved&px=1&section=5&ave_thresh=3&_ignoreionsscorebelow=20&report=0&_sigthreshold=0.05&_msresflags=1089&_msresflags2=2&percolate=-1&percolate_rt=0))

**198 - 210 796.8911 1591.7677 1591.7590 5 1 R.VLDIDENGNYRQR.V**  Deamidated (NQ) ([Ions score 44](http://gary/mascot/cgi/peptide_view.pl?file=../data/20130722/F001267.dat&query=637&hit=1&index=gene_42|conserved&px=1&section=5&ave_thresh=3&_ignoreionsscorebelow=20&report=0&_sigthreshold=0.05&_msresflags=1089&_msresflags2=2&percolate=-1&percolate_rt=0))

**211 - 230 733.3885 2197.1436 2197.1419 1 1 R.VYKFDNSGSLQGGVLELFPK.L**  ([Ions score 48](http://gary/mascot/cgi/peptide_view.pl?file=../data/20130722/F001267.dat&query=801&hit=1&index=gene_42|conserved&px=1&section=5&ave_thresh=3&_ignoreionsscorebelow=20&report=0&_sigthreshold=0.05&_msresflags=1089&_msresflags2=2&percolate=-1&percolate_rt=0))

**211 - 236 702.6331 2806.5031 2806.4905 4 2 R.VYKFDNSGSLQGGVLELFPKLNGVPK.G** Deamidated(NQ)([Ions score 67](http://gary/mascot/cgi/peptide_view.pl?file=../data/20130722/F001267.dat&query=917&hit=1&index=gene_42|conserved&px=1&section=5&ave_thresh=3&_ignoreionsscorebelow=20&report=0&_sigthreshold=0.05&_msresflags=1089&_msresflags2=2&percolate=-1&percolate_rt=0))

**214 - 230 603.3107 1806.9104 1806.9152 -3 0 K.FDNSGSLQGGVLELFPK.L**  ([Ions score 72](http://gary/mascot/cgi/peptide_view.pl?file=../data/20130722/F001267.dat&query=709&hit=1&index=gene_42|conserved&px=1&section=5&ave_thresh=3&_ignoreionsscorebelow=20&report=0&_sigthreshold=0.05&_msresflags=1089&_msresflags2=2&percolate=-1&percolate_rt=0))

**214 - 230 904.4639 1806.9133 1806.9152 -1 0 K.FDNSGSLQGGVLELFPK.L**  ([Ions score 93](http://gary/mascot/cgi/peptide_view.pl?file=../data/20130722/F001267.dat&query=710&hit=1&index=gene_42|conserved&px=1&section=5&ave_thresh=3&_ignoreionsscorebelow=20&report=0&_sigthreshold=0.05&_msresflags=1089&_msresflags2=2&percolate=-1&percolate_rt=0))

**214 - 230 904.9599 1807.9052 1807.8992 3 0 K.FDNSGSLQGGVLELFPK.L**  Deamidated (NQ) ([Ions score 87](http://gary/mascot/cgi/peptide_view.pl?file=../data/20130722/F001267.dat&query=711&hit=1&index=gene_42|conserved&px=1&section=5&ave_thresh=3&_ignoreionsscorebelow=20&report=0&_sigthreshold=0.05&_msresflags=1089&_msresflags2=2&percolate=-1&percolate_rt=0))

**214 - 236 1209.1403 2416.2660 2416.2638 1 1 K.FDNSGSLQGGVLELFPKLNGVPK.G**  Deamidated (NQ) ([Ions score 63](http://gary/mascot/cgi/peptide_view.pl?file=../data/20130722/F001267.dat&query=846&hit=1&index=gene_42|conserved&px=1&section=5&ave_thresh=3&_ignoreionsscorebelow=20&report=0&_sigthreshold=0.05&_msresflags=1089&_msresflags2=2&percolate=-1&percolate_rt=0))

**214 - 236 605.0739 2416.2665 2416.2638 1 1 K.FDNSGSLQGGVLELFPKLNGVPK.G**  Deamidated (NQ) ([Ions score 26](http://gary/mascot/cgi/peptide_view.pl?file=../data/20130722/F001267.dat&query=847&hit=1&index=gene_42|conserved&px=1&section=5&ave_thresh=3&_ignoreionsscorebelow=20&report=0&_sigthreshold=0.05&_msresflags=1089&_msresflags2=2&percolate=-1&percolate_rt=0))

**214 - 236 806.4315 2416.2726 2416.2638 4 1 K.FDNSGSLQGGVLELFPKLNGVPK.G**  Deamidated (NQ) ([Ions score 51](http://gary/mascot/cgi/peptide_view.pl?file=../data/20130722/F001267.dat&query=848&hit=1&index=gene_42|conserved&px=1&section=5&ave_thresh=3&_ignoreionsscorebelow=20&report=0&_sigthreshold=0.05&_msresflags=1089&_msresflags2=2&percolate=-1&percolate_rt=0))

**231 - 236 314.1948 626.3750 626.3752 0 0 K.LNGVPK.G**  ([Ions score 50](http://gary/mascot/cgi/peptide_view.pl?file=../data/20130722/F001267.dat&query=5&hit=1&index=gene_42|conserved&px=1&section=5&ave_thresh=3&_ignoreionsscorebelow=20&report=0&_sigthreshold=0.05&_msresflags=1089&_msresflags2=2&percolate=-1&percolate_rt=0))

**231 - 236 314.6869 627.3592 627.3592 0 0 K.LNGVPK.G**  Deamidated (NQ) ([Ions score 37](http://gary/mascot/cgi/peptide_view.pl?file=../data/20130722/F001267.dat&query=6&hit=1&index=gene_42|conserved&px=1&section=5&ave_thresh=3&_ignoreionsscorebelow=20&report=0&_sigthreshold=0.05&_msresflags=1089&_msresflags2=2&percolate=-1&percolate_rt=0))

**315 - 342 910.4873 2728.4401 2728.4429 -1 0 R.GLNVGAGGSALLLQMQESTALQAALTAK.E**Oxidation(M)([Ions score 112](http://gary/mascot/cgi/peptide_view.pl?file=../data/20130722/F001267.dat&query=913&hit=1&index=gene_42|conserved&px=1&section=5&ave_thresh=3&_ignoreionsscorebelow=20&report=0&_sigthreshold=0.05&_msresflags=1089&_msresflags2=2&percolate=-1&percolate_rt=0))

**343 - 366 856.7929 2567.3569 2567.3555 1 0 K.EQQAIQIGAQLITPTTQITAESAR.L**  ([Ions score 74](http://gary/mascot/cgi/peptide_view.pl?file=../data/20130722/F001267.dat&query=895&hit=1&index=gene_42|conserved&px=1&section=5&ave_thresh=3&_ignoreionsscorebelow=20&report=0&_sigthreshold=0.05&_msresflags=1089&_msresflags2=2&percolate=-1&percolate_rt=0))

**343 - 366 856.7947 2567.3624 2567.3555 3 0 K.EQQAIQIGAQLITPTTQITAESAR.L**  ([Ions score 39](http://gary/mascot/cgi/peptide_view.pl?file=../data/20130722/F001267.dat&query=896&hit=1&index=gene_42|conserved&px=1&section=5&ave_thresh=3&_ignoreionsscorebelow=20&report=0&_sigthreshold=0.05&_msresflags=1089&_msresflags2=2&percolate=-1&percolate_rt=0))

**367 - 381 525.9468 1574.8187 1574.8199 -1 1 R.LQRGADSSVMATIAR.N**  ([Ions score 36](http://gary/mascot/cgi/peptide_view.pl?file=../data/20130722/F001267.dat&query=630&hit=1&index=gene_42|conserved&px=1&section=5&ave_thresh=3&_ignoreionsscorebelow=20&report=0&_sigthreshold=0.05&_msresflags=1089&_msresflags2=2&percolate=-1&percolate_rt=0))

**370 - 381 589.7989 1177.5832 1177.5761 6 0 R.GADSSVMATIAR.N**  ([Ions score 64](http://gary/mascot/cgi/peptide_view.pl?file=../data/20130722/F001267.dat&query=358&hit=1&index=gene_42|conserved&px=1&section=5&ave_thresh=3&_ignoreionsscorebelow=20&report=0&_sigthreshold=0.05&_msresflags=1089&_msresflags2=2&percolate=-1&percolate_rt=0))

**370 - 381 597.7916 1193.5686 1193.5710 -2 0 R.GADSSVMATIAR.N**  Oxidation (M) ([Ions score 21](http://gary/mascot/cgi/peptide_view.pl?file=../data/20130722/F001267.dat&query=378&hit=1&index=gene_42|conserved&px=1&section=5&ave_thresh=3&_ignoreionsscorebelow=20&report=0&_sigthreshold=0.05&_msresflags=1089&_msresflags2=2&percolate=-1&percolate_rt=0))

**370 - 392 829.3922 2485.1546 2485.1577 -1 1 R.GADSSVMATIARNVSQAYEDCLK.W**  ([Ions score 77](http://gary/mascot/cgi/peptide_view.pl?file=../data/20130722/F001267.dat&query=857&hit=1&index=gene_42|conserved&px=1&section=5&ave_thresh=3&_ignoreionsscorebelow=20&report=0&_sigthreshold=0.05&_msresflags=1089&_msresflags2=2&percolate=-1&percolate_rt=0))

**370 - 392 834.7239 2501.1498 2501.1526 -1 1 R.GADSSVMATIARNVSQAYEDCLK.W**  Oxidation (M) ([Ions score 81](http://gary/mascot/cgi/peptide_view.pl?file=../data/20130722/F001267.dat&query=860&hit=1&index=gene_42|conserved&px=1&section=5&ave_thresh=3&_ignoreionsscorebelow=20&report=0&_sigthreshold=0.05&_msresflags=1089&_msresflags2=2&percolate=-1&percolate_rt=0))

**370 - 392 626.2950 2501.1508 2501.1526 -1 1 R.GADSSVMATIARNVSQAYEDCLK.W**  Oxidation (M) ([Ions score 34](http://gary/mascot/cgi/peptide_view.pl?file=../data/20130722/F001267.dat&query=861&hit=1&index=gene_42|conserved&px=1&section=5&ave_thresh=3&_ignoreionsscorebelow=20&report=0&_sigthreshold=0.05&_msresflags=1089&_msresflags2=2&percolate=-1&percolate_rt=0))

**382 - 392 663.8027 1325.5909 1325.5921 -1 0 R.NVSQAYEDCLK.W**  ([Ions score 55](http://gary/mascot/cgi/peptide_view.pl?file=../data/20130722/F001267.dat&query=518&hit=1&index=gene_42|conserved&px=1&section=5&ave_thresh=3&_ignoreionsscorebelow=20&report=0&_sigthreshold=0.05&_msresflags=1089&_msresflags2=2&percolate=-1&percolate_rt=0))

**393 - 408 895.9606 1789.9066 1789.9073 0 0 K.WVAQMLGLPDSGIEFK.L**  ([Ions score 82](http://gary/mascot/cgi/peptide_view.pl?file=../data/20130722/F001267.dat&query=702&hit=1&index=gene_42|conserved&px=1&section=5&ave_thresh=3&_ignoreionsscorebelow=20&report=0&_sigthreshold=0.05&_msresflags=1089&_msresflags2=2&percolate=-1&percolate_rt=0))

**393 - 408 597.6439 1789.9099 1789.9073 1 0 K.WVAQMLGLPDSGIEFK.L**  ([Ions score 41](http://gary/mascot/cgi/peptide_view.pl?file=../data/20130722/F001267.dat&query=703&hit=1&index=gene_42|conserved&px=1&section=5&ave_thresh=3&_ignoreionsscorebelow=20&report=0&_sigthreshold=0.05&_msresflags=1089&_msresflags2=2&percolate=-1&percolate_rt=0))

**393 - 408 903.9589 1805.9032 1805.9022 1 0 K.WVAQMLGLPDSGIEFK.L**  Oxidation (M) ([Ions score 89](http://gary/mascot/cgi/peptide_view.pl?file=../data/20130722/F001267.dat&query=708&hit=1&index=gene_42|conserved&px=1&section=5&ave_thresh=3&_ignoreionsscorebelow=20&report=0&_sigthreshold=0.05&_msresflags=1089&_msresflags2=2&percolate=-1&percolate_rt=0))

**446 - 458 517.5758 1549.7056 1549.7096 -3 1 R.RGGWTNWTNAEMK.E**  ([Ions score 23](http://gary/mascot/cgi/peptide_view.pl?file=../data/20130722/F001267.dat&query=625&hit=1&index=gene_42|conserved&px=1&section=5&ave_thresh=3&_ignoreionsscorebelow=20&report=0&_sigthreshold=0.05&_msresflags=1089&_msresflags2=2&percolate=-1&percolate_rt=0))

**447 - 458 697.8116 1393.6086 1393.6085 0 0 R.GGWTNWTNAEMK.E**  ([Ions score 60](http://gary/mascot/cgi/peptide_view.pl?file=../data/20130722/F001267.dat&query=556&hit=1&index=gene_42|conserved&px=1&section=5&ave_thresh=3&_ignoreionsscorebelow=20&report=0&_sigthreshold=0.05&_msresflags=1089&_msresflags2=2&percolate=-1&percolate_rt=0))

**447 - 458 705.8094 1409.6042 1409.6034 1 0 R.GGWTNWTNAEMK.E**  Oxidation (M) ([Ions score 73](http://gary/mascot/cgi/peptide_view.pl?file=../data/20130722/F001267.dat&query=560&hit=1&index=gene_42|conserved&px=1&section=5&ave_thresh=3&_ignoreionsscorebelow=20&report=0&_sigthreshold=0.05&_msresflags=1089&_msresflags2=2&percolate=-1&percolate_rt=0))

**447 - 460 826.3774 1650.7402 1650.7460 -4 1 R.GGWTNWTNAEMKEK.I**  ([Ions score 67](http://gary/mascot/cgi/peptide_view.pl?file=../data/20130722/F001267.dat&query=663&hit=1&index=gene_42|conserved&px=1&section=5&ave_thresh=3&_ignoreionsscorebelow=20&report=0&_sigthreshold=0.05&_msresflags=1089&_msresflags2=2&percolate=-1&percolate_rt=0))

**447 - 460 834.3752 1666.7358 1666.7409 -3 1 R.GGWTNWTNAEMKEK.I**  Oxidation (M) ([Ions score 70](http://gary/mascot/cgi/peptide_view.pl?file=../data/20130722/F001267.dat&query=671&hit=1&index=gene_42|conserved&px=1&section=5&ave_thresh=3&_ignoreionsscorebelow=20&report=0&_sigthreshold=0.05&_msresflags=1089&_msresflags2=2&percolate=-1&percolate_rt=0))

**459 - 500 868.8241 4339.0841 4339.0952 -3 1 K.EKIEAAPVPSISAATPVAGDIPASAQQPQADNQQQNDQQAQQ.-**([Ions score 57](http://gary/mascot/cgi/peptide_view.pl?file=../data/20130722/F001267.dat&query=971&hit=1&index=gene_42|conserved&px=1&section=5&ave_thresh=3&_ignoreionsscorebelow=20&report=0&_sigthreshold=0.05&_msresflags=1089&_msresflags2=2&percolate=-1&percolate_rt=0))

**459 - 500 1085.7791 4339.0871 4339.0952 -2 1 K.EKIEAAPVPSISAATPVAGDIPASAQQPQADNQQQNDQQAQQ.-**([Ions score 80](http://gary/mascot/cgi/peptide_view.pl?file=../data/20130722/F001267.dat&query=972&hit=1&index=gene_42|conserved&px=1&section=5&ave_thresh=3&_ignoreionsscorebelow=20&report=0&_sigthreshold=0.05&_msresflags=1089&_msresflags2=2&percolate=-1&percolate_rt=0))

**459 - 500 724.1932 4339.1154 4339.0952 5 1 K.EKIEAAPVPSISAATPVAGDIPASAQQPQADNQQQNDQQAQQ.-**([Ions score 31](http://gary/mascot/cgi/peptide_view.pl?file=../data/20130722/F001267.dat&query=973&hit=1&index=gene_42|conserved&px=1&section=5&ave_thresh=3&_ignoreionsscorebelow=20&report=0&_sigthreshold=0.05&_msresflags=1089&_msresflags2=2&percolate=-1&percolate_rt=0))

**461 - 500 1021.4966 4081.9575 4081.9577 0 0 K.IEAAPVPSISAATPVAGDIPASAQQPQADNQQQNDQQAQQ.-**  ([Ions score 89](http://gary/mascot/cgi/peptide_view.pl?file=../data/20130722/F001267.dat&query=967&hit=1&index=gene_42|conserved&px=1&section=5&ave_thresh=3&_ignoreionsscorebelow=20&report=0&_sigthreshold=0.05&_msresflags=1089&_msresflags2=2&percolate=-1&percolate_rt=0))

**5.** [gene_69|putative](http://10.139.25.109/mascot/cgi/protein_view.pl?file=../data/20100316/F010067.dat&hit=gene_69|conserved&px=1&ave_thresh=1&_sigthreshold=0.05&_server_mudpit_switch=1e-009&_ignoreionsscorebelow=20) tailspike protein|[Serratia phage Eta]; **Mass:** 76109    **Score:** 4151;    **Queries matched:** 74(74);   **emPAI:** 13.26; Sequence Coverage: **49%** ; Matched peptides shown in **Bold Red**

**1** M**SSGCGDVLS LEDLKTAKKH QLFEAEVITG RSGGVATGAF IDIATNQATG QVQKTMPAVL RDIGFAPASF DFNTGGTISD RNVAVLWPLP GGDGDWYVWE**

**101 GALPKIIPAA STPDSTGGVA AGAWK**AVNSN ALRDQLSVVS PEMFGAIGNG IADDTIAWQA MDSYVSAR**AT ASHIITIIAS R**KYRVTAQLN GWSYVNLLGG

**201** GTLIYDGADQ LNSTVLTYNS K**TKFNISNIN ITRNFNDPFA LRSK**GAYGLT LASCSDFSVV GCDIYMHTDA LSVMDSDRFK **IESNRTHELG EEGIAVRRSR**

**301 NWSVINNDVY HHNGDGILIK TGNVSSYAGS ICDNRVYDGI QSAGTAGGNR GGGITGNDEV IGGGATESFN RLIVER**NHCY NVSYGIAFTN IVDLSMSNNM

**401** VNNIDR**FGII IDTALFNNPN K**NPVKRISVS NNHVSGTVQA GLLFTGTADI SVSQVTISNN IVDTCGTSTA AGYPAIGASF ASVTGNKVTN CKIGLQVEGC

**501** ATTGNVITDS TYTSSAPASV WVK**IIGGGSF NGNSLSDSKF GHIR**FSSISG LTFTGNTITT ASSFACLYFD SVSSGSTCIF RANNYVSAFT NVSVFNIGAV

**601** PAVPALVGDE LIVSSPPAGG IRR**LVCVTAG NPGVYVPVEW NIISAVSSSS SPTINAGASL RITGTAAGVA SNSACVGAK**F NQDPQGVDMV ASVTGAGTVT

**701** FILTNNTGSS K**TFSNLVMTA YCHS**

**Start - End Observed Mr(expt) Mr(calc) ppm Miss Sequence**

**2 - 15 493.9045 1478.6918 1478.6923 0 0 M.SSGCGDVLSLEDLK.T**  ([Ions score 29](http://gary/mascot/cgi/peptide_view.pl?file=../data/20130722/F001269.dat&query=605&hit=1&index=gene_69|conserved&px=1&section=5&ave_thresh=2&_ignoreionsscorebelow=20&report=0&_sigthreshold=0.05&_msresflags=1089&_msresflags2=2&percolate=-1&percolate_rt=0))

**2 - 15 740.3546 1478.6945 1478.6923 2 0 M.SSGCGDVLSLEDLK.T**  ([Ions score 88](http://gary/mascot/cgi/peptide_view.pl?file=../data/20130722/F001269.dat&query=606&hit=1&index=gene_69|conserved&px=1&section=5&ave_thresh=2&_ignoreionsscorebelow=20&report=0&_sigthreshold=0.05&_msresflags=1089&_msresflags2=2&percolate=-1&percolate_rt=0))

**2 - 15 761.3584 1520.7022 1520.7028 0 0 M.SSGCGDVLSLEDLK.T**  Acetyl (Protein N-term) ([Ions score 61](http://gary/mascot/cgi/peptide_view.pl?file=../data/20130722/F001269.dat&query=634&hit=1&index=gene_69|conserved&px=1&section=5&ave_thresh=2&_ignoreionsscorebelow=20&report=0&_sigthreshold=0.05&_msresflags=1089&_msresflags2=2&percolate=-1&percolate_rt=0))

**2 - 18 911.4507 1820.8868 1820.8826 2 1 M.SSGCGDVLSLEDLKTAK.K**  Acetyl (Protein N-term) ([Ions score 104](http://gary/mascot/cgi/peptide_view.pl?file=../data/20130722/F001269.dat&query=774&hit=1&index=gene_69|conserved&px=1&section=5&ave_thresh=2&_ignoreionsscorebelow=20&report=0&_sigthreshold=0.05&_msresflags=1089&_msresflags2=2&percolate=-1&percolate_rt=0))

**19 - 31 509.9456 1526.8149 1526.8205 -4 1 K.KHQLFEAEVITGR.S**  ([Ions score 48](http://gary/mascot/cgi/peptide_view.pl?file=../data/20130722/F001269.dat&query=635&hit=1&index=gene_69|conserved&px=1&section=5&ave_thresh=2&_ignoreionsscorebelow=20&report=0&_sigthreshold=0.05&_msresflags=1089&_msresflags2=2&percolate=-1&percolate_rt=0))

**19 - 31 509.9463 1526.8171 1526.8205 -2 1 K.KHQLFEAEVITGR.S**  ([Ions score 60](http://gary/mascot/cgi/peptide_view.pl?file=../data/20130722/F001269.dat&query=636&hit=1&index=gene_69|conserved&px=1&section=5&ave_thresh=2&_ignoreionsscorebelow=20&report=0&_sigthreshold=0.05&_msresflags=1089&_msresflags2=2&percolate=-1&percolate_rt=0))

**19 - 31 509.9464 1526.8175 1526.8205 -2 1 K.KHQLFEAEVITGR.S**  ([Ions score 49](http://gary/mascot/cgi/peptide_view.pl?file=../data/20130722/F001269.dat&query=637&hit=1&index=gene_69|conserved&px=1&section=5&ave_thresh=2&_ignoreionsscorebelow=20&report=0&_sigthreshold=0.05&_msresflags=1089&_msresflags2=2&percolate=-1&percolate_rt=0))

**19 - 31 509.9468 1526.8186 1526.8205 -1 1 K.KHQLFEAEVITGR.S**  ([Ions score 42](http://gary/mascot/cgi/peptide_view.pl?file=../data/20130722/F001269.dat&query=638&hit=1&index=gene_69|conserved&px=1&section=5&ave_thresh=2&_ignoreionsscorebelow=20&report=0&_sigthreshold=0.05&_msresflags=1089&_msresflags2=2&percolate=-1&percolate_rt=0))

**19 - 31 509.9469 1526.8190 1526.8205 -1 1 K.KHQLFEAEVITGR.S**  ([Ions score 58](http://gary/mascot/cgi/peptide_view.pl?file=../data/20130722/F001269.dat&query=639&hit=1&index=gene_69|conserved&px=1&section=5&ave_thresh=2&_ignoreionsscorebelow=20&report=0&_sigthreshold=0.05&_msresflags=1089&_msresflags2=2&percolate=-1&percolate_rt=0))

**19 - 31 509.9475 1526.8207 1526.8205 0 1 K.KHQLFEAEVITGR.S**  ([Ions score 54](http://gary/mascot/cgi/peptide_view.pl?file=../data/20130722/F001269.dat&query=640&hit=1&index=gene_69|conserved&px=1&section=5&ave_thresh=2&_ignoreionsscorebelow=20&report=0&_sigthreshold=0.05&_msresflags=1089&_msresflags2=2&percolate=-1&percolate_rt=0))

**19 - 31 509.9475 1526.8208 1526.8205 0 1 K.KHQLFEAEVITGR.S**  ([Ions score 56](http://gary/mascot/cgi/peptide_view.pl?file=../data/20130722/F001269.dat&query=641&hit=1&index=gene_69|conserved&px=1&section=5&ave_thresh=2&_ignoreionsscorebelow=20&report=0&_sigthreshold=0.05&_msresflags=1089&_msresflags2=2&percolate=-1&percolate_rt=0))

**19 - 31 509.9489 1526.8248 1526.8205 3 1 K.KHQLFEAEVITGR.S**  ([Ions score 55](http://gary/mascot/cgi/peptide_view.pl?file=../data/20130722/F001269.dat&query=642&hit=1&index=gene_69|conserved&px=1&section=5&ave_thresh=2&_ignoreionsscorebelow=20&report=0&_sigthreshold=0.05&_msresflags=1089&_msresflags2=2&percolate=-1&percolate_rt=0))

**20 - 31 467.2490 1398.7251 1398.7256 0 0 K.HQLFEAEVITGR.S**  ([Ions score 24](http://gary/mascot/cgi/peptide_view.pl?file=../data/20130722/F001269.dat&query=556&hit=1&index=gene_69|conserved&px=1&section=5&ave_thresh=2&_ignoreionsscorebelow=20&report=0&_sigthreshold=0.05&_msresflags=1089&_msresflags2=2&percolate=-1&percolate_rt=0))

**32 - 54 1117.5728 2233.1309 2233.1339 -1 0 R.SGGVATGAFIDIATNQATGQVQK.T**  ([Ions score 134](http://gary/mascot/cgi/peptide_view.pl?file=../data/20130722/F001269.dat&query=883&hit=1&index=gene_69|conserved&px=1&section=5&ave_thresh=2&_ignoreionsscorebelow=20&report=0&_sigthreshold=0.05&_msresflags=1089&_msresflags2=2&percolate=-1&percolate_rt=0))

**32 - 54 745.3848 2233.1327 2233.1339 -1 0 R.SGGVATGAFIDIATNQATGQVQK.T**  ([Ions score 94](http://gary/mascot/cgi/peptide_view.pl?file=../data/20130722/F001269.dat&query=884&hit=1&index=gene_69|conserved&px=1&section=5&ave_thresh=2&_ignoreionsscorebelow=20&report=0&_sigthreshold=0.05&_msresflags=1089&_msresflags2=2&percolate=-1&percolate_rt=0))

**32 - 61 1001.5223 3001.5450 3001.5655 -7 1 R.SGGVATGAFIDIATNQATGQVQKTMPAVLR.D**  ([Ions score 145](http://gary/mascot/cgi/peptide_view.pl?file=../data/20130722/F001269.dat&query=1006&hit=1&index=gene_69|conserved&px=1&section=5&ave_thresh=2&_ignoreionsscorebelow=20&report=0&_sigthreshold=0.05&_msresflags=1089&_msresflags2=2&percolate=-1&percolate_rt=0))

**32 - 61 1006.8594 3017.5565 3017.5604 -1 1 R.SGGVATGAFIDIATNQATGQVQKTMPAVLR.D**

Oxidation (M) ([Ions score 144](http://gary/mascot/cgi/peptide_view.pl?file=../data/20130722/F001269.dat&query=1007&hit=1&index=gene_69|conserved&px=1&section=5&ave_thresh=2&_ignoreionsscorebelow=20&report=0&_sigthreshold=0.05&_msresflags=1089&_msresflags2=2&percolate=-1&percolate_rt=0))

**55 - 61 402.2256 802.4366 802.4371 -1 0 K.TMPAVLR.D**  Oxidation (M) ([Ions score 32](http://gary/mascot/cgi/peptide_view.pl?file=../data/20130722/F001269.dat&query=56&hit=1&index=gene_69|conserved&px=1&section=5&ave_thresh=2&_ignoreionsscorebelow=20&report=0&_sigthreshold=0.05&_msresflags=1089&_msresflags2=2&percolate=-1&percolate_rt=0))

**62 - 81 1044.4794 2086.9442 2086.9596 -7 0 R.DIGFAPASFDFNTGGTISDR.N**  ([Ions score 133](http://gary/mascot/cgi/peptide_view.pl?file=../data/20130722/F001269.dat&query=864&hit=1&index=gene_69|conserved&px=1&section=5&ave_thresh=2&_ignoreionsscorebelow=20&report=0&_sigthreshold=0.05&_msresflags=1089&_msresflags2=2&percolate=-1&percolate_rt=0))

**62 - 81 696.6597 2086.9572 2086.9596 -1 0 R.DIGFAPASFDFNTGGTISDR.N**  ([Ions score 57](http://gary/mascot/cgi/peptide_view.pl?file=../data/20130722/F001269.dat&query=865&hit=1&index=gene_69|conserved&px=1&section=5&ave_thresh=2&_ignoreionsscorebelow=20&report=0&_sigthreshold=0.05&_msresflags=1089&_msresflags2=2&percolate=-1&percolate_rt=0))

**82 - 105 880.4497 2638.3271 2638.3220 2 0 R.NVAVLWPLPGGDGDWYVWEGALPK.I**  ([Ions score 55](http://gary/mascot/cgi/peptide_view.pl?file=../data/20130722/F001269.dat&query=967&hit=1&index=gene_69|conserved&px=1&section=5&ave_thresh=2&_ignoreionsscorebelow=20&report=0&_sigthreshold=0.05&_msresflags=1089&_msresflags2=2&percolate=-1&percolate_rt=0))

**106 - 125 935.4880 1868.9614 1868.9632 -1 0 K.IIPAASTPDSTGGVAAGAWK.A**  ([Ions score 108](http://gary/mascot/cgi/peptide_view.pl?file=../data/20130722/F001269.dat&query=793&hit=1&index=gene_69|conserved&px=1&section=5&ave_thresh=2&_ignoreionsscorebelow=20&report=0&_sigthreshold=0.05&_msresflags=1089&_msresflags2=2&percolate=-1&percolate_rt=0))

**106 - 125 623.9951 1868.9634 1868.9632 0 0 K.IIPAASTPDSTGGVAAGAWK.A**  ([Ions score 60](http://gary/mascot/cgi/peptide_view.pl?file=../data/20130722/F001269.dat&query=794&hit=1&index=gene_69|conserved&px=1&section=5&ave_thresh=2&_ignoreionsscorebelow=20&report=0&_sigthreshold=0.05&_msresflags=1089&_msresflags2=2&percolate=-1&percolate_rt=0))

**169 - 181 677.3966 1352.7787 1352.7776 1 0 R.ATASHIITIIASR.K**  ([Ions score 73](http://gary/mascot/cgi/peptide_view.pl?file=../data/20130722/F001269.dat&query=535&hit=1&index=gene_69|conserved&px=1&section=5&ave_thresh=2&_ignoreionsscorebelow=20&report=0&_sigthreshold=0.05&_msresflags=1089&_msresflags2=2&percolate=-1&percolate_rt=0))

**222 - 233 474.2684 1419.7833 1419.7834 0 1 K.TKFNISNINITR.N**  ([Ions score 32](http://gary/mascot/cgi/peptide_view.pl?file=../data/20130722/F001269.dat&query=568&hit=1&index=gene_69|conserved&px=1&section=5&ave_thresh=2&_ignoreionsscorebelow=20&report=0&_sigthreshold=0.05&_msresflags=1089&_msresflags2=2&percolate=-1&percolate_rt=0))

**224 - 233 596.3279 1190.6413 1190.6407 0 0 K.FNISNINITR.N**  ([Ions score 69](http://gary/mascot/cgi/peptide_view.pl?file=../data/20130722/F001269.dat&query=386&hit=1&index=gene_69|conserved&px=1&section=5&ave_thresh=2&_ignoreionsscorebelow=20&report=0&_sigthreshold=0.05&_msresflags=1089&_msresflags2=2&percolate=-1&percolate_rt=0))

**224 - 233 397.8880 1190.6421 1190.6407 1 0 K.FNISNINITR.N**  ([Ions score 29](http://gary/mascot/cgi/peptide_view.pl?file=../data/20130722/F001269.dat&query=387&hit=1&index=gene_69|conserved&px=1&section=5&ave_thresh=2&_ignoreionsscorebelow=20&report=0&_sigthreshold=0.05&_msresflags=1089&_msresflags2=2&percolate=-1&percolate_rt=0))

**224 - 233 596.8193 1191.6240 1191.6248 -1 0 K.FNISNINITR.N**  Deamidated (NQ) ([Ions score 70](http://gary/mascot/cgi/peptide_view.pl?file=../data/20130722/F001269.dat&query=388&hit=1&index=gene_69|conserved&px=1&section=5&ave_thresh=2&_ignoreionsscorebelow=20&report=0&_sigthreshold=0.05&_msresflags=1089&_msresflags2=2&percolate=-1&percolate_rt=0))

**224 - 242 756.0619 2265.1638 2265.1654 -1 1 K.FNISNINITRNFNDPFALR.S**  ([Ions score 57](http://gary/mascot/cgi/peptide_view.pl?file=../data/20130722/F001269.dat&query=887&hit=1&index=gene_69|conserved&px=1&section=5&ave_thresh=2&_ignoreionsscorebelow=20&report=0&_sigthreshold=0.05&_msresflags=1089&_msresflags2=2&percolate=-1&percolate_rt=0))

**234 - 242 547.2732 1092.5318 1092.5352 -3 0 R.NFNDPFALR.S**  ([Ions score 41](http://gary/mascot/cgi/peptide_view.pl?file=../data/20130722/F001269.dat&query=271&hit=1&index=gene_69|conserved&px=1&section=5&ave_thresh=2&_ignoreionsscorebelow=20&report=0&_sigthreshold=0.05&_msresflags=1089&_msresflags2=2&percolate=-1&percolate_rt=0))

**234 - 242 547.7672 1093.5197 1093.5192 0 0 R.NFNDPFALR.S**  Deamidated (NQ) ([Ions score 58](http://gary/mascot/cgi/peptide_view.pl?file=../data/20130722/F001269.dat&query=272&hit=1&index=gene_69|conserved&px=1&section=5&ave_thresh=2&_ignoreionsscorebelow=20&report=0&_sigthreshold=0.05&_msresflags=1089&_msresflags2=2&percolate=-1&percolate_rt=0))

**234 - 244 654.8377 1307.6609 1307.6622 -1 1 R.NFNDPFALRSK.G**  ([Ions score 63](http://gary/mascot/cgi/peptide_view.pl?file=../data/20130722/F001269.dat&query=504&hit=1&index=gene_69|conserved&px=1&section=5&ave_thresh=2&_ignoreionsscorebelow=20&report=0&_sigthreshold=0.05&_msresflags=1089&_msresflags2=2&percolate=-1&percolate_rt=0))

**234 - 244 436.8945 1307.6618 1307.6622 0 1 R.NFNDPFALRSK.G**  ([Ions score 22](http://gary/mascot/cgi/peptide_view.pl?file=../data/20130722/F001269.dat&query=505&hit=1&index=gene_69|conserved&px=1&section=5&ave_thresh=2&_ignoreionsscorebelow=20&report=0&_sigthreshold=0.05&_msresflags=1089&_msresflags2=2&percolate=-1&percolate_rt=0))

**281 - 297 478.2480 1908.9629 1908.9653 -1 1 K.IESNRTHELGEEGIAVR.R**  ([Ions score 23](http://gary/mascot/cgi/peptide_view.pl?file=../data/20130722/F001269.dat&query=809&hit=1&index=gene_69|conserved&px=1&section=5&ave_thresh=2&_ignoreionsscorebelow=20&report=0&_sigthreshold=0.05&_msresflags=1089&_msresflags2=2&percolate=-1&percolate_rt=0))

**281 - 297 637.3300 1908.9681 1908.9653 1 1 K.IESNRTHELGEEGIAVR.R**  ([Ions score 43](http://gary/mascot/cgi/peptide_view.pl?file=../data/20130722/F001269.dat&query=810&hit=1&index=gene_69|conserved&px=1&section=5&ave_thresh=2&_ignoreionsscorebelow=20&report=0&_sigthreshold=0.05&_msresflags=1089&_msresflags2=2&percolate=-1&percolate_rt=0))

**281 - 298 517.2732 2065.0637 2065.0664 -1 2 K.IESNRTHELGEEGIAVRR.S**  ([Ions score 27](http://gary/mascot/cgi/peptide_view.pl?file=../data/20130722/F001269.dat&query=856&hit=1&index=gene_69|conserved&px=1&section=5&ave_thresh=2&_ignoreionsscorebelow=20&report=0&_sigthreshold=0.05&_msresflags=1089&_msresflags2=2&percolate=-1&percolate_rt=0))

**281 - 298 414.0202 2065.0646 2065.0664 -1 2 K.IESNRTHELGEEGIAVRR.S**  ([Ions score 51](http://gary/mascot/cgi/peptide_view.pl?file=../data/20130722/F001269.dat&query=857&hit=1&index=gene_69|conserved&px=1&section=5&ave_thresh=2&_ignoreionsscorebelow=20&report=0&_sigthreshold=0.05&_msresflags=1089&_msresflags2=2&percolate=-1&percolate_rt=0))

**281 - 298 689.3624 2065.0653 2065.0664 -1 2 K.IESNRTHELGEEGIAVRR.S**  ([Ions score 33](http://gary/mascot/cgi/peptide_view.pl?file=../data/20130722/F001269.dat&query=858&hit=1&index=gene_69|conserved&px=1&section=5&ave_thresh=2&_ignoreionsscorebelow=20&report=0&_sigthreshold=0.05&_msresflags=1089&_msresflags2=2&percolate=-1&percolate_rt=0))

**286 - 297 655.8358 1309.6571 1309.6626 -4 0 R.THELGEEGIAVR.R**  ([Ions score 65](http://gary/mascot/cgi/peptide_view.pl?file=../data/20130722/F001269.dat&query=506&hit=1&index=gene_69|conserved&px=1&section=5&ave_thresh=2&_ignoreionsscorebelow=20&report=0&_sigthreshold=0.05&_msresflags=1089&_msresflags2=2&percolate=-1&percolate_rt=0))

**286 - 297 437.5615 1309.6626 1309.6626 0 0 R.THELGEEGIAVR.R**  ([Ions score 34](http://gary/mascot/cgi/peptide_view.pl?file=../data/20130722/F001269.dat&query=507&hit=1&index=gene_69|conserved&px=1&section=5&ave_thresh=2&_ignoreionsscorebelow=20&report=0&_sigthreshold=0.05&_msresflags=1089&_msresflags2=2&percolate=-1&percolate_rt=0))

**286 - 298 489.5944 1465.7613 1465.7637 -2 1 R.THELGEEGIAVRR.S**  ([Ions score 26](http://gary/mascot/cgi/peptide_view.pl?file=../data/20130722/F001269.dat&query=594&hit=1&index=gene_69|conserved&px=1&section=5&ave_thresh=2&_ignoreionsscorebelow=20&report=0&_sigthreshold=0.05&_msresflags=1089&_msresflags2=2&percolate=-1&percolate_rt=0))

**286 - 298 489.5951 1465.7635 1465.7637 0 1 R.THELGEEGIAVRR.S**  ([Ions score 50](http://gary/mascot/cgi/peptide_view.pl?file=../data/20130722/F001269.dat&query=595&hit=1&index=gene_69|conserved&px=1&section=5&ave_thresh=2&_ignoreionsscorebelow=20&report=0&_sigthreshold=0.05&_msresflags=1089&_msresflags2=2&percolate=-1&percolate_rt=0))

**299 - 320 638.5764 2550.2763 2550.2728 1 1 R.SRNWSVINNDVYHHNGDGILIK.T**  ([Ions score 45](http://gary/mascot/cgi/peptide_view.pl?file=../data/20130722/F001269.dat&query=945&hit=1&index=gene_69|conserved&px=1&section=5&ave_thresh=2&_ignoreionsscorebelow=20&report=0&_sigthreshold=0.05&_msresflags=1089&_msresflags2=2&percolate=-1&percolate_rt=0))

**299 - 320 638.8219 2551.2585 2551.2568 1 1 R.SRNWSVINNDVYHHNGDGILIK.T**  Deamidated (NQ) ([Ions score 44](http://gary/mascot/cgi/peptide_view.pl?file=../data/20130722/F001269.dat&query=948&hit=1&index=gene_69|conserved&px=1&section=5&ave_thresh=2&_ignoreionsscorebelow=20&report=0&_sigthreshold=0.05&_msresflags=1089&_msresflags2=2&percolate=-1&percolate_rt=0))

**301 - 320 770.0534 2307.1384 2307.1396 -1 0 R.NWSVINNDVYHHNGDGILIK.T**  ([Ions score 47](http://gary/mascot/cgi/peptide_view.pl?file=../data/20130722/F001269.dat&query=896&hit=1&index=gene_69|conserved&px=1&section=5&ave_thresh=2&_ignoreionsscorebelow=20&report=0&_sigthreshold=0.05&_msresflags=1089&_msresflags2=2&percolate=-1&percolate_rt=0))

**301 - 320 770.3858 2308.1356 2308.1236 5 0 R.NWSVINNDVYHHNGDGILIK.T**  Deamidated (NQ) ([Ions score 50](http://gary/mascot/cgi/peptide_view.pl?file=../data/20130722/F001269.dat&query=899&hit=1&index=gene_69|conserved&px=1&section=5&ave_thresh=2&_ignoreionsscorebelow=20&report=0&_sigthreshold=0.05&_msresflags=1089&_msresflags2=2&percolate=-1&percolate_rt=0))

**321 - 335 800.8539 1599.6932 1599.6947 -1 0 K.TGNVSSYAGSICDNR.V**  ([Ions score 92](http://gary/mascot/cgi/peptide_view.pl?file=../data/20130722/F001269.dat&query=701&hit=1&index=gene_69|conserved&px=1&section=5&ave_thresh=2&_ignoreionsscorebelow=20&report=0&_sigthreshold=0.05&_msresflags=1089&_msresflags2=2&percolate=-1&percolate_rt=0))

**321 - 335 534.2393 1599.6961 1599.6947 1 0 K.TGNVSSYAGSICDNR.V**  ([Ions score 59](http://gary/mascot/cgi/peptide_view.pl?file=../data/20130722/F001269.dat&query=702&hit=1&index=gene_69|conserved&px=1&section=5&ave_thresh=2&_ignoreionsscorebelow=20&report=0&_sigthreshold=0.05&_msresflags=1089&_msresflags2=2&percolate=-1&percolate_rt=0))

**321 - 350 1016.4657 3046.3753 3046.3799 -2 1 K.TGNVSSYAGSICDNRVYDGIQSAGTAGGNR.G**  ([Ions score 120](http://gary/mascot/cgi/peptide_view.pl?file=../data/20130722/F001269.dat&query=1015&hit=1&index=gene_69|conserved&px=1&section=5&ave_thresh=2&_ignoreionsscorebelow=20&report=0&_sigthreshold=0.05&_msresflags=1089&_msresflags2=2&percolate=-1&percolate_rt=0))

**321 - 350 762.6019 3046.3784 3046.3799 0 1 K.TGNVSSYAGSICDNRVYDGIQSAGTAGGNR.G**  ([Ions score 39](http://gary/mascot/cgi/peptide_view.pl?file=../data/20130722/F001269.dat&query=1016&hit=1&index=gene_69|conserved&px=1&section=5&ave_thresh=2&_ignoreionsscorebelow=20&report=0&_sigthreshold=0.05&_msresflags=1089&_msresflags2=2&percolate=-1&percolate_rt=0))

**321 - 350 1016.4697 3046.3872 3046.3799 2 1 K.TGNVSSYAGSICDNRVYDGIQSAGTAGGNR.G**  ([Ions score 117](http://gary/mascot/cgi/peptide_view.pl?file=../data/20130722/F001269.dat&query=1017&hit=1&index=gene_69|conserved&px=1&section=5&ave_thresh=2&_ignoreionsscorebelow=20&report=0&_sigthreshold=0.05&_msresflags=1089&_msresflags2=2&percolate=-1&percolate_rt=0))

**336 - 350 489.2394 1464.6965 1464.6957 1 0 R.VYDGIQSAGTAGGNR.G**  ([Ions score 59](http://gary/mascot/cgi/peptide_view.pl?file=../data/20130722/F001269.dat&query=592&hit=1&index=gene_69|conserved&px=1&section=5&ave_thresh=2&_ignoreionsscorebelow=20&report=0&_sigthreshold=0.05&_msresflags=1089&_msresflags2=2&percolate=-1&percolate_rt=0))

**336 - 350 733.3563 1464.6980 1464.6957 2 0 R.VYDGIQSAGTAGGNR.G**  ([Ions score 102](http://gary/mascot/cgi/peptide_view.pl?file=../data/20130722/F001269.dat&query=593&hit=1&index=gene_69|conserved&px=1&section=5&ave_thresh=2&_ignoreionsscorebelow=20&report=0&_sigthreshold=0.05&_msresflags=1089&_msresflags2=2&percolate=-1&percolate_rt=0))

**336 - 371 864.4105 3453.6127 3453.6145 -1 1 R.VYDGIQSAGTAGGNRGGGITGNDEVIGGGATESFNR.L**  ([Ions score 58](http://gary/mascot/cgi/peptide_view.pl?file=../data/20130722/F001269.dat&query=1030&hit=1&index=gene_69|conserved&px=1&section=5&ave_thresh=2&_ignoreionsscorebelow=20&report=0&_sigthreshold=0.05&_msresflags=1089&_msresflags2=2&percolate=-1&percolate_rt=0))

**351 - 371 669.9835 2006.9287 2006.9294 0 0 R.GGGITGNDEVIGGGATESFNR.L**  ([Ions score 82](http://gary/mascot/cgi/peptide_view.pl?file=../data/20130722/F001269.dat&query=837&hit=1&index=gene_69|conserved&px=1&section=5&ave_thresh=2&_ignoreionsscorebelow=20&report=0&_sigthreshold=0.05&_msresflags=1089&_msresflags2=2&percolate=-1&percolate_rt=0))

**351 - 371 1004.4717 2006.9288 2006.9294 0 0 R.GGGITGNDEVIGGGATESFNR.L**  ([Ions score 144](http://gary/mascot/cgi/peptide_view.pl?file=../data/20130722/F001269.dat&query=838&hit=1&index=gene_69|conserved&px=1&section=5&ave_thresh=2&_ignoreionsscorebelow=20&report=0&_sigthreshold=0.05&_msresflags=1089&_msresflags2=2&percolate=-1&percolate_rt=0))

**351 - 371 1004.9673 2007.9200 2007.9134 3 0 R.GGGITGNDEVIGGGATESFNR.L**  Deamidated (NQ) ([Ions score 137](http://gary/mascot/cgi/peptide_view.pl?file=../data/20130722/F001269.dat&query=839&hit=1&index=gene_69|conserved&px=1&section=5&ave_thresh=2&_ignoreionsscorebelow=20&report=0&_sigthreshold=0.05&_msresflags=1089&_msresflags2=2&percolate=-1&percolate_rt=0))

**351 - 371 670.3146 2007.9219 2007.9134 4 0 R.GGGITGNDEVIGGGATESFNR.L**  Deamidated (NQ) ([Ions score 87](http://gary/mascot/cgi/peptide_view.pl?file=../data/20130722/F001269.dat&query=840&hit=1&index=gene_69|conserved&px=1&section=5&ave_thresh=2&_ignoreionsscorebelow=20&report=0&_sigthreshold=0.05&_msresflags=1089&_msresflags2=2&percolate=-1&percolate_rt=0))

**351 - 376 873.4437 2617.3092 2617.3096 0 1 R.GGGITGNDEVIGGGATESFNRLIVER.N**  ([Ions score 74](http://gary/mascot/cgi/peptide_view.pl?file=../data/20130722/F001269.dat&query=966&hit=1&index=gene_69|conserved&px=1&section=5&ave_thresh=2&_ignoreionsscorebelow=20&report=0&_sigthreshold=0.05&_msresflags=1089&_msresflags2=2&percolate=-1&percolate_rt=0))

**372 - 376 315.2025 628.3904 628.3908 -1 0 R.LIVER.N**  ([Ions score 24](http://gary/mascot/cgi/peptide_view.pl?file=../data/20130722/F001269.dat&query=5&hit=1&index=gene_69|conserved&px=1&section=5&ave_thresh=2&_ignoreionsscorebelow=20&report=0&_sigthreshold=0.05&_msresflags=1089&_msresflags2=2&percolate=-1&percolate_rt=0))

**407 - 421 838.9533 1675.8919 1675.8933 -1 0 R.FGIIIDTALFNNPNK.N**  ([Ions score 57](http://gary/mascot/cgi/peptide_view.pl?file=../data/20130722/F001269.dat&query=735&hit=1&index=gene_69|conserved&px=1&section=5&ave_thresh=2&_ignoreionsscorebelow=20&report=0&_sigthreshold=0.05&_msresflags=1089&_msresflags2=2&percolate=-1&percolate_rt=0))

**407 - 421 838.9543 1675.8941 1675.8933 0 0 R.FGIIIDTALFNNPNK.N**  ([Ions score 98](http://gary/mascot/cgi/peptide_view.pl?file=../data/20130722/F001269.dat&query=736&hit=1&index=gene_69|conserved&px=1&section=5&ave_thresh=2&_ignoreionsscorebelow=20&report=0&_sigthreshold=0.05&_msresflags=1089&_msresflags2=2&percolate=-1&percolate_rt=0))

**407 - 421 559.6387 1675.8944 1675.8933 1 0 R.FGIIIDTALFNNPNK.N**  ([Ions score 72](http://gary/mascot/cgi/peptide_view.pl?file=../data/20130722/F001269.dat&query=737&hit=1&index=gene_69|conserved&px=1&section=5&ave_thresh=2&_ignoreionsscorebelow=20&report=0&_sigthreshold=0.05&_msresflags=1089&_msresflags2=2&percolate=-1&percolate_rt=0))

**524 - 539 776.8836 1551.7527 1551.7529 0 0 K.IIGGGSFNGNSLSDSK.F**  ([Ions score 115](http://gary/mascot/cgi/peptide_view.pl?file=../data/20130722/F001269.dat&query=665&hit=1&index=gene_69|conserved&px=1&section=5&ave_thresh=2&_ignoreionsscorebelow=20&report=0&_sigthreshold=0.05&_msresflags=1089&_msresflags2=2&percolate=-1&percolate_rt=0))

**524 - 539 777.3762 1552.7378 1552.7369 1 0 K.IIGGGSFNGNSLSDSK.F**  Deamidated (NQ) ([Ions score 94](http://gary/mascot/cgi/peptide_view.pl?file=../data/20130722/F001269.dat&query=666&hit=1&index=gene_69|conserved&px=1&section=5&ave_thresh=2&_ignoreionsscorebelow=20&report=0&_sigthreshold=0.05&_msresflags=1089&_msresflags2=2&percolate=-1&percolate_rt=0))

**540 - 544 315.1791 628.3436 628.3445 -1 0 K.FGHIR.F**  ([Ions score 25](http://gary/mascot/cgi/peptide_view.pl?file=../data/20130722/F001269.dat&query=4&hit=1&index=gene_69|conserved&px=1&section=5&ave_thresh=2&_ignoreionsscorebelow=20&report=0&_sigthreshold=0.05&_msresflags=1089&_msresflags2=2&percolate=-1&percolate_rt=0))

**624 - 661 969.0019 3871.9785 3871.9778 0 0 R.LVCVTAGNPGVYVPVEWNIISAVSSSSSPTINAGASLR.I**  ([Ions score 31](http://gary/mascot/cgi/peptide_view.pl?file=../data/20130722/F001269.dat&query=1039&hit=1&index=gene_69|conserved&px=1&section=5&ave_thresh=2&_ignoreionsscorebelow=20&report=0&_sigthreshold=0.05&_msresflags=1089&_msresflags2=2&percolate=-1&percolate_rt=0))

**624 - 661 1291.6715 3871.9927 3871.9778 4 0 R.LVCVTAGNPGVYVPVEWNIISAVSSSSSPTINAGASLR.I**  ([Ions score 117](http://gary/mascot/cgi/peptide_view.pl?file=../data/20130722/F001269.dat&query=1040&hit=1&index=gene_69|conserved&px=1&section=5&ave_thresh=2&_ignoreionsscorebelow=20&report=0&_sigthreshold=0.05&_msresflags=1089&_msresflags2=2&percolate=-1&percolate_rt=0))

**662 - 679 817.9076 1633.8006 1633.8094 -5 0 R.ITGTAAGVASNSACVGAK.F**  ([Ions score 105](http://gary/mascot/cgi/peptide_view.pl?file=../data/20130722/F001269.dat&query=713&hit=1&index=gene_69|conserved&px=1&section=5&ave_thresh=2&_ignoreionsscorebelow=20&report=0&_sigthreshold=0.05&_msresflags=1089&_msresflags2=2&percolate=-1&percolate_rt=0))

**662 - 679 817.9106 1633.8066 1633.8094 -2 0 R.ITGTAAGVASNSACVGAK.F**  ([Ions score 88](http://gary/mascot/cgi/peptide_view.pl?file=../data/20130722/F001269.dat&query=714&hit=1&index=gene_69|conserved&px=1&section=5&ave_thresh=2&_ignoreionsscorebelow=20&report=0&_sigthreshold=0.05&_msresflags=1089&_msresflags2=2&percolate=-1&percolate_rt=0))

**662 - 679 818.4062 1634.7978 1634.7934 3 0 R.ITGTAAGVASNSACVGAK.F**  Deamidated (NQ) ([Ions score 123](http://gary/mascot/cgi/peptide_view.pl?file=../data/20130722/F001269.dat&query=716&hit=1&index=gene_69|conserved&px=1&section=5&ave_thresh=2&_ignoreionsscorebelow=20&report=0&_sigthreshold=0.05&_msresflags=1089&_msresflags2=2&percolate=-1&percolate_rt=0))

**712 - 724 765.8388 1529.6631 1529.6643 -1 0 K.TFSNLVMTAYCHS.-**  ([Ions score 86](http://gary/mascot/cgi/peptide_view.pl?file=../data/20130722/F001269.dat&query=645&hit=1&index=gene_69|conserved&px=1&section=5&ave_thresh=2&_ignoreionsscorebelow=20&report=0&_sigthreshold=0.05&_msresflags=1089&_msresflags2=2&percolate=-1&percolate_rt=0))

**712 - 724 773.8381 1545.6617 1545.6592 2 0 K.TFSNLVMTAYCHS.-**  Oxidation (M) ([Ions score 84](http://gary/mascot/cgi/peptide_view.pl?file=../data/20130722/F001269.dat&query=660&hit=1&index=gene_69|conserved&px=1&section=5&ave_thresh=2&_ignoreionsscorebelow=20&report=0&_sigthreshold=0.05&_msresflags=1089&_msresflags2=2&percolate=-1&percolate_rt=0))

**6.**[gene_63|putative](http://10.139.25.109/mascot/cgi/protein_view.pl?file=../data/20100316/F010068.dat&hit=gene_63|putative&px=1&ave_thresh=1&_sigthreshold=0.05&_server_mudpit_switch=1e-009&_ignoreionsscorebelow=20) tail tape measure protein|[Serratia phage Eta]; **Mass:** 78127    **Score:** 4167; **Queries matched:** 81(81);   **emPAI:** 25.83; Sequence Coverage: **60%;** Matched peptides shown in **Bold Red**

**1** M**ADTASLVAR** VKTEGAK**QAA DELNNVAGAA NNADAAVQKI PKDANAAGQA MGNAAKGGFS SFK**NSAQQVG FQVQDMVVQL QSGTSAFVAI GQQGSQLAGA

**101** FGPGGAVLGA VIALASAVGG VLYKSMGNAK **VTAAEMEEAH KTLAGVLKQT SSGAYEVSDA IQTMALSGAS AAEIDAKFAD AQEALRTK**VE ASTQAVVEAT

**201** K**ATDTWLYGT AVGAEHNLRL GDSAGTAAGF IKDLSSSLGI TSDEATRLVP LLAAVQKEST PANVAALR**AE VER**LTTAHGT ENDELRKLNS TLQQNATDTR**

**301 NAKAADDALA ASKANLIKRI QEQNDAIVKN QQISILADRD RAK**AQAAADK **EAFAKREGVT KEQIAAFNAA RDTEAQQDIA RIDATEKAKS DRIAAAGQKR**

**401 LDTQARQAET AAARQKKAAD AFLAQVDRTS GDEIAR**ITAT EQQKLEQLNA FNQQGLIVGQ QYEQAK**TDIQ LAAEDAR**QKE LEK**RRNTQAR** QQNEHDQFIA

**501** NIQALNATEI EMIDAQNEAK LAKAKEMHDK **GRISEQEYQD SIRAIQENTE KKKEEVQLAA LSDMTSNLKT ALGEGNALYK AAAITQTIID TYKGATAAYS**

**601 AFAGIPIVGP ALGAAAAAAA IGAGMAR**VSA IRSAREQGGN LAPGQVSTIA ERGKPEVIMP AGASRVRTAQ QMRQIMGENS GGSGGDSNIV IVNQTTGRID

**701** SVQQDRDDEG RLRVIIRELV SGDLQDSNSD ISKSRRSTRG QPGH

**Start - End Observed Mr(expt) Mr(calc) ppm Miss Sequence**

**2 - 10 452.2482 902.4818 902.4821 0 0 M.ADTASLVAR.V**  ([Ions score 60](http://gary/mascot/cgi/peptide_view.pl?file=../data/20130722/F001268.dat&query=84&hit=1&index=gene_63|putative&px=1&section=5&ave_thresh=3&_ignoreionsscorebelow=20&report=0&_sigthreshold=0.05&_msresflags=1089&_msresflags2=2&percolate=-1&percolate_rt=0))

**18 - 39 1069.5054 2136.9962 2137.0036 -3 0 K.QAADELNNVAGAANNADAAVQK.I**

Gln->pyro-Glu(N-term Q)([Ions score 113](http://gary/mascot/cgi/peptide_view.pl?file=../data/20130722/F001268.dat&query=504&hit=1&index=gene_63|putative&px=1&section=5&ave_thresh=3&_ignoreionsscorebelow=20&report=0&_sigthreshold=0.05&_msresflags=1089&_msresflags2=2&percolate=-1&percolate_rt=0))

**18 - 39 713.3428 2137.0065 2137.0036 1 0 K.QAADELNNVAGAANNADAAVQK.I**

Gln->pyro-Glu (N-term Q) ([Ions score 113](http://gary/mascot/cgi/peptide_view.pl?file=../data/20130722/F001268.dat&query=505&hit=1&index=gene_63|putative&px=1&section=5&ave_thresh=3&_ignoreionsscorebelow=20&report=0&_sigthreshold=0.05&_msresflags=1089&_msresflags2=2&percolate=-1&percolate_rt=0))

**18 - 39 1078.0222 2154.0299 2154.0301 0 0 K.QAADELNNVAGAANNADAAVQK.I**  ([Ions score 141](http://gary/mascot/cgi/peptide_view.pl?file=../data/20130722/F001268.dat&query=506&hit=1&index=gene_63|putative&px=1&section=5&ave_thresh=3&_ignoreionsscorebelow=20&report=0&_sigthreshold=0.05&_msresflags=1089&_msresflags2=2&percolate=-1&percolate_rt=0))

**18 - 39 719.0176 2154.0311 2154.0301 0 0 K.QAADELNNVAGAANNADAAVQK.I**  ([Ions score 127](http://gary/mascot/cgi/peptide_view.pl?file=../data/20130722/F001268.dat&query=507&hit=1&index=gene_63|putative&px=1&section=5&ave_thresh=3&_ignoreionsscorebelow=20&report=0&_sigthreshold=0.05&_msresflags=1089&_msresflags2=2&percolate=-1&percolate_rt=0))

**18 - 39 719.3464 2155.0173 2155.0141 1 0 K.QAADELNNVAGAANNADAAVQK.I**  Deamidated (NQ) ([Ions score 120](http://gary/mascot/cgi/peptide_view.pl?file=../data/20130722/F001268.dat&query=508&hit=1&index=gene_63|putative&px=1&section=5&ave_thresh=3&_ignoreionsscorebelow=20&report=0&_sigthreshold=0.05&_msresflags=1089&_msresflags2=2&percolate=-1&percolate_rt=0))

**18 - 39 1078.5172 2155.0199 2155.0141 3 0 K.QAADELNNVAGAANNADAAVQK.I**  Deamidated (NQ) ([Ions score 134](http://gary/mascot/cgi/peptide_view.pl?file=../data/20130722/F001268.dat&query=509&hit=1&index=gene_63|putative&px=1&section=5&ave_thresh=3&_ignoreionsscorebelow=20&report=0&_sigthreshold=0.05&_msresflags=1089&_msresflags2=2&percolate=-1&percolate_rt=0))

**18 - 39 719.3484 2155.0235 2155.0141 4 0 K.QAADELNNVAGAANNADAAVQK.I**  Deamidated (NQ) ([Ions score 93](http://gary/mascot/cgi/peptide_view.pl?file=../data/20130722/F001268.dat&query=510&hit=1&index=gene_63|putative&px=1&section=5&ave_thresh=3&_ignoreionsscorebelow=20&report=0&_sigthreshold=0.05&_msresflags=1089&_msresflags2=2&percolate=-1&percolate_rt=0))

**18 - 42 831.7603 2492.2589 2492.2619 -1 1 K.QAADELNNVAGAANNADAAVQKIPK.D**  ([Ions score 134](http://gary/mascot/cgi/peptide_view.pl?file=../data/20130722/F001268.dat&query=556&hit=1&index=gene_63|putative&px=1&section=5&ave_thresh=3&_ignoreionsscorebelow=20&report=0&_sigthreshold=0.05&_msresflags=1089&_msresflags2=2&percolate=-1&percolate_rt=0))

**43 - 56 645.2967 1288.5789 1288.5830 -3 0 K.DANAAGQAMGNAAK.G**  ([Ions score 116](http://gary/mascot/cgi/peptide_view.pl?file=../data/20130722/F001268.dat&query=303&hit=1&index=gene_63|putative&px=1&section=5&ave_thresh=3&_ignoreionsscorebelow=20&report=0&_sigthreshold=0.05&_msresflags=1089&_msresflags2=2&percolate=-1&percolate_rt=0))

**43 - 56 653.2964 1304.5782 1304.5779 0 0 K.DANAAGQAMGNAAK.G**  Oxidation (M) ([Ions score 94](http://gary/mascot/cgi/peptide_view.pl?file=../data/20130722/F001268.dat&query=316&hit=1&index=gene_63|putative&px=1&section=5&ave_thresh=3&_ignoreionsscorebelow=20&report=0&_sigthreshold=0.05&_msresflags=1089&_msresflags2=2&percolate=-1&percolate_rt=0))

**57 - 63 365.1819 728.3491 728.3493 0 0 K.GGFSSFK.N**  ([Ions score 48](http://gary/mascot/cgi/peptide_view.pl?file=../data/20130722/F001268.dat&query=27&hit=1&index=gene_63|putative&px=1&section=5&ave_thresh=3&_ignoreionsscorebelow=20&report=0&_sigthreshold=0.05&_msresflags=1089&_msresflags2=2&percolate=-1&percolate_rt=0))

**131 - 141 405.8603 1214.5592 1214.5601 -1 0 K.VTAAEMEEAHK.T**  ([Ions score 29](http://gary/mascot/cgi/peptide_view.pl?file=../data/20130722/F001268.dat&query=255&hit=1&index=gene_63|putative&px=1&section=5&ave_thresh=3&_ignoreionsscorebelow=20&report=0&_sigthreshold=0.05&_msresflags=1089&_msresflags2=2&percolate=-1&percolate_rt=0))

**131 - 141 608.2870 1214.5594 1214.5601 -1 0 K.VTAAEMEEAHK.T**  ([Ions score 59](http://gary/mascot/cgi/peptide_view.pl?file=../data/20130722/F001268.dat&query=256&hit=1&index=gene_63|putative&px=1&section=5&ave_thresh=3&_ignoreionsscorebelow=20&report=0&_sigthreshold=0.05&_msresflags=1089&_msresflags2=2&percolate=-1&percolate_rt=0))

**131 - 141 411.1921 1230.5544 1230.5550 0 0 K.VTAAEMEEAHK.T**  Oxidation (M) ([Ions score 25](http://gary/mascot/cgi/peptide_view.pl?file=../data/20130722/F001268.dat&query=273&hit=1&index=gene_63|putative&px=1&section=5&ave_thresh=3&_ignoreionsscorebelow=20&report=0&_sigthreshold=0.05&_msresflags=1089&_msresflags2=2&percolate=-1&percolate_rt=0))

**131 - 141 616.2855 1230.5565 1230.5550 1 0 K.VTAAEMEEAHK.T**  Oxidation (M) ([Ions score 65](http://gary/mascot/cgi/peptide_view.pl?file=../data/20130722/F001268.dat&query=274&hit=1&index=gene_63|putative&px=1&section=5&ave_thresh=3&_ignoreionsscorebelow=20&report=0&_sigthreshold=0.05&_msresflags=1089&_msresflags2=2&percolate=-1&percolate_rt=0))

**131 - 148 475.2573 1897.0002 1896.9979 1 1 K.VTAAEMEEAHKTLAGVLK.Q**  ([Ions score 39](http://gary/mascot/cgi/peptide_view.pl?file=../data/20130722/F001268.dat&query=470&hit=1&index=gene_63|putative&px=1&section=5&ave_thresh=3&_ignoreionsscorebelow=20&report=0&_sigthreshold=0.05&_msresflags=1089&_msresflags2=2&percolate=-1&percolate_rt=0))

**142 - 148 351.2315 700.4483 700.4483 0 0 K.TLAGVLK.Q**  ([Ions score 40](http://gary/mascot/cgi/peptide_view.pl?file=../data/20130722/F001268.dat&query=19&hit=1&index=gene_63|putative&px=1&section=5&ave_thresh=3&_ignoreionsscorebelow=20&report=0&_sigthreshold=0.05&_msresflags=1089&_msresflags2=2&percolate=-1&percolate_rt=0))

**149 - 177 952.4469 2854.3189 2854.3178 0 0 K.QTSSGAYEVSDAIQTMALSGASAAEIDAK.F**

Gln->pyro-Glu (N-term Q) ([Ions score 87](http://gary/mascot/cgi/peptide_view.pl?file=../data/20130722/F001268.dat&query=595&hit=1&index=gene_63|putative&px=1&section=5&ave_thresh=3&_ignoreionsscorebelow=20&report=0&_sigthreshold=0.05&_msresflags=1089&_msresflags2=2&percolate=-1&percolate_rt=0))

**149 - 177 958.1204 2871.3393 2871.3444 -2 0 K.QTSSGAYEVSDAIQTMALSGASAAEIDAK.F**  ([Ions score 90](http://gary/mascot/cgi/peptide_view.pl?file=../data/20130722/F001268.dat&query=597&hit=1&index=gene_63|putative&px=1&section=5&ave_thresh=3&_ignoreionsscorebelow=20&report=0&_sigthreshold=0.05&_msresflags=1089&_msresflags2=2&percolate=-1&percolate_rt=0))

**149 - 177 963.4534 2887.3383 2887.3393 0 0 K.QTSSGAYEVSDAIQTMALSGASAAEIDAK.F**  Oxidation(M)([Ions score 100](http://gary/mascot/cgi/peptide_view.pl?file=../data/20130722/F001268.dat&query=602&hit=1&index=gene_63|putative&px=1&section=5&ave_thresh=3&_ignoreionsscorebelow=20&report=0&_sigthreshold=0.05&_msresflags=1089&_msresflags2=2&percolate=-1&percolate_rt=0))

**178 - 186 510.7596 1019.5047 1019.5036 1 0 K.FADAQEALR.T**  ([Ions score 62](http://gary/mascot/cgi/peptide_view.pl?file=../data/20130722/F001268.dat&query=139&hit=1&index=gene_63|putative&px=1&section=5&ave_thresh=3&_ignoreionsscorebelow=20&report=0&_sigthreshold=0.05&_msresflags=1089&_msresflags2=2&percolate=-1&percolate_rt=0))

**178 - 188 417.2234 1248.6483 1248.6462 2 1 K.FADAQEALRTK.V**  ([Ions score 32](http://gary/mascot/cgi/peptide_view.pl?file=../data/20130722/F001268.dat&query=284&hit=1&index=gene_63|putative&px=1&section=5&ave_thresh=3&_ignoreionsscorebelow=20&report=0&_sigthreshold=0.05&_msresflags=1089&_msresflags2=2&percolate=-1&percolate_rt=0))

**202 - 219 658.9960 1973.9661 1973.9595 3 0 K.ATDTWLYGTAVGAEHNLR.L**  ([Ions score 94](http://gary/mascot/cgi/peptide_view.pl?file=../data/20130722/F001268.dat&query=483&hit=1&index=gene_63|putative&px=1&section=5&ave_thresh=3&_ignoreionsscorebelow=20&report=0&_sigthreshold=0.05&_msresflags=1089&_msresflags2=2&percolate=-1&percolate_rt=0))

**202 - 219 987.9907 1973.9668 1973.9595 4 0 K.ATDTWLYGTAVGAEHNLR.L**  ([Ions score 149](http://gary/mascot/cgi/peptide_view.pl?file=../data/20130722/F001268.dat&query=484&hit=1&index=gene_63|putative&px=1&section=5&ave_thresh=3&_ignoreionsscorebelow=20&report=0&_sigthreshold=0.05&_msresflags=1089&_msresflags2=2&percolate=-1&percolate_rt=0))

**202 - 232 791.6506 3162.5732 3162.5734 0 1 K.ATDTWLYGTAVGAEHNLRLGDSAGTAAGFIK.D**  ([Ions score 42](http://gary/mascot/cgi/peptide_view.pl?file=../data/20130722/F001268.dat&query=618&hit=1&index=gene_63|putative&px=1&section=5&ave_thresh=3&_ignoreionsscorebelow=20&report=0&_sigthreshold=0.05&_msresflags=1089&_msresflags2=2&percolate=-1&percolate_rt=0))

**220 - 232 604.3198 1206.6251 1206.6245 1 0 R.LGDSAGTAAGFIK.D**  ([Ions score 81](http://gary/mascot/cgi/peptide_view.pl?file=../data/20130722/F001268.dat&query=250&hit=1&index=gene_63|putative&px=1&section=5&ave_thresh=3&_ignoreionsscorebelow=20&report=0&_sigthreshold=0.05&_msresflags=1089&_msresflags2=2&percolate=-1&percolate_rt=0))

**233 - 247 776.3761 1550.7376 1550.7424 -3 0 K.DLSSSLGITSDEATR.L**  ([Ions score 87](http://gary/mascot/cgi/peptide_view.pl?file=../data/20130722/F001268.dat&query=396&hit=1&index=gene_63|putative&px=1&section=5&ave_thresh=3&_ignoreionsscorebelow=20&report=0&_sigthreshold=0.05&_msresflags=1089&_msresflags2=2&percolate=-1&percolate_rt=0))

**233 - 257 646.8591 2583.4074 2583.4119 -2 1 K.DLSSSLGITSDEATRLVPLLAAVQK.E**  ([Ions score 36](http://gary/mascot/cgi/peptide_view.pl?file=../data/20130722/F001268.dat&query=584&hit=1&index=gene_63|putative&px=1&section=5&ave_thresh=3&_ignoreionsscorebelow=20&report=0&_sigthreshold=0.05&_msresflags=1089&_msresflags2=2&percolate=-1&percolate_rt=0))

**233 - 257 862.1448 2583.4127 2583.4119 0 1 K.DLSSSLGITSDEATRLVPLLAAVQK.E**  ([Ions score 63](http://gary/mascot/cgi/peptide_view.pl?file=../data/20130722/F001268.dat&query=585&hit=1&index=gene_63|putative&px=1&section=5&ave_thresh=3&_ignoreionsscorebelow=20&report=0&_sigthreshold=0.05&_msresflags=1089&_msresflags2=2&percolate=-1&percolate_rt=0))

**248 - 257 526.3500 1050.6855 1050.6801 5 0 R.LVPLLAAVQK.E**  ([Ions score 31](http://gary/mascot/cgi/peptide_view.pl?file=../data/20130722/F001268.dat&query=153&hit=1&index=gene_63|putative&px=1&section=5&ave_thresh=3&_ignoreionsscorebelow=20&report=0&_sigthreshold=0.05&_msresflags=1089&_msresflags2=2&percolate=-1&percolate_rt=0))

**248 - 268 721.0977 2160.2712 2160.2630 4 1 R.LVPLLAAVQKESTPANVAALR.A**  ([Ions score 53](http://gary/mascot/cgi/peptide_view.pl?file=../data/20130722/F001268.dat&query=511&hit=1&index=gene_63|putative&px=1&section=5&ave_thresh=3&_ignoreionsscorebelow=20&report=0&_sigthreshold=0.05&_msresflags=1089&_msresflags2=2&percolate=-1&percolate_rt=0))

**258 - 268 564.8042 1127.5938 1127.5935 0 0 K.ESTPANVAALR.A**  ([Ions score 62](http://gary/mascot/cgi/peptide_view.pl?file=../data/20130722/F001268.dat&query=206&hit=1&index=gene_63|putative&px=1&section=5&ave_thresh=3&_ignoreionsscorebelow=20&report=0&_sigthreshold=0.05&_msresflags=1089&_msresflags2=2&percolate=-1&percolate_rt=0))

**274 - 286 486.2394 1455.6963 1455.6954 1 0 R.LTTAHGTENDELR.K**  ([Ions score 38](http://gary/mascot/cgi/peptide_view.pl?file=../data/20130722/F001268.dat&query=365&hit=1&index=gene_63|putative&px=1&section=5&ave_thresh=3&_ignoreionsscorebelow=20&report=0&_sigthreshold=0.05&_msresflags=1089&_msresflags2=2&percolate=-1&percolate_rt=0))

**274 - 287 396.9549 1583.7906 1583.7903 0 1 R.LTTAHGTENDELRK.L**  ([Ions score 24](http://gary/mascot/cgi/peptide_view.pl?file=../data/20130722/F001268.dat&query=403&hit=1&index=gene_63|putative&px=1&section=5&ave_thresh=3&_ignoreionsscorebelow=20&report=0&_sigthreshold=0.05&_msresflags=1089&_msresflags2=2&percolate=-1&percolate_rt=0))

**287 - 300 530.6120 1588.8142 1588.8169 -2 1 R.KLNSTLQQNATDTR.N**  ([Ions score 52](http://gary/mascot/cgi/peptide_view.pl?file=../data/20130722/F001268.dat&query=406&hit=1&index=gene_63|putative&px=1&section=5&ave_thresh=3&_ignoreionsscorebelow=20&report=0&_sigthreshold=0.05&_msresflags=1089&_msresflags2=2&percolate=-1&percolate_rt=0))

**288 - 300 731.3673 1460.7199 1460.7219 -1 0 K.LNSTLQQNATDTR.N**  ([Ions score 115](http://gary/mascot/cgi/peptide_view.pl?file=../data/20130722/F001268.dat&query=368&hit=1&index=gene_63|putative&px=1&section=5&ave_thresh=3&_ignoreionsscorebelow=20&report=0&_sigthreshold=0.05&_msresflags=1089&_msresflags2=2&percolate=-1&percolate_rt=0))

**288 - 303 592.3062 1773.8966 1773.8969 0 1 K.LNSTLQQNATDTRNAK.A**  ([Ions score 51](http://gary/mascot/cgi/peptide_view.pl?file=../data/20130722/F001268.dat&query=444&hit=1&index=gene_63|putative&px=1&section=5&ave_thresh=3&_ignoreionsscorebelow=20&report=0&_sigthreshold=0.05&_msresflags=1089&_msresflags2=2&percolate=-1&percolate_rt=0))

**304 - 318 491.2749 1470.8030 1470.8042 -1 1 K.AADDALAASKANLIK.R**  ([Ions score 21](http://gary/mascot/cgi/peptide_view.pl?file=../data/20130722/F001268.dat&query=372&hit=1&index=gene_63|putative&px=1&section=5&ave_thresh=3&_ignoreionsscorebelow=20&report=0&_sigthreshold=0.05&_msresflags=1089&_msresflags2=2&percolate=-1&percolate_rt=0))

**314 - 319 357.7347 713.4548 713.4548 0 1 K.ANLIKR.I**  ([Ions score 23](http://gary/mascot/cgi/peptide_view.pl?file=../data/20130722/F001268.dat&query=22&hit=1&index=gene_63|putative&px=1&section=5&ave_thresh=3&_ignoreionsscorebelow=20&report=0&_sigthreshold=0.05&_msresflags=1089&_msresflags2=2&percolate=-1&percolate_rt=0))

**319 - 329 657.3618 1312.7090 1312.7099 -1 1 K.RIQEQNDAIVK.N**  ([Ions score 55](http://gary/mascot/cgi/peptide_view.pl?file=../data/20130722/F001268.dat&query=325&hit=1&index=gene_63|putative&px=1&section=5&ave_thresh=3&_ignoreionsscorebelow=20&report=0&_sigthreshold=0.05&_msresflags=1089&_msresflags2=2&percolate=-1&percolate_rt=0))

**319 - 329 438.5779 1312.7117 1312.7099 1 1 K.RIQEQNDAIVK.N**  ([Ions score 59](http://gary/mascot/cgi/peptide_view.pl?file=../data/20130722/F001268.dat&query=326&hit=1&index=gene_63|putative&px=1&section=5&ave_thresh=3&_ignoreionsscorebelow=20&report=0&_sigthreshold=0.05&_msresflags=1089&_msresflags2=2&percolate=-1&percolate_rt=0))

**320 - 329 579.3106 1156.6067 1156.6088 -2 0 R.IQEQNDAIVK.N**  ([Ions score 55](http://gary/mascot/cgi/peptide_view.pl?file=../data/20130722/F001268.dat&query=220&hit=1&index=gene_63|putative&px=1&section=5&ave_thresh=3&_ignoreionsscorebelow=20&report=0&_sigthreshold=0.05&_msresflags=1089&_msresflags2=2&percolate=-1&percolate_rt=0))

**330 - 341 476.9225 1427.7455 1427.7481 -2 1 K.NQQISILADRDR.A**  ([Ions score 28](http://gary/mascot/cgi/peptide_view.pl?file=../data/20130722/F001268.dat&query=360&hit=1&index=gene_63|putative&px=1&section=5&ave_thresh=3&_ignoreionsscorebelow=20&report=0&_sigthreshold=0.05&_msresflags=1089&_msresflags2=2&percolate=-1&percolate_rt=0))

**330 - 341 714.8840 1427.7534 1427.7481 4 1 K.NQQISILADRDR.A**  ([Ions score 66](http://gary/mascot/cgi/peptide_view.pl?file=../data/20130722/F001268.dat&query=361&hit=1&index=gene_63|putative&px=1&section=5&ave_thresh=3&_ignoreionsscorebelow=20&report=0&_sigthreshold=0.05&_msresflags=1089&_msresflags2=2&percolate=-1&percolate_rt=0))

**330 - 343 407.7272 1626.8796 1626.8801 0 2 K.NQQISILADRDRAK.A**  ([Ions score 23](http://gary/mascot/cgi/peptide_view.pl?file=../data/20130722/F001268.dat&query=417&hit=1&index=gene_63|putative&px=1&section=5&ave_thresh=3&_ignoreionsscorebelow=20&report=0&_sigthreshold=0.05&_msresflags=1089&_msresflags2=2&percolate=-1&percolate_rt=0))

**351 - 356 361.2036 720.3927 720.3918 1 1 K.EAFAKR.E**  ([Ions score 29](http://gary/mascot/cgi/peptide_view.pl?file=../data/20130722/F001268.dat&query=25&hit=1&index=gene_63|putative&px=1&section=5&ave_thresh=3&_ignoreionsscorebelow=20&report=0&_sigthreshold=0.05&_msresflags=1089&_msresflags2=2&percolate=-1&percolate_rt=0))

**357 - 371 535.6183 1603.8330 1603.8318 1 1 R.EGVTKEQIAAFNAAR.D**  ([Ions score 51](http://gary/mascot/cgi/peptide_view.pl?file=../data/20130722/F001268.dat&query=411&hit=1&index=gene_63|putative&px=1&section=5&ave_thresh=3&_ignoreionsscorebelow=20&report=0&_sigthreshold=0.05&_msresflags=1089&_msresflags2=2&percolate=-1&percolate_rt=0))

**362 - 371 545.7856 1089.5566 1089.5567 0 0 K.EQIAAFNAAR.D**  ([Ions score 66](http://gary/mascot/cgi/peptide_view.pl?file=../data/20130722/F001268.dat&query=177&hit=1&index=gene_63|putative&px=1&section=5&ave_thresh=3&_ignoreionsscorebelow=20&report=0&_sigthreshold=0.05&_msresflags=1089&_msresflags2=2&percolate=-1&percolate_rt=0))

**362 - 381 740.0326 2217.0759 2217.0774 -1 1 K.EQIAAFNAARDTEAQQDIAR.I**  ([Ions score 69](http://gary/mascot/cgi/peptide_view.pl?file=../data/20130722/F001268.dat&query=522&hit=1&index=gene_63|putative&px=1&section=5&ave_thresh=3&_ignoreionsscorebelow=20&report=0&_sigthreshold=0.05&_msresflags=1089&_msresflags2=2&percolate=-1&percolate_rt=0))

**362 - 387 959.1429 2874.4070 2874.4107 -1 2 K.EQIAAFNAARDTEAQQDIARIDATEK.A**  ([Ions score 82](http://gary/mascot/cgi/peptide_view.pl?file=../data/20130722/F001268.dat&query=600&hit=1&index=gene_63|putative&px=1&section=5&ave_thresh=3&_ignoreionsscorebelow=20&report=0&_sigthreshold=0.05&_msresflags=1089&_msresflags2=2&percolate=-1&percolate_rt=0))

**372 - 381 573.7730 1145.5315 1145.5313 0 0 R.DTEAQQDIAR.I**  ([Ions score 69](http://gary/mascot/cgi/peptide_view.pl?file=../data/20130722/F001268.dat&query=214&hit=1&index=gene_63|putative&px=1&section=5&ave_thresh=3&_ignoreionsscorebelow=20&report=0&_sigthreshold=0.05&_msresflags=1089&_msresflags2=2&percolate=-1&percolate_rt=0))

**372 - 387 601.9618 1802.8635 1802.8646 -1 1 R.DTEAQQDIARIDATEK.A**  ([Ions score 35](http://gary/mascot/cgi/peptide_view.pl?file=../data/20130722/F001268.dat&query=449&hit=1&index=gene_63|putative&px=1&section=5&ave_thresh=3&_ignoreionsscorebelow=20&report=0&_sigthreshold=0.05&_msresflags=1089&_msresflags2=2&percolate=-1&percolate_rt=0))

**372 - 387 902.4449 1802.8752 1802.8646 6 1 R.DTEAQQDIARIDATEK.A**  ([Ions score 51](http://gary/mascot/cgi/peptide_view.pl?file=../data/20130722/F001268.dat&query=450&hit=1&index=gene_63|putative&px=1&section=5&ave_thresh=3&_ignoreionsscorebelow=20&report=0&_sigthreshold=0.05&_msresflags=1089&_msresflags2=2&percolate=-1&percolate_rt=0))

**388 - 399 405.8981 1214.6726 1214.6731 0 2 K.AKSDRIAAAGQK.R**  ([Ions score 40](http://gary/mascot/cgi/peptide_view.pl?file=../data/20130722/F001268.dat&query=259&hit=1&index=gene_63|putative&px=1&section=5&ave_thresh=3&_ignoreionsscorebelow=20&report=0&_sigthreshold=0.05&_msresflags=1089&_msresflags2=2&percolate=-1&percolate_rt=0))

**388 - 399 608.3436 1214.6726 1214.6731 0 2 K.AKSDRIAAAGQK.R**  ([Ions score 47](http://gary/mascot/cgi/peptide_view.pl?file=../data/20130722/F001268.dat&query=260&hit=1&index=gene_63|putative&px=1&section=5&ave_thresh=3&_ignoreionsscorebelow=20&report=0&_sigthreshold=0.05&_msresflags=1089&_msresflags2=2&percolate=-1&percolate_rt=0))

**388 - 399 304.6759 1214.6746 1214.6731 1 2 K.AKSDRIAAAGQK.R**  ([Ions score 22](http://gary/mascot/cgi/peptide_view.pl?file=../data/20130722/F001268.dat&query=261&hit=1&index=gene_63|putative&px=1&section=5&ave_thresh=3&_ignoreionsscorebelow=20&report=0&_sigthreshold=0.05&_msresflags=1089&_msresflags2=2&percolate=-1&percolate_rt=0))

**390 - 399 339.5208 1015.5405 1015.5410 -1 1 K.SDRIAAAGQK.R**  ([Ions score 47](http://gary/mascot/cgi/peptide_view.pl?file=../data/20130722/F001268.dat&query=134&hit=1&index=gene_63|putative&px=1&section=5&ave_thresh=3&_ignoreionsscorebelow=20&report=0&_sigthreshold=0.05&_msresflags=1089&_msresflags2=2&percolate=-1&percolate_rt=0))

**390 - 399 508.7780 1015.5415 1015.5410 0 1 K.SDRIAAAGQK.R**  ([Ions score 36](http://gary/mascot/cgi/peptide_view.pl?file=../data/20130722/F001268.dat&query=135&hit=1&index=gene_63|putative&px=1&section=5&ave_thresh=3&_ignoreionsscorebelow=20&report=0&_sigthreshold=0.05&_msresflags=1089&_msresflags2=2&percolate=-1&percolate_rt=0))

**400 - 406 430.2406 858.4666 858.4671 -1 1 K.RLDTQAR.Q**  ([Ions score 50](http://gary/mascot/cgi/peptide_view.pl?file=../data/20130722/F001268.dat&query=66&hit=1&index=gene_63|putative&px=1&section=5&ave_thresh=3&_ignoreionsscorebelow=20&report=0&_sigthreshold=0.05&_msresflags=1089&_msresflags2=2&percolate=-1&percolate_rt=0))

**400 - 414 553.2941 1656.8604 1656.8655 -3 2 K.RLDTQARQAETAAAR.Q**  ([Ions score 24](http://gary/mascot/cgi/peptide_view.pl?file=../data/20130722/F001268.dat&query=427&hit=1&index=gene_63|putative&px=1&section=5&ave_thresh=3&_ignoreionsscorebelow=20&report=0&_sigthreshold=0.05&_msresflags=1089&_msresflags2=2&percolate=-1&percolate_rt=0))

**407 - 414 409.2121 816.4097 816.4090 1 0 R.QAETAAAR.Q**  ([Ions score 50](http://gary/mascot/cgi/peptide_view.pl?file=../data/20130722/F001268.dat&query=54&hit=1&index=gene_63|putative&px=1&section=5&ave_thresh=3&_ignoreionsscorebelow=20&report=0&_sigthreshold=0.05&_msresflags=1089&_msresflags2=2&percolate=-1&percolate_rt=0))

**407 - 416 528.7752 1055.5359 1055.5360 0 1 R.QAETAAARQK.K**  Gln->pyro-Glu (N-term Q) ([Ions score 52](http://gary/mascot/cgi/peptide_view.pl?file=../data/20130722/F001268.dat&query=155&hit=1&index=gene_63|putative&px=1&section=5&ave_thresh=3&_ignoreionsscorebelow=20&report=0&_sigthreshold=0.05&_msresflags=1089&_msresflags2=2&percolate=-1&percolate_rt=0))

**407 - 417 395.5514 1183.6324 1183.6309 1 2 R.QAETAAARQKK.A**  Gln->pyro-Glu (N-term Q) ([Ions score 36](http://gary/mascot/cgi/peptide_view.pl?file=../data/20130722/F001268.dat&query=235&hit=1&index=gene_63|putative&px=1&section=5&ave_thresh=3&_ignoreionsscorebelow=20&report=0&_sigthreshold=0.05&_msresflags=1089&_msresflags2=2&percolate=-1&percolate_rt=0))

**407 - 417 401.2262 1200.6567 1200.6574 -1 2 R.QAETAAARQKK.A**  ([Ions score 30](http://gary/mascot/cgi/peptide_view.pl?file=../data/20130722/F001268.dat&query=245&hit=1&index=gene_63|putative&px=1&section=5&ave_thresh=3&_ignoreionsscorebelow=20&report=0&_sigthreshold=0.05&_msresflags=1089&_msresflags2=2&percolate=-1&percolate_rt=0))

**417 - 428 435.5698 1303.6877 1303.6884 -1 1 K.KAADAFLAQVDR.T**  ([Ions score 48](http://gary/mascot/cgi/peptide_view.pl?file=../data/20130722/F001268.dat&query=315&hit=1&index=gene_63|putative&px=1&section=5&ave_thresh=3&_ignoreionsscorebelow=20&report=0&_sigthreshold=0.05&_msresflags=1089&_msresflags2=2&percolate=-1&percolate_rt=0))

**418 - 428 588.8042 1175.5938 1175.5935 0 0 K.AADAFLAQVDR.T**  ([Ions score 94](http://gary/mascot/cgi/peptide_view.pl?file=../data/20130722/F001268.dat&query=229&hit=1&index=gene_63|putative&px=1&section=5&ave_thresh=3&_ignoreionsscorebelow=20&report=0&_sigthreshold=0.05&_msresflags=1089&_msresflags2=2&percolate=-1&percolate_rt=0))

**418 - 436 669.3362 2004.9869 2004.9865 0 1 K.AADAFLAQVDRTSGDEIAR.I**  ([Ions score 79](http://gary/mascot/cgi/peptide_view.pl?file=../data/20130722/F001268.dat&query=490&hit=1&index=gene_63|putative&px=1&section=5&ave_thresh=3&_ignoreionsscorebelow=20&report=0&_sigthreshold=0.05&_msresflags=1089&_msresflags2=2&percolate=-1&percolate_rt=0))

**467 - 477 601.8043 1201.5940 1201.5939 0 0 K.TDIQLAAEDAR.Q**  ([Ions score 78](http://gary/mascot/cgi/peptide_view.pl?file=../data/20130722/F001268.dat&query=246&hit=1&index=gene_63|putative&px=1&section=5&ave_thresh=3&_ignoreionsscorebelow=20&report=0&_sigthreshold=0.05&_msresflags=1089&_msresflags2=2&percolate=-1&percolate_rt=0))

**484 - 490 301.1745 900.5016 900.5002 2 2 K.RRNTQAR.Q**  ([Ions score 30](http://gary/mascot/cgi/peptide_view.pl?file=../data/20130722/F001268.dat&query=83&hit=1&index=gene_63|putative&px=1&section=5&ave_thresh=3&_ignoreionsscorebelow=20&report=0&_sigthreshold=0.05&_msresflags=1089&_msresflags2=2&percolate=-1&percolate_rt=0))

**531 - 543 527.5932 1579.7578 1579.7590 -1 1 K.GRISEQEYQDSIR.A**  ([Ions score 46](http://gary/mascot/cgi/peptide_view.pl?file=../data/20130722/F001268.dat&query=401&hit=1&index=gene_63|putative&px=1&section=5&ave_thresh=3&_ignoreionsscorebelow=20&report=0&_sigthreshold=0.05&_msresflags=1089&_msresflags2=2&percolate=-1&percolate_rt=0))

**531 - 543 790.8862 1579.7579 1579.7590 -1 1 K.GRISEQEYQDSIR.A**  ([Ions score 73](http://gary/mascot/cgi/peptide_view.pl?file=../data/20130722/F001268.dat&query=402&hit=1&index=gene_63|putative&px=1&section=5&ave_thresh=3&_ignoreionsscorebelow=20&report=0&_sigthreshold=0.05&_msresflags=1089&_msresflags2=2&percolate=-1&percolate_rt=0))

**531 - 551 624.3076 2493.2014 2493.2095 -3 2 K.GRISEQEYQDSIRAIQENTEK.K**  ([Ions score 66](http://gary/mascot/cgi/peptide_view.pl?file=../data/20130722/F001268.dat&query=557&hit=1&index=gene_63|putative&px=1&section=5&ave_thresh=3&_ignoreionsscorebelow=20&report=0&_sigthreshold=0.05&_msresflags=1089&_msresflags2=2&percolate=-1&percolate_rt=0))

**533 - 543 684.3251 1366.6357 1366.6364 -1 0 R.ISEQEYQDSIR.A**  ([Ions score 86](http://gary/mascot/cgi/peptide_view.pl?file=../data/20130722/F001268.dat&query=343&hit=1&index=gene_63|putative&px=1&section=5&ave_thresh=3&_ignoreionsscorebelow=20&report=0&_sigthreshold=0.05&_msresflags=1089&_msresflags2=2&percolate=-1&percolate_rt=0))

**533 - 551 761.0388 2280.0946 2280.0869 3 1 R.ISEQEYQDSIRAIQENTEK.K**  ([Ions score 65](http://gary/mascot/cgi/peptide_view.pl?file=../data/20130722/F001268.dat&query=528&hit=1&index=gene_63|putative&px=1&section=5&ave_thresh=3&_ignoreionsscorebelow=20&report=0&_sigthreshold=0.05&_msresflags=1089&_msresflags2=2&percolate=-1&percolate_rt=0))

**544 - 551 466.7377 931.4609 931.4610 0 0 R.AIQENTEK.K**  ([Ions score 30](http://gary/mascot/cgi/peptide_view.pl?file=../data/20130722/F001268.dat&query=100&hit=1&index=gene_63|putative&px=1&section=5&ave_thresh=3&_ignoreionsscorebelow=20&report=0&_sigthreshold=0.05&_msresflags=1089&_msresflags2=2&percolate=-1&percolate_rt=0))

**552 - 569 674.3600 2020.0581 2020.0510 4 2 K.KKEEVQLAALSDMTSNLK.T**  Oxidation (M) ([Ions score 84](http://gary/mascot/cgi/peptide_view.pl?file=../data/20130722/F001268.dat&query=495&hit=1&index=gene_63|putative&px=1&section=5&ave_thresh=3&_ignoreionsscorebelow=20&report=0&_sigthreshold=0.05&_msresflags=1089&_msresflags2=2&percolate=-1&percolate_rt=0))

**570 - 580 568.8003 1135.5861 1135.5873 -1 0 K.TALGEGNALYK.A**  ([Ions score 72](http://gary/mascot/cgi/peptide_view.pl?file=../data/20130722/F001268.dat&query=209&hit=1&index=gene_63|putative&px=1&section=5&ave_thresh=3&_ignoreionsscorebelow=20&report=0&_sigthreshold=0.05&_msresflags=1089&_msresflags2=2&percolate=-1&percolate_rt=0))

**570 - 580 569.2927 1136.5708 1136.5713 0 0 K.TALGEGNALYK.A**  Deamidated (NQ) ([Ions score 63](http://gary/mascot/cgi/peptide_view.pl?file=../data/20130722/F001268.dat&query=211&hit=1&index=gene_63|putative&px=1&section=5&ave_thresh=3&_ignoreionsscorebelow=20&report=0&_sigthreshold=0.05&_msresflags=1089&_msresflags2=2&percolate=-1&percolate_rt=0))

**581 - 593 704.8875 1407.7605 1407.7609 0 0 K.AAAITQTIIDTYK.G**  ([Ions score 95](http://gary/mascot/cgi/peptide_view.pl?file=../data/20130722/F001268.dat&query=354&hit=1&index=gene_63|putative&px=1&section=5&ave_thresh=3&_ignoreionsscorebelow=20&report=0&_sigthreshold=0.05&_msresflags=1089&_msresflags2=2&percolate=-1&percolate_rt=0))

**594 - 627 991.8605 2972.5596 2972.5542 2 0 K.GATAAYSAFAGIPIVGPALGAAAAAAAIGAGMAR.V**

Oxidation (M) ([Ions score 84](http://gary/mascot/cgi/peptide_view.pl?file=../data/20130722/F001268.dat&query=607&hit=1&index=gene_63|putative&px=1&section=5&ave_thresh=3&_ignoreionsscorebelow=20&report=0&_sigthreshold=0.05&_msresflags=1089&_msresflags2=2&percolate=-1&percolate_rt=0))

**7.** [gene_68|](http://10.139.25.109/mascot/cgi/protein_view.pl?file=../data/20100316/F010069.dat&hit=gene_68|conserved&px=1&ave_thresh=1&_sigthreshold=0.05&_server_mudpit_switch=1e-009&_ignoreionsscorebelow=20)putative tail protein |[Serratia phage Eta] **Mass:** 93247    **Score:** 1789   **Queries matched:** 46(46)

**emPAI:** 3.90; Sequence Coverage: **39%;** Matched peptides shown in **Bold Red**

**1** MALVIHYTRN ENGGFNTSQH YTEPMLFVTE RIPDGVPFTI YRDSVGEDNN VTENFDALRE PGIFHIIEGA GGGVVSGAFK LLGAVLSPIM K**LLTPSTAAA**

**101 QSFSNVQGES PNNSLTDR**NN KPRPYER**VYD ICGTVQSIPN NLMTTYSVFN SSGR**KVEYGY YCVGRSYLDI SVSGVTDGDT LLQNIPGASA YIYGPNTSPN

**201** SGSPQLAIGD PIDQGLYSTV SANEVDGQTL K**APNDLSSR**M TDGATAVYSG GLGTITDPSG GADFSAYIK**V GSNVVFK**NVF VDGVADLNAI YVVTDVSSVS

**301** ITVNPSPK**LD QWVNVGGTPR PLKLSDDPIV EPYDTKEASF TPWVSITRLQ PER**LLINVSA PNGMYLDWGA GKLQLGTTFE AEYQLLDELN NPYGPVYAQQ

**401** ATVTGK**SSQE TGATLYAVLP VASKVR**VRVR RVTPFQFWYN GVVVDELKYN ELFAQVTDYT PHYGNITTVH TAR**LQTVRAT AVK**QPQLK**IL CTEMVQKYLG**

**501 AGQFDATWTP NTQAVQSMIR** LLRDPVVGGL DLTTANMDAL LATQTEIETY FNDVRAGQFC YTFDTYTTTA QDIIEAIAEA VFCRAYREGK **SILLDFDRPR**

**601 VGPEMVFTHR SKIPGQEKWT RSFDDQSSYD SLKFSYIDPK K**NTK**ETITIP ADGGRKTYTY DSK**GVR**NYQQ AYWLAHR**RYQ MIKLKRISVD FSATEEGSLV

**701** RPGR**AISVVK** GSRVAPYDGY IVAADGLTLT LSQEVDFSGS GQHSIILK**KR DGGVQSVPVV AGTTKR**K**VVM LAAPQEAVYT GNSALKTEFS FGSDSR**HDAQ

**801** MILVQTVTPN DDRTVK**ITGF NYHQDYYMQD GVTPFGR**AFS NGFDTGFS

**Start - End Observed Mr(expt) Mr(calc) ppm Miss Sequence**

**92 - 118 935.7930 2804.3571 2804.3577 0 0 K.LLTPSTAAAQSFSNVQGESPNNSLTDR.N**  ([Ions score 79](http://gary/mascot/cgi/peptide_view.pl?file=../data/20130722/F001269.dat&query=983&hit=1&index=gene_68|conserved&px=1&section=5&ave_thresh=2&_ignoreionsscorebelow=20&report=0&_sigthreshold=0.05&_msresflags=1089&_msresflags2=2&percolate=-1&percolate_rt=0))

**128 - 154 1008.4771 3022.4095 3022.4165 -2 0 R.VYDICGTVQSIPNNLMTTYSVFNSSGR.K**  ([Ions score 54](http://gary/mascot/cgi/peptide_view.pl?file=../data/20130722/F001269.dat&query=1009&hit=1&index=gene_68|conserved&px=1&section=5&ave_thresh=2&_ignoreionsscorebelow=20&report=0&_sigthreshold=0.05&_msresflags=1089&_msresflags2=2&percolate=-1&percolate_rt=0))

**128 - 154 1013.8135 3038.4188 3038.4114 2 0 R.VYDICGTVQSIPNNLMTTYSVFNSSGR.K**  Oxidation(M)([Ions score 38](http://gary/mascot/cgi/peptide_view.pl?file=../data/20130722/F001269.dat&query=1014&hit=1&index=gene_68|conserved&px=1&section=5&ave_thresh=2&_ignoreionsscorebelow=20&report=0&_sigthreshold=0.05&_msresflags=1089&_msresflags2=2&percolate=-1&percolate_rt=0))

**232 - 239 430.2170 858.4195 858.4195 0 0 K.APNDLSSR.M**  ([Ions score 49](http://gary/mascot/cgi/peptide_view.pl?file=../data/20130722/F001269.dat&query=91&hit=1&index=gene_68|conserved&px=1&section=5&ave_thresh=2&_ignoreionsscorebelow=20&report=0&_sigthreshold=0.05&_msresflags=1089&_msresflags2=2&percolate=-1&percolate_rt=0))

**232 - 239 430.7077 859.4008 859.4035 -3 0 K.APNDLSSR.M**  Deamidated (NQ) ([Ions score 39](http://gary/mascot/cgi/peptide_view.pl?file=../data/20130722/F001269.dat&query=95&hit=1&index=gene_68|conserved&px=1&section=5&ave_thresh=2&_ignoreionsscorebelow=20&report=0&_sigthreshold=0.05&_msresflags=1089&_msresflags2=2&percolate=-1&percolate_rt=0))

**270 - 277 425.2447 848.4749 848.4756 -1 0 K.VGSNVVFK.N**  ([Ions score 65](http://gary/mascot/cgi/peptide_view.pl?file=../data/20130722/F001269.dat&query=87&hit=1&index=gene_68|conserved&px=1&section=5&ave_thresh=2&_ignoreionsscorebelow=20&report=0&_sigthreshold=0.05&_msresflags=1089&_msresflags2=2&percolate=-1&percolate_rt=0))

**270 - 277 425.7355 849.4564 849.4596 -4 0 K.VGSNVVFK.N**  Deamidated (NQ) ([Ions score 58](http://gary/mascot/cgi/peptide_view.pl?file=../data/20130722/F001269.dat&query=88&hit=1&index=gene_68|conserved&px=1&section=5&ave_thresh=2&_ignoreionsscorebelow=20&report=0&_sigthreshold=0.05&_msresflags=1089&_msresflags2=2&percolate=-1&percolate_rt=0))

**309 - 323 840.4638 1678.9131 1678.9155 -1 0 K.LDQWVNVGGTPRPLK.L**  ([Ions score 46](http://gary/mascot/cgi/peptide_view.pl?file=../data/20130722/F001269.dat&query=739&hit=1&index=gene_68|conserved&px=1&section=5&ave_thresh=2&_ignoreionsscorebelow=20&report=0&_sigthreshold=0.05&_msresflags=1089&_msresflags2=2&percolate=-1&percolate_rt=0))

**309 - 323 560.6461 1678.9164 1678.9155 1 0 K.LDQWVNVGGTPRPLK.L**  ([Ions score 44](http://gary/mascot/cgi/peptide_view.pl?file=../data/20130722/F001269.dat&query=740&hit=1&index=gene_68|conserved&px=1&section=5&ave_thresh=2&_ignoreionsscorebelow=20&report=0&_sigthreshold=0.05&_msresflags=1089&_msresflags2=2&percolate=-1&percolate_rt=0))

**324 - 336 497.9116 1490.7130 1490.7141 -1 0 K.LSDDPIVEPYDTK.E**  ([Ions score 22](http://gary/mascot/cgi/peptide_view.pl?file=../data/20130722/F001269.dat&query=615&hit=1&index=gene_68|conserved&px=1&section=5&ave_thresh=2&_ignoreionsscorebelow=20&report=0&_sigthreshold=0.05&_msresflags=1089&_msresflags2=2&percolate=-1&percolate_rt=0))

**324 - 336 746.3641 1490.7137 1490.7141 0 0 K.LSDDPIVEPYDTK.E**  ([Ions score 79](http://gary/mascot/cgi/peptide_view.pl?file=../data/20130722/F001269.dat&query=616&hit=1&index=gene_68|conserved&px=1&section=5&ave_thresh=2&_ignoreionsscorebelow=20&report=0&_sigthreshold=0.05&_msresflags=1089&_msresflags2=2&percolate=-1&percolate_rt=0))

**324 - 348 956.1442 2865.4107 2865.4073 1 1 K.LSDDPIVEPYDTKEASFTPWVSITR.L**  ([Ions score 73](http://gary/mascot/cgi/peptide_view.pl?file=../data/20130722/F001269.dat&query=986&hit=1&index=gene_68|conserved&px=1&section=5&ave_thresh=2&_ignoreionsscorebelow=20&report=0&_sigthreshold=0.05&_msresflags=1089&_msresflags2=2&percolate=-1&percolate_rt=0))

**337 - 348 697.3591 1392.7036 1392.7038 0 0 K.EASFTPWVSITR.L**  ([Ions score 70](http://gary/mascot/cgi/peptide_view.pl?file=../data/20130722/F001269.dat&query=551&hit=1&index=gene_68|conserved&px=1&section=5&ave_thresh=2&_ignoreionsscorebelow=20&report=0&_sigthreshold=0.05&_msresflags=1089&_msresflags2=2&percolate=-1&percolate_rt=0))

**337 - 353 673.0225 2016.0456 2016.0429 1 1 K.EASFTPWVSITRLQPER.L**  ([Ions score 35](http://gary/mascot/cgi/peptide_view.pl?file=../data/20130722/F001269.dat&query=841&hit=1&index=gene_68|conserved&px=1&section=5&ave_thresh=2&_ignoreionsscorebelow=20&report=0&_sigthreshold=0.05&_msresflags=1089&_msresflags2=2&percolate=-1&percolate_rt=0))

**407 - 424 607.9911 1820.9514 1820.9520 0 0 K.SSQETGATLYAVLPVASK.V**  ([Ions score 59](http://gary/mascot/cgi/peptide_view.pl?file=../data/20130722/F001269.dat&query=775&hit=1&index=gene_68|conserved&px=1&section=5&ave_thresh=2&_ignoreionsscorebelow=20&report=0&_sigthreshold=0.05&_msresflags=1089&_msresflags2=2&percolate=-1&percolate_rt=0))

**407 - 424 911.4868 1820.9590 1820.9520 4 0 K.SSQETGATLYAVLPVASK.V**  ([Ions score 103](http://gary/mascot/cgi/peptide_view.pl?file=../data/20130722/F001269.dat&query=776&hit=1&index=gene_68|conserved&px=1&section=5&ave_thresh=2&_ignoreionsscorebelow=20&report=0&_sigthreshold=0.05&_msresflags=1089&_msresflags2=2&percolate=-1&percolate_rt=0))

**407 - 426 693.0463 2076.1172 2076.1215 -2 1 K.SSQETGATLYAVLPVASKVR.V**  ([Ions score 49](http://gary/mascot/cgi/peptide_view.pl?file=../data/20130722/F001269.dat&query=859&hit=1&index=gene_68|conserved&px=1&section=5&ave_thresh=2&_ignoreionsscorebelow=20&report=0&_sigthreshold=0.05&_msresflags=1089&_msresflags2=2&percolate=-1&percolate_rt=0))

**407 - 426 1039.0706 2076.1266 2076.1215 2 1 K.SSQETGATLYAVLPVASKVR.V**  ([Ions score 115](http://gary/mascot/cgi/peptide_view.pl?file=../data/20130722/F001269.dat&query=860&hit=1&index=gene_68|conserved&px=1&section=5&ave_thresh=2&_ignoreionsscorebelow=20&report=0&_sigthreshold=0.05&_msresflags=1089&_msresflags2=2&percolate=-1&percolate_rt=0))

**474 - 483 543.8342 1085.6538 1085.6557 -2 1 R.LQTVRATAVK.Q**  ([Ions score 43](http://gary/mascot/cgi/peptide_view.pl?file=../data/20130722/F001269.dat&query=262&hit=1&index=gene_68|conserved&px=1&section=5&ave_thresh=2&_ignoreionsscorebelow=20&report=0&_sigthreshold=0.05&_msresflags=1089&_msresflags2=2&percolate=-1&percolate_rt=0))

**489 - 497 561.2882 1120.5619 1120.5620 0 0 K.ILCTEMVQK.Y**  ([Ions score 53](http://gary/mascot/cgi/peptide_view.pl?file=../data/20130722/F001269.dat&query=307&hit=1&index=gene_68|conserved&px=1&section=5&ave_thresh=2&_ignoreionsscorebelow=20&report=0&_sigthreshold=0.05&_msresflags=1089&_msresflags2=2&percolate=-1&percolate_rt=0))

**489 - 497 569.2866 1136.5586 1136.5570 1 0 K.ILCTEMVQK.Y**  Oxidation (M) ([Ions score 58](http://gary/mascot/cgi/peptide_view.pl?file=../data/20130722/F001269.dat&query=330&hit=1&index=gene_68|conserved&px=1&section=5&ave_thresh=2&_ignoreionsscorebelow=20&report=0&_sigthreshold=0.05&_msresflags=1089&_msresflags2=2&percolate=-1&percolate_rt=0))

**498 - 520 852.4164 2554.2275 2554.2275 0 0 K.YLGAGQFDATWTPNTQAVQSMIR.L**  ([Ions score 57](http://gary/mascot/cgi/peptide_view.pl?file=../data/20130722/F001269.dat&query=949&hit=1&index=gene_68|conserved&px=1&section=5&ave_thresh=2&_ignoreionsscorebelow=20&report=0&_sigthreshold=0.05&_msresflags=1089&_msresflags2=2&percolate=-1&percolate_rt=0))

**498 - 520 857.7490 2570.2251 2570.2224 1 0 K.YLGAGQFDATWTPNTQAVQSMIR.L**  Oxidation (M) ([Ions score 68](http://gary/mascot/cgi/peptide_view.pl?file=../data/20130722/F001269.dat&query=955&hit=1&index=gene_68|conserved&px=1&section=5&ave_thresh=2&_ignoreionsscorebelow=20&report=0&_sigthreshold=0.05&_msresflags=1089&_msresflags2=2&percolate=-1&percolate_rt=0))

**591 - 600 616.3443 1230.6740 1230.6721 2 0 K.SILLDFDRPR.V**  ([Ions score 38](http://gary/mascot/cgi/peptide_view.pl?file=../data/20130722/F001269.dat&query=425&hit=1&index=gene_68|conserved&px=1&section=5&ave_thresh=2&_ignoreionsscorebelow=20&report=0&_sigthreshold=0.05&_msresflags=1089&_msresflags2=2&percolate=-1&percolate_rt=0))

**601 - 610 586.7966 1171.5786 1171.5808 -2 0 R.VGPEMVFTHR.S**  ([Ions score 57](http://gary/mascot/cgi/peptide_view.pl?file=../data/20130722/F001269.dat&query=367&hit=1&index=gene_68|conserved&px=1&section=5&ave_thresh=2&_ignoreionsscorebelow=20&report=0&_sigthreshold=0.05&_msresflags=1089&_msresflags2=2&percolate=-1&percolate_rt=0))

**601 - 610 391.5348 1171.5825 1171.5808 1 0 R.VGPEMVFTHR.S**  ([Ions score 46](http://gary/mascot/cgi/peptide_view.pl?file=../data/20130722/F001269.dat&query=368&hit=1&index=gene_68|conserved&px=1&section=5&ave_thresh=2&_ignoreionsscorebelow=20&report=0&_sigthreshold=0.05&_msresflags=1089&_msresflags2=2&percolate=-1&percolate_rt=0))

**601 - 610 396.8656 1187.5749 1187.5757 -1 0 R.VGPEMVFTHR.S**  Oxidation (M) ([Ions score 34](http://gary/mascot/cgi/peptide_view.pl?file=../data/20130722/F001269.dat&query=381&hit=1&index=gene_68|conserved&px=1&section=5&ave_thresh=2&_ignoreionsscorebelow=20&report=0&_sigthreshold=0.05&_msresflags=1089&_msresflags2=2&percolate=-1&percolate_rt=0))

**601 - 610 594.7948 1187.5750 1187.5757 -1 0 R.VGPEMVFTHR.S**  Oxidation (M) ([Ions score 70](http://gary/mascot/cgi/peptide_view.pl?file=../data/20130722/F001269.dat&query=382&hit=1&index=gene_68|conserved&px=1&section=5&ave_thresh=2&_ignoreionsscorebelow=20&report=0&_sigthreshold=0.05&_msresflags=1089&_msresflags2=2&percolate=-1&percolate_rt=0))

**611 - 618 443.7530 885.4915 885.4920 -1 1 R.SKIPGQEK.W**  ([Ions score 33](http://gary/mascot/cgi/peptide_view.pl?file=../data/20130722/F001269.dat&query=110&hit=1&index=gene_68|conserved&px=1&section=5&ave_thresh=2&_ignoreionsscorebelow=20&report=0&_sigthreshold=0.05&_msresflags=1089&_msresflags2=2&percolate=-1&percolate_rt=0))

**619 - 633 612.2794 1833.8164 1833.8170 0 1 K.WTRSFDDQSSYDSLK.F**  ([Ions score 34](http://gary/mascot/cgi/peptide_view.pl?file=../data/20130722/F001269.dat&query=780&hit=1&index=gene_68|conserved&px=1&section=5&ave_thresh=2&_ignoreionsscorebelow=20&report=0&_sigthreshold=0.05&_msresflags=1089&_msresflags2=2&percolate=-1&percolate_rt=0))

**622 - 633 696.3017 1390.5888 1390.5889 0 0 R.SFDDQSSYDSLK.F**  ([Ions score 75](http://gary/mascot/cgi/peptide_view.pl?file=../data/20130722/F001269.dat&query=549&hit=1&index=gene_68|conserved&px=1&section=5&ave_thresh=2&_ignoreionsscorebelow=20&report=0&_sigthreshold=0.05&_msresflags=1089&_msresflags2=2&percolate=-1&percolate_rt=0))

**634 - 640 435.2245 868.4344 868.4331 2 0 K.FSYIDPK.K**  ([Ions score 23](http://gary/mascot/cgi/peptide_view.pl?file=../data/20130722/F001269.dat&query=99&hit=1&index=gene_68|conserved&px=1&section=5&ave_thresh=2&_ignoreionsscorebelow=20&report=0&_sigthreshold=0.05&_msresflags=1089&_msresflags2=2&percolate=-1&percolate_rt=0))

**634 - 641 499.2713 996.5281 996.5280 0 1 K.FSYIDPKK.N**  ([Ions score 33](http://gary/mascot/cgi/peptide_view.pl?file=../data/20130722/F001269.dat&query=184&hit=1&index=gene_68|conserved&px=1&section=5&ave_thresh=2&_ignoreionsscorebelow=20&report=0&_sigthreshold=0.05&_msresflags=1089&_msresflags2=2&percolate=-1&percolate_rt=0))

**634 - 641 333.1834 996.5282 996.5280 0 1 K.FSYIDPKK.N**  ([Ions score 28](http://gary/mascot/cgi/peptide_view.pl?file=../data/20130722/F001269.dat&query=185&hit=1&index=gene_68|conserved&px=1&section=5&ave_thresh=2&_ignoreionsscorebelow=20&report=0&_sigthreshold=0.05&_msresflags=1089&_msresflags2=2&percolate=-1&percolate_rt=0))

**645 - 655 565.2959 1128.5772 1128.5775 0 0 K.ETITIPADGGR.K**  ([Ions score 56](http://gary/mascot/cgi/peptide_view.pl?file=../data/20130722/F001269.dat&query=321&hit=1&index=gene_68|conserved&px=1&section=5&ave_thresh=2&_ignoreionsscorebelow=20&report=0&_sigthreshold=0.05&_msresflags=1089&_msresflags2=2&percolate=-1&percolate_rt=0))

**645 - 656 629.3423 1256.6700 1256.6725 -2 1 K.ETITIPADGGRK.T**  ([Ions score 38](http://gary/mascot/cgi/peptide_view.pl?file=../data/20130722/F001269.dat&query=448&hit=1&index=gene_68|conserved&px=1&section=5&ave_thresh=2&_ignoreionsscorebelow=20&report=0&_sigthreshold=0.05&_msresflags=1089&_msresflags2=2&percolate=-1&percolate_rt=0))

**657 - 663 439.2003 876.3861 876.3865 0 0 K.TYTYDSK.G**  ([Ions score 28](http://gary/mascot/cgi/peptide_view.pl?file=../data/20130722/F001269.dat&query=104&hit=1&index=gene_68|conserved&px=1&section=5&ave_thresh=2&_ignoreionsscorebelow=20&report=0&_sigthreshold=0.05&_msresflags=1089&_msresflags2=2&percolate=-1&percolate_rt=0))

**667 - 677 483.9045 1448.6916 1448.6949 -2 0 R.NYQQAYWLAHR.R**  ([Ions score 29](http://gary/mascot/cgi/peptide_view.pl?file=../data/20130722/F001269.dat&query=584&hit=1&index=gene_68|conserved&px=1&section=5&ave_thresh=2&_ignoreionsscorebelow=20&report=0&_sigthreshold=0.05&_msresflags=1089&_msresflags2=2&percolate=-1&percolate_rt=0))

**705 - 710 308.7049 615.3953 615.3956 0 0 R.AISVVK.G**  ([Ions score 45](http://gary/mascot/cgi/peptide_view.pl?file=../data/20130722/F001269.dat&query=1&hit=1&index=gene_68|conserved&px=1&section=5&ave_thresh=2&_ignoreionsscorebelow=20&report=0&_sigthreshold=0.05&_msresflags=1089&_msresflags2=2&percolate=-1&percolate_rt=0))

**749 - 765 566.9877 1697.9412 1697.9425 -1 2 K.KRDGGVQSVPVVAGTTK.R**  ([Ions score 28](http://gary/mascot/cgi/peptide_view.pl?file=../data/20130722/F001269.dat&query=743&hit=1&index=gene_68|conserved&px=1&section=5&ave_thresh=2&_ignoreionsscorebelow=20&report=0&_sigthreshold=0.05&_msresflags=1089&_msresflags2=2&percolate=-1&percolate_rt=0))

**751 - 765 707.8796 1413.7446 1413.7464 -1 0 R.DGGVQSVPVVAGTTK.R**  ([Ions score 82](http://gary/mascot/cgi/peptide_view.pl?file=../data/20130722/F001269.dat&query=566&hit=1&index=gene_68|conserved&px=1&section=5&ave_thresh=2&_ignoreionsscorebelow=20&report=0&_sigthreshold=0.05&_msresflags=1089&_msresflags2=2&percolate=-1&percolate_rt=0))

**751 - 766 785.9298 1569.8451 1569.8475 -2 1 R.DGGVQSVPVVAGTTKR.K**  ([Ions score 53](http://gary/mascot/cgi/peptide_view.pl?file=../data/20130722/F001269.dat&query=674&hit=1&index=gene_68|conserved&px=1&section=5&ave_thresh=2&_ignoreionsscorebelow=20&report=0&_sigthreshold=0.05&_msresflags=1089&_msresflags2=2&percolate=-1&percolate_rt=0))

**751 - 766 524.2911 1569.8516 1569.8475 3 1 R.DGGVQSVPVVAGTTKR.K**  ([Ions score 22](http://gary/mascot/cgi/peptide_view.pl?file=../data/20130722/F001269.dat&query=675&hit=1&index=gene_68|conserved&px=1&section=5&ave_thresh=2&_ignoreionsscorebelow=20&report=0&_sigthreshold=0.05&_msresflags=1089&_msresflags2=2&percolate=-1&percolate_rt=0))

**768 - 786 654.6846 1961.0321 1961.0292 1 0 K.VVMLAAPQEAVYTGNSALK.T**  ([Ions score 70](http://gary/mascot/cgi/peptide_view.pl?file=../data/20130722/F001269.dat&query=824&hit=1&index=gene_68|conserved&px=1&section=5&ave_thresh=2&_ignoreionsscorebelow=20&report=0&_sigthreshold=0.05&_msresflags=1089&_msresflags2=2&percolate=-1&percolate_rt=0))

**787 - 796 566.7493 1131.4841 1131.4833 1 0 K.TEFSFGSDSR.H**  ([Ions score 46](http://gary/mascot/cgi/peptide_view.pl?file=../data/20130722/F001269.dat&query=323&hit=1&index=gene_68|conserved&px=1&section=5&ave_thresh=2&_ignoreionsscorebelow=20&report=0&_sigthreshold=0.05&_msresflags=1089&_msresflags2=2&percolate=-1&percolate_rt=0))

**817 - 837 842.3795 2524.1166 2524.1118 2 0 K.ITGFNYHQDYYMQDGVTPFGR.A**  Oxidation (M) ([Ions score 50](http://gary/mascot/cgi/peptide_view.pl?file=../data/20130722/F001269.dat&query=930&hit=1&index=gene_68|conserved&px=1&section=5&ave_thresh=2&_ignoreionsscorebelow=20&report=0&_sigthreshold=0.05&_msresflags=1089&_msresflags2=2&percolate=-1&percolate_rt=0))

**C. Modification of N-terminus of tailspike proteins by acetylation**

MS/MS Fragmentation of **SSGCGDVLSLEDLKTAK** at m/z 911.4507(2+) 
[gene_69|putative](http://10.139.25.109/mascot/cgi/protein_view.pl?file=../data/20100316/F010067.dat&hit=gene_69|conserved&px=1&ave_thresh=1&_sigthreshold=0.05&_server_mudpit_switch=1e-009&_ignoreionsscorebelow=20) tailspike protein|[Serratia phage Eta]

Bottom of Form

**Monoisotopic mass of neutral peptide Mr(calc):** 1820.8826

**Fixed modifications:** Carbamidomethyl (C) (apply to specified residues or termini only)

**Variable modifications:**

**N-term :** Acetyl (Protein N-term)

**Ions Score:** 104 **Expect:** 4.1e-011

**
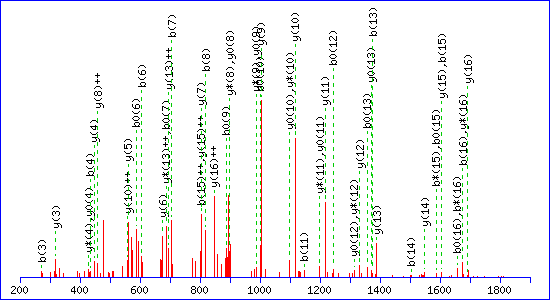
**
